# Invigorating human MSCs for transplantation therapy via Nrf2/DKK1 co-stimulation in an acute-on-chronic liver failure mouse model

**DOI:** 10.1093/gastro/goae016

**Published:** 2024-03-25

**Authors:** Feng Chen, Zhaodi Che, Yingxia Liu, Pingping Luo, Lu Xiao, Yali Song, Cunchuan Wang, Zhiyong Dong, Mianhuan Li, George L Tipoe, Min Yang, Yi Lv, Hong Zhang, Fei Wang, Jia Xiao

**Affiliations:** Division of Gastroenterology, Seventh Affiliated Hospital of Sun Yat-sen University, Shenzhen, Guangdong, P. R. China; National Clinical Research Center for Infectious Diseases, Second Affiliated Hospital of Southern University of Science and Technology, Shenzhen, Guangdong, P. R. China; Clinical Medicine Research Institute and Department of Metabolic and Bariatric Surgery, The First Affiliated Hospital of Jinan University, Guangzhou, Guangdong, P. R. China; National Clinical Research Center for Infectious Diseases, Second Affiliated Hospital of Southern University of Science and Technology, Shenzhen, Guangdong, P. R. China; Clinical Medicine Research Institute and Department of Metabolic and Bariatric Surgery, The First Affiliated Hospital of Jinan University, Guangzhou, Guangdong, P. R. China; Clinical Medicine Research Institute and Department of Metabolic and Bariatric Surgery, The First Affiliated Hospital of Jinan University, Guangzhou, Guangdong, P. R. China; Clinical Medicine Research Institute and Department of Metabolic and Bariatric Surgery, The First Affiliated Hospital of Jinan University, Guangzhou, Guangdong, P. R. China; Clinical Medicine Research Institute and Department of Metabolic and Bariatric Surgery, The First Affiliated Hospital of Jinan University, Guangzhou, Guangdong, P. R. China; Clinical Medicine Research Institute and Department of Metabolic and Bariatric Surgery, The First Affiliated Hospital of Jinan University, Guangzhou, Guangdong, P. R. China; National Clinical Research Center for Infectious Diseases, Second Affiliated Hospital of Southern University of Science and Technology, Shenzhen, Guangdong, P. R. China; School of Biomedical Sciences, The University of Hong Kong, Hong Kong SAR, P. R. China; National Clinical Research Center for Infectious Diseases, Second Affiliated Hospital of Southern University of Science and Technology, Shenzhen, Guangdong, P. R. China; Laboratory of Neuroendocrinology, Fujian Key Laboratory of Developmental and Neurobiology, School of Life Sciences, Fujian Normal University, Fuzhou, Fujian, P. R. China; Department of Surgery, The Sixth Affiliated Hospital of Jinan University, Jinan University, Dongguan, Guangdong, P. R. China; Division of Gastroenterology, Seventh Affiliated Hospital of Sun Yat-sen University, Shenzhen, Guangdong, P. R. China; Clinical Medicine Research Institute and Department of Metabolic and Bariatric Surgery, The First Affiliated Hospital of Jinan University, Guangzhou, Guangdong, P. R. China; Department of Surgery, The Sixth Affiliated Hospital of Jinan University, Jinan University, Dongguan, Guangdong, P. R. China

**Keywords:** mesenchymal stromal cells, acute-on-chronic liver failure, Nrf2/DKK1, CKAP4, oxidative stress

## Abstract

**Background:**

Since boosting stem cell resilience in stressful environments is critical for the therapeutic efficacy of stem cell-based transplantations in liver disease, this study aimed to establish the efficacy of a transient plasmid-based preconditioning strategy for boosting the capability of mesenchymal stromal cells (MSCs) for anti-inflammation/antioxidant defenses and paracrine actions in recipient hepatocytes.

**Methods:**

Human adipose mesenchymal stem cells (hADMSCs) were subjected to transfer, either with or without the nuclear factor erythroid 2-related factor 2 (Nrf2)/Dickkopf1 (DKK1) genes, followed by exposure to TNF-α/H_2_O_2_. Mouse models were subjected to acute chronic liver failure (ACLF) and subsequently injected with either transfected or untransfected MSCs. These hADMSCs and ACLF mouse models were used to investigate the interaction between Nrf2/DKK1 and the hepatocyte receptor cytoskeleton-associated protein 4 (CKAP4).

**Results:**

Activation of Nrf2 and DKK1 enhanced the anti-stress capacity of MSCs *in vitro*. In a murine model of ACLF, transient co-overexpression of Nrf2 and DKK1 via plasmid transfection improved MSC resilience against inflammatory and oxidative assaults, boosted MSC transplantation efficacy, and promoted recipient liver regeneration due to a shift from the activation of the anti-regenerative IFN-γ/STAT1 pathway to the pro-regenerative IL-6/STAT3 pathway in the liver. Importantly, the therapeutic benefits of MSC transplantation were nullified when the receptor CKAP4, which interacts with DKK1, was specifically removed from recipient hepatocytes. However, the removal of the another receptor low-density lipoprotein receptor-related protein 6 (LRP6) had no impact on the effectiveness of MSC transplantation. Moreover, in long-term observations, no tumorigenicity was detected in mice following transplantation of transiently preconditioned MSCs.

**Conclusions:**

Co-stimulation with Nrf2/DKK1 safely improved the efficacy of human MSC-based therapies in murine models of ACLF through CKAP4-dependent paracrine mechanisms.

## Introduction

As a vital organ governing systemic homeostasis, the liver is continually exposed to biological and/or chemical toxins, some of which have profound implications in severe liver diseases [1]. Acute-on-chronic liver failure (ACLF) is a clinical syndrome that occurs when there is an acute liver insult on top of pre-existing chronic liver injury. This condition is marked by a sudden decline in liver function, leading to a rapid deterioration of overall liver health and a high risk of short-term mortality [2, 3]. The main etiologies for acute liver insult include alcohol drinking, bacterial infections, viral hepatitis (e.g. hepatitis B virus), and drug-induced liver injury, while the most frequently documented etiologies for the pre-existing chronic liver injury of ACLF include chronic alcohol consumption and hepatitis B virus infection [4–7]. Existing management strategies focus primarily on providing organ support and resolving complications, and there are no specific drugs for patients with acute-on-chronic liver failure (ACLF). Liver transplantation is often the only curative treatment for ACLF patients with organ failure in the presence of cirrhosis, especially extrahepatic organ failure. However, the lack of donors makes the clinical achievement of liver transplantation difficult [8]. Thus, there is a need to develop alternative approaches to support spontaneous liver regeneration and prepare the liver for transplantation.

In recent years, human mesenchymal stromal cell (MSC) transplantation has been explored as a treatment option for ACLF in experimental models and patients [9–12]. Although still in their formative stages, MSC-based treatments have generally shown encouraging results. In these treatments, MSCs improved the condition of the recipients’ liver by boosting the hepatic regenerative capacity and exerting immunoregulatory effects through their paracrine actions on local hepatocytes [13, 14]. However, a major challenge in achieving efficacious MSC-based therapies in the clinic is the poor survival rate of MSCs after transplantation [15]. This problem is at least partially attributable to the pro-inflammatory and pro-oxidant host environments that prevail at the injured sites. Several proposals have been made to enhance the endogenous antioxidant capacities of transplanted MSCs with the aim of improving the efficacy of transplantation [16–18]. For example, several small-molecule antioxidants or reactive oxygen species (ROS) scavengers, such as edaravone and *N*-acetyl-L-cysteine (NAC), have been explored to enhance the stress resistance of MSCs [19, 20], but there is no definite evidence that these compounds can sustainably boost the resilience of MSCs due to a paucity of mechanistic details. Since the nuclear factor erythroid 2-related factor 2 (Nrf2) has a protective impact on stem cells in response to various environmental cues via the regulation of pluripotency factors, redox homeostasis, ageing, and cellular stress responses [21], activation of Nrf2 could be a plausible method for enhancing the efficacy of stem cell transplantation [22]. However, the underlying mechanisms and subsequent profiles of MSC-based secretory factors are poorly understood. In addition, the lack of information on the direct molecular targets of such paracrine actions also impedes advances in therapeutic inventions. While several mechanisms have been revealed in disease models in animals, research has shown that the key potential targets mediating MSC-related benefits require rigorous scrutiny using molecular and biochemical approaches [23, 24]. For instance, as a canonical inhibitor of Wnt signaling, Dickkopf1 (DKK1) was previously shown to be secreted by MSCs to ameliorate tissue injury and reduce liver fibrosis [25, 26].

With this in mind, we explored the antioxidant effects of Nrf2 and DDK1 in MSC. This study aimed to establish a transient plasmid-based pretreatment strategy to improve the stress resistance of MSCs and their paracrine effects on recipient liver failure cells, with the goal of providing a new perspective in the treatment of liver failure.

## Materials and methods

### MSC isolation, culture, and validation of surface markers

Commercially available human adipose-derived mesenchymal stromal cells (hADMSCs, #HUXMD-01001) were purchased from Cyagen Biosciences (Guangzhou, China) and handled according to the manufacturer’s instructions. This product was authenticated by the manufacturer and tested negative for mycoplasma, bacteria, endotoxins, and contamination. Flow cytometry was used to characterize human MSCs. Specific cell surface markers for hADMSCs, including CD105, CD73, and CD90 protein expressions, were assessed, while the absence of CD45, CD34, CD14 or CD11b, CD79a or CD19, and HLA-DR proteins confirmed the identity of the cells [27]. Therefore, these selected molecules were utilized for identification purposes. For validation, the following BD PharmingenTM monoclonal antibodies (mAbs) were used: phycoerythrin (PE) conjugated mouse antibodies with anti-human immunoreactivity for CD34 (#555822), CD44 (#555479), CD45 (#555483), and CD105 (#560839) (BD Biosciences, San Jose, CA). hADMSCs were separately incubated with the above mAbs or mouse IgG isotype control for 30 min at 4°C. Excess mAbs were removed by washing twice with phosphate buffer saline (PBS). Cells were resuspended in PBS (0.5 mL) to achieve a final density of 2 × 10^5^ cells prior to acquisition and analysed using a FACSCalibur flow cytometer (BD Biosciences). Surface marker expression of hADMSCs was assessed by flow cytometry after two passages. The results indicated high expression of CD44 and CD105 (all >94%) and very low expression of CD34 or CD45 (all <0.5%) (Supplementary Figure 1).

### Plasmid construction and transfection

Construction of the pIRES2–Nrf2–DKK1 plasmid, which was used to simultaneously overexpress Nrf2 and DKK1 in MSCs prior to their administration, was based on the pIRES2–EGFP vector (Clontech, Mountain View, CA; #6029–1). cDNA inserts from coding domain sequences (CDS) of the Nrf2 (GenBank accession: NM_006164) and DKK1 genes (GenBank accession: NM_012242) were synthesized by using polymerase chain reaction (PCR). The following gene-specific primers were used: Nrf2:5’- cagtGGATCCATGATGGACTTGGAGCTG-3’ and 5’- cagtGAGCTCCTAGTTTTTCTTAACATCTGGCTTC-3’; DKK1:5′- ATGATGGCTCTGGGCGCA-3′ and 5′- TTAGTGTCTCTGACAAGTGTGAAG-3′.’ The EGFP gene in the pIRES2–EGFP vector was then replaced by a DKK1 CDS sequence to generate a pIRES2–DKK1 vector. The Nrf2 gene fragment was inserted into the pIRES2–DKK1 vector at the SacI and BamHI restriction sites. Finally, overexpression (OE) plasmids containing the CDS of both human Nrf2 and DKK1 genes were obtained by PCR amplification and subcloning into empty pCDNA3.1 plasmid. A list of plasmid constructs is provided in Supplementary Table 1. Knock-down (KD) of endogenous Nrf2 (#NM-006164-07241504MN), DKK1 (#NM-012242-07241504MN), NAD (P) H quinone dehydrogenase 1 (NQO-1, #NM-000903-07241504MN), and heme oxygenase-1 (HO-1, #NM-002133-07241504MN) expression was achieved using the corresponding human MISSION short hairpin RNAs (shRNAs) purchased from Sigma-Aldrich (St Louis, MO). Transfection of plasmids or shRNAs into MSCs was conducted using a Lipofectamine 3000 system (#1687583; Invitrogen, Carlsbad, CA, USA). The efficiencies of the genetic OE and KD were verified according to the manufacturer’s instructions.

### Cell culture, reagents, and chemicals

All cell culture reagents and consumables were purchased from Gibco (Carlsbad, CA, USA) and Corning, Inc. (Corning, NY, USA). Hydrogen peroxide (H_2_O_2_; #H1009), NAC (#S0077) and methylthiazolyldiphenyl-tetrazolium bromide (MTT; #2128) were purchased from Sigma-Aldrich (St Louis, MO). The mitochondria-targeted antioxidant (MitoQ) was purchased from MedChemExpress (#HY-100116; Monmouth Junction, NJ, USA). Antibodies for the human proliferating cell nuclear antigen (PCNA; #ab18197), Bcl-2 (#ab196495), phosphorylated p38 mitogen-activated protein kinase (MAPK) at Thr180/Tyr182 (#ab4822), total p38 MAPK (#ab31828), phosphorylated p44/42 MAPK (ERK) at Thr202/Tyr204 (#ab214362), total ERK (#ab17942), dickkopf-1 (DKK1; #ab93017), alpha-1 antitrypsin (αAT; #ab166610), albumin (Alb; #ab106582), NRF2 (#ab92946), cyclin D1 (#ab16663), STAT1 (#ab239360), phosphorylated STAT1 at S727 (#ab109461), STAT3 (#ab68153), phosphorylated STAT3 at Y705 (#ab76315), heme oxygenase-1 (HO-1; ab189491), NAD(P)H quinone dehydrogenase 1 (NQO-1; #ab80588), and glycerol-3-phosphate dehydrogenase (GAPDH; #ab8245) antibodies were provided by Abcam (Cambridge, England). p38 MAPK inhibitor SB203580 (#S8307) and ERK inhibitor U0126 (#U120) were purchased from Sigma-Aldrich (St Louis, MO, USA). These reagents were added (20 μM) to the cell culture medium 2 h before the toxin (e.g. lipopolysaccharide/H_2_O_2_ or ethanol) treatment. Recombinant human tumor necrosis factor-alpha was purchased from PeproTech (Rocky Hill, NJ; #300-01A).

### AAV8-shRNA preparation

Adeno-associated virus type 8 (AAV8) was produced by transfecting AAV-293 cells with three plasmids: an AAV vector expressing shRNA targeting mouse cytoskeleton-associated protein 4 (CKAP4)/low-density lipoprotein receptor-related protein 6 (LRP6), an AAV helper plasmid (pAAV Helper), and an AAV Rep/Cap expression plasmid. At 72 h post-transfection, the cells were harvested and lysed following a freeze–thaw procedure. The viral particles were purified using an iodixanol step-gradient ultracentrifugation method. The iodixanol was subsequently diluted and AAV was concentrated using a 100-kDa molecular-weight cut-off ultrafiltration device. A genomic titer of 2.5–5.0 × 10^12^ infectious units per microliter was determined by using real-time quantitative PCR. To construct shRNAs, oligonucleotides containing sense and antisense sequences were joined by a hairpin loop followed by a poly (T) termination signal. The sequences targeting mouse CKAP4 (GenBank accession: NM_175451.1) and mouse LRP6 (GenBank accession: NM_008514.4) as used in the experiments were 5′-CCAAGTCTATCAATGACAACA-3′ and 5′-CGCACTACATTAGTTCCAAAT-3′,’ respectively. The sequence used to generate the mock control shRNA was TTCTCCGAACGTGTCACGT. The shRNAs were ligated into an AAV8 vector expressing the H1 promoter and EGFP.

### ACLF mouse model

Male 7-week-old wild-type (WT) C57BL/6J mice (∼21 g) were procured from the Guangdong Experimental Animal Center (Guangzhou, China). After randomization, 10 mice were assigned to each group. The ACLF model was established as previously described [28]. Briefly, mice were injected intraperitoneally (*i.p.*) with CCl_4_ (0.2 mL/kg twice a week) for 8 weeks, with subsequent injection of a double dose of CCl_4_ (0.4 mL/kg) followed by an *i.p*. injection of *Klebsiella pneumoniae* (*K.P.*) strain 43816 (ATCC, Manassas, VA). All experimental procedures were approved by the Ethical Committee of Shenzhen Third People’s Hospital (SZTPH: 2016–07). For *in vivo* assessment, mouse serum and liver tissue were collected on Day 3 after MSC transplantation; this was because our previous studies had shown that sampling on Day 3 (an experimentally optimized window for observation) would allow sufficiently informative evaluation of therapeutic effects from drugs or MSCs [17, 29]. For *in vivo* viral injection for CKAP4/LRP6 hepatic KD, mice were injected in the tail vein with 1 × 10^12^ genomic copies of AAV8 control or AAV-shRNA (five per group). The mice were maintained under a 12-h light/12-h dark cycle. After 14 days, the mice were fasted for 4 h at the end of the dark cycle and sacrificed to detect the downregulation of CKAP4 or LRP6 in different tissues.

### Cell viability

Changes in MSC viability after specific treatment(s) were measured using MTT assay. After treatment, the cells were washed three times with sterile PBS and then incubated with 5 mg/mL of MTT (Sigma-Aldrich; #M2128) for 4 h. The reaction products were subsequently dissolved in dimethyl sulfoxide (DMSO, Sigma-Aldrich; #D2650). The absorbance of the MTT was measured at 570 nm and pure DMSO was used as a blank control.

### Apoptotic percentage measurement

After treatments, Hoechst 33342 (5 μg/mL, Sigma-Aldrich; #B2261) and propidium iodide (5 μg/mL, Sigma-Aldrich; #P4170) were added simultaneously to each well to stain live MSCs. The cell population was divided into three groups: healthy cells showing a low level of blue fluorescence, apoptotic cells showing a higher level of blue fluorescence, and dead cells showing low blue and high red fluorescence. The stained cells were observed and quantified by two independent experimenters who were blinded to the group assignment. The results are expressed as apoptosis (%) = apoptotic cell number/total cell number × 100%.

### Caspase-3 activity assay

Activities of caspase-3 in cell lysates after treatment were measured using the Cell Meter Caspase 3 Activity Apoptosis Assay Kit (#22795; AAT Bio, Sunnyvale, CA, USA) according to the manufacturer’s instructions. The results were read at 520 nm using a microplate reader (Bio-Rad) and expressed as the fold change in caspase-3 activity relative to the control.

### Caspase-8 activity assay

Caspase-8 activity was detected using a colorimetric Caspase-8 activity assay kit (Novus Biologicals, Centennial, CO, USA; #NBP2-54817). Briefly, the cell lysate was incubated with Ile-Glu-Thr-Asp-p-nitroanilide (IETD-pDA) substrate at 37°C for 1 h according to the manufacturer’s instructions. Absorbance was read at 405 nm using a microplate reader (Bio-Rad).

### Measurement of oxidative stress in MSCs

CellROX^®^ oxidative stress reagent (Invitrogen, Carlsbad, CA; #C10444) is a novel fluorogenic probe for measuring oxidative stress in living cells. After the treatments, CellROX^®^ reagent was added at a final concentration of 5 µM to the MSCs, followed by incubation for 30 min at 37°C for fluorescence (green color) measurement using an inverted fluorescent microscope IX71 (Olympus microscope, Tokyo, Japan). Positive signals were quantified using ImageJ software (Version 1.52 NIH, Bethesda, MD, USA).

### Detection of mitochondrial superoxide by flow cytometry

After the treatments, MSCs mitochondrial superoxide was measured with 5 μM MitoSOX (Invitrogen; #M36008) for 15 min at 37°C. The cells were then washed with PBS, treated with trypsin, and resuspended in PBS containing 1% (v/v) heat-inactivated fetal bovine serum (FBS). Data were acquired using a FACSCalibur machine (BD Biosciences, San Jose, CA, USA) and analysed using CellQuest analytical software.

### Serum and liver tissue processing and analysis

After sacrificing the animals, their serums were collected by centrifugation from whole blood samples at 1,000 *g* for 10 min at 4°C and stored at –80°C. Serum alanine aminotransferase (ALT) and aspartate aminotransferase (AST) levels were measured using ALT (SGPT; #A524-150) and AST (SGOT; #A559-150) reagent sets (Teco Diagnostics, Anaheim, CA, USA) according to the manufacturer’s instructions. The liver tissue samples were fixed in 10% phosphate-buffered formalin, processed for histological analysis, and embedded in paraffin. Hepatic histology and fibrosis were visualized by staining with hematoxylin & eosin (H&E) or Sirius Red using a LEICA Qwin Image Analyser (Leica Microsystems Ltd, Milton Keynes, UK).

### Western blotting and enzyme-linked immunosorbent assay (ELISA) on key hepatic genes

Protein extraction/quantification from MSCs or murine liver tissues, as well as Western blotting assays, were conducted as previously described [30] and parallel blotting with GAPDH was used as an internal loading control. Activity changes in Nrf2 (#TFEH-NRF2) were evaluated using ELISA kits from RayBiotech (Norcross, GA). Human IL-1β (#ab214025), IL-6 (#ab178013), IL-10 (#ab185986), MCP-1 (#ab179886), RANTES (#ab174446), NQO-1 (#ab28947), and HO-1 (#ab133064) protein level changes were quantified using ELISA kits from Abcam. The number of cells was the same in both the control and stimulated groups.

### Assay on transplantation safety

To verify the long-term transplantation safety of MSCs in healthy and ACLF mice, we performed a 24-week tumorigenicity study as previously described elsewhere [30]. Healthy 7-week-old C57BL/6J male mice (with or without ACLF induction) received 1 × 10^7^ MRC-5 (negative control; ATCC, Manassas, VA; #CCL-171), 1 × 10^7^ ES-D3 (positive control; #CRL-11632), or 1 × 10^7^ hADMSCs (MSCs group, with or without pIRES-Nrf2-DKK1 plasmid pre-transfection) (12 mice per group for healthy and 18 mice for ACLF group). After 24 weeks, or when the mice exhibited severe symptoms of dyspnea and minimal activity, they were sacrificed to assess the extent of tumor formation.

### Statistical analysis

Data from each group are presented as mean ± standard deviation. Unless otherwise stated, statistical comparisons between groups were performed using the Kruskal–Wallis test, followed by Dunn’s post hoc test to determine differences in all groups. *P *<* *0.05 was considered statistically significant (GraphPad Prism 5.0; San Diego, CA).

## Results

### Nrf2 and DKK1 both promote the anti-stress capacity of MSCs

**Figure 1.**
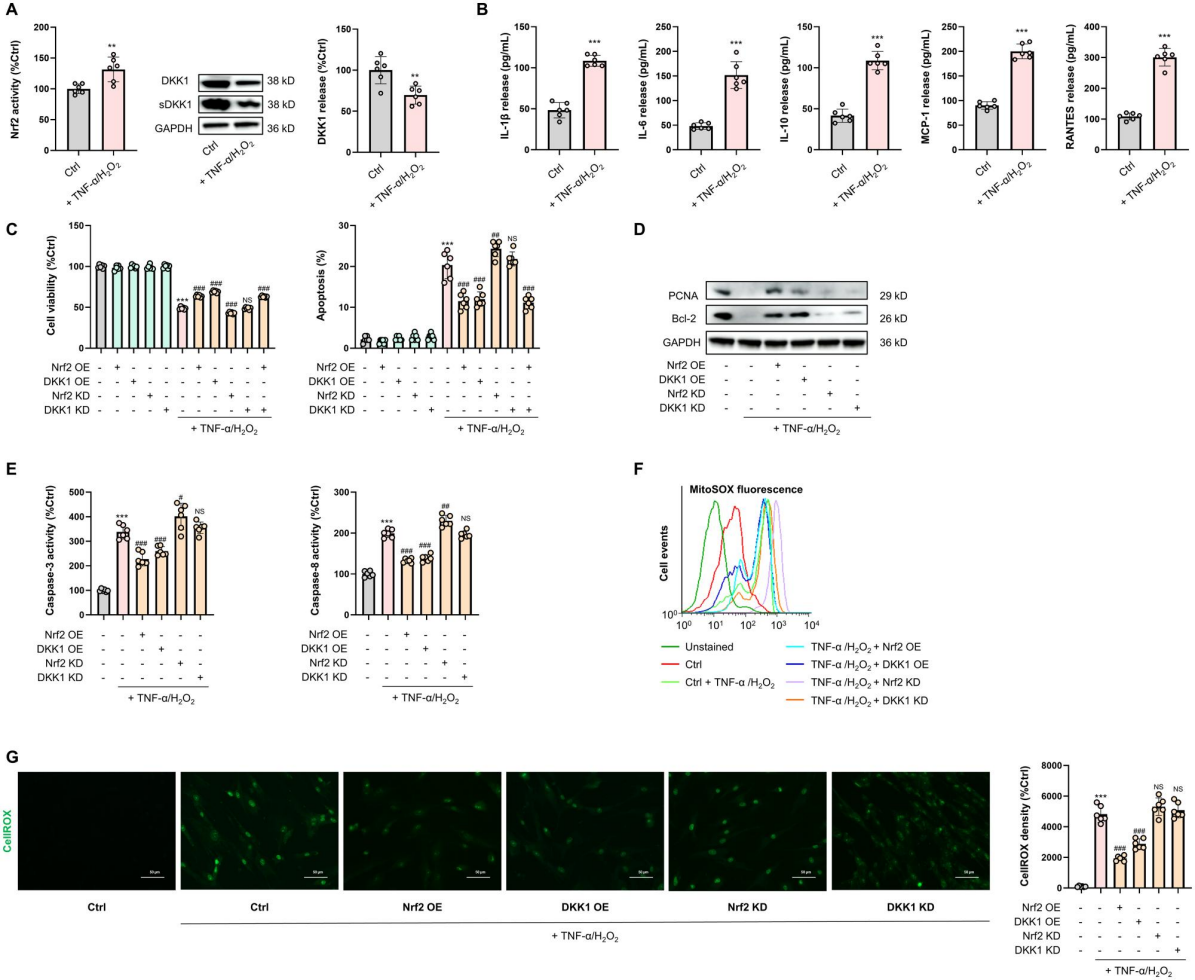
Co-stimulation of Nrf2 and DKK1 signaling boosts the anti-stress capacity of human MSCs. (A) Left: Changes in Nrf2 activity in MSCs with or without TNF-α/H_2_O_2_ co-treatment. Right: Representative immunoblot results for DKK1 and secreted DKK1 (sDKK1) in MSCs with a similar test design as in (A) (*n *=* *6). (B) ELISA results of released IL-1β, IL-6, MCP-1, RANTES, and IL-10 protein from MSCs treated with TNF-α/H_2_O_2_ (*n *=* *6). (C) Changes in cell viability and apoptotic ratios of MSCs following challenge with TNF-α/H_2_O_2_ in the presence or absence of Nrf2/DKK1 manipulations (*n *=* *6). (D) Representative immunoblot results for PCNA and Bcl-2 from MSCs following the aforementioned treatments. (E) Changes in cellular caspase-3/8 activity of MSCs with the aforementioned treatments (*n *=* *6). (F) Changes in mitochondrial superoxide levels in MSCs measured by MitoSOX in flow cytometry following the aforementioned treatments. (G) Fluorescence micrographs for the detection of cellular ROS by CellROX Green and corresponding quantified fluorescence intensities in MSCs treated with TNF-α/H_2_O_2_ with the aforementioned treatments (*n *=* *6). Scale bar = 50 μm. hADMSCs = human adipose-derived mesenchymal stromal cells. Values are expressed as mean ± SD. *, **, and *** indicate *P *<* *0.05, 0.01, and 0.001 against an untreated MSC group (or between indicated groups), respectively; ^#^, ^##^, and ^###^ indicate *P *<* *0.05, 0.01, and 0.001 against TNF-α/H_2_O_2_-treated control group, respectively.

Since the transplantation of MSCs preconditioned with minocycline or doxycycline protects against ischemic injury in murine models via Nrf2 activation [22, 31], we first examined whether treatment with tumor necrosis factor-alpha plus hydrogen peroxide (TNF-α/H_2_O_2_)—a well-characterized ACLF-like cell model [32–34]—altered the basal Nrf2 activity and the release of key soluble cytokines/chemokines. A 24-h treatment with TNF-α/H_2_O_2_ significantly promoted the activity of Nrf2 in MSCs, whereas it decreased the translational and secreted levels of Wnt canonical pathway inhibitor DKK1 (Figure 1A). TNF-α/H_2_O_2_ treatment also enhanced the secretion of pro-inflammatory cytokines/chemokines [IL-1β, IL-6, MCP-1, and regulated upon activation, normal T cell expressed and secreted (RANTES)] and anti-inflammatory cytokine (IL-10) from MSCs, indicating an ACLF-comparable inflammatory environment in the cell culture system (Figure 1B). Moreover, a dose-dependent increase in *DKK1* activity was observed when *Nrf2* was knocked down using siRNA (Supplementary Figure 2A). Then the basal expression of *Nrf2* and *DKK1* was increased or decreased by transfection with expression plasmid or shRNA, respectively, at the 48-h time point before the treatment with TNF-α/H_2_O_2_ (Supplementary Figure 2B). Silencing of *Nrf2* reduced cell viability in TNF-α/H_2_O_2_-challenged MSCs, whereas overexpression of Nrf2 or DKK1 improved cell viability (*P *<* *0.001). Inhibition of DKK1 in the absence of Nrf2 overexpression does not influence the cell viability after TNF-α/H_2_O_2_ stimulation (Figure 1C). We observed consistent changes in the MSC apoptotic ratio after manipulation of the basal expression of Nrf2 and DKK1 (Figure 1C). In addition, changes in the protein expression of the cell cycle regulator, PCNA, and the apoptotic negative regulator, Bcl-2, reflected the influence of Nrf2/DKK1 signaling on MSC viability and apoptosis, respectively (Figure 1D). This result was further strengthened by the activity changes in the caspase-3/8 of MSCs (Figure 1E). Since endogenous production of excessive cellular and mitochondrial ROS in MSCs can arise as a direct consequence of extrinsically imposed TNF-α/H_2_O_2_ toxicity [32], we proceeded to verify the effects of Nrf2/DKK1 modulation on oxidant-induced cellular and mitochondrial dysfunction by the detection of oxidative events using CellROX (cellular oxidative stress probe) and MitoSOX (mitochondrial superoxide probe), respectively. As anticipated, exposure to TNF-α/H_2_O_2_ elevated the endogenous production of the cellular/mitochondrial ROS of MSCs, which was alleviated by Nrf2 or DKK1 overexpression. However, the KD of Nrf2 or DKK1 slightly exacerbated cellular/mitochondrial ROS production in MSCs (Figure 1F and G). Collectively, Nrf2 and DKK1 both promote the anti-stress capacity of MSCs.

### Mobilization of Nrf2/DKK1 signaling in MSCs inhibits ROS production through the antioxidant effects of NQO-1 and HO-1

**Figure 2.**
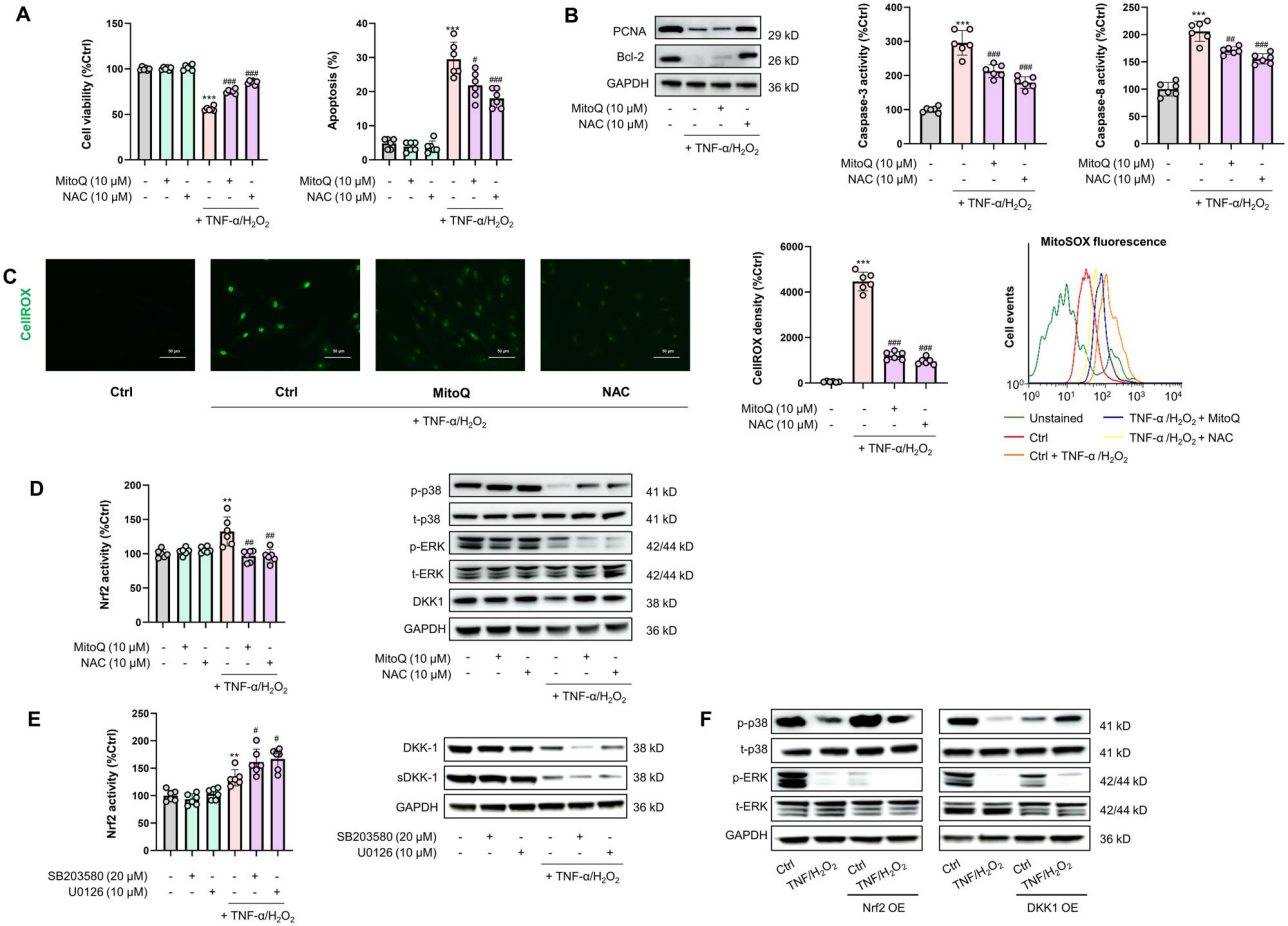
Enhanced MSC resilience through co-stimulation of MAPK/Nrf2 and DKK1 signaling is attained by reducing ROS generation. (A) Cell viability and apoptotic ratio changes in MSCs treated with TNF-α/H_2_O_2_ in the presence or absence of MitoQ or NAC (*n *=* *6). (B) Representative immunoblotting results for PCNA and Bcl-2 in MSCs (left) and changes in cellular caspase-3/8 activity of MSCs with a similar test design as in (A) (*n *=* *6). (C) (Left) Fluorescence micrographs for the detection of cellular ROS by CellROX Green and corresponding quantified fluorescence intensities in MSCs following antioxidant intervention (*n *=* *6) (Scale bar = 50 μm). (Right) Changes in mitochondrial superoxide levels in MSCs following antioxidant intervention, as measured by MitoSOX in flow cytometry (*n *=* *6). (D) Assays on transcriptional activities of Nrf2 (left) and representative immunoblot results (right) for phosphorylated p38 MAPK (p-p38), total p38 MAPK (t-p38), p-ERK, t-ERK, and DKK1 in MSCs following antioxidant intervention (*n *=* *6). (E) (Left) Changes in transcriptional activities of Nrf2 in MSCs following TNF-α/H_2_O_2_ challenge in the presence or absence of MAPK/ERK inhibitors (*n *=* *6). (Right) Representative immunoblot results for DKK1 and secreted DKK1 (sDKK1) in MSCs following the aforementioned treatments. (F) Representative immunoblot results for p-p38, t-p38, p-ERK, and t-ERK of MSCs following TNF-α/H_2_O_2_ challenge, with or without Nrf2/DKK1 overexpression (OE). Data are expressed as mean ± SD. ** and *** indicate *P *<* *0.01 and 0.001 against an untreated MSC group, respectively; ^#^, ^##^, and ^###^ indicate *P *<* *0.05, 0.01, and 0.001 against a TNF-α/H_2_O_2_ group, respectively. SB, SB203580, the inhibitor of p38 MAPK; U0, U0126, the inhibitor of ERK.

**Figure 3.**
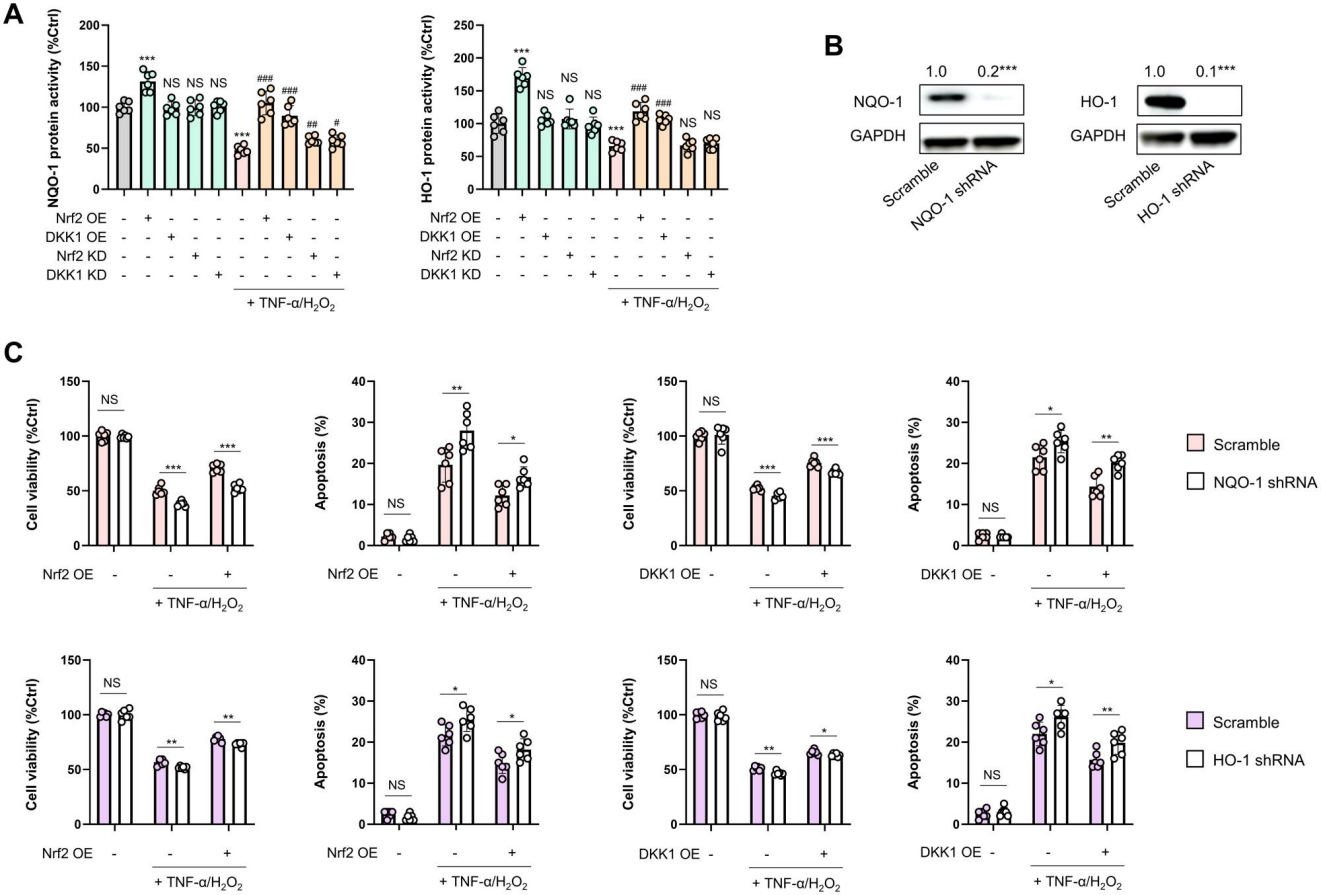
Regulatory loop between Nrf2/DKK1 and NQO-1/HO-1 in MSCs. (A) Changes in NQO-1/HO-1 protein expression of MSCs following TNF-α/H_2_O_2_ challenge in the absence or presence of Nrf2/Dkk1 manipulations (*n *=* *6). (B) Representative immunoblot results for MSCs when endogenous NQO-1/HO-1 was inhibited specifically by shRNAs. (C) Changes in cell viability or apoptotic ratios of MSCs following TNF-α/H_2_O_2_ challenge in the presence or absence of NQO-1/HO-1 inhibition or Nrf2/DKK1 overexpression (OE) (*n *=* *6). Data are expressed as mean ± SD. For (A) and (B), *** indicates *P *<* *0.001 against an untreated MSC group; ^#^, ^##^, and ^###^ indicate *P *<* *0.05, 0.01, and 0.001 against a corresponding TNF-α/H_2_O_2_ group, respectively. For (C), *, **, and *** represent *P *<* *0.05, 0.01, and 0.001 between indicated groups, respectively.

To substantiate mechanistically how Nrf2/DKK1 signaling contributes to MSC resilience against TNF-α/H_2_O_2_-induced stress, we investigated the interrelations between this pathway and that for ROS production in human MSCs by using MitoQ (mitochondrial ROS scavenger) and NAC (total cellular antioxidant) in the presence of TNF-α/H_2_O_2_. Consistently with our assumptions, co-treatment with MitoQ or NAC ameliorated TNF-α/H_2_O_2_-induced cell damage in several aspects, including an increase in cell viability/PCNA expression and a reduction in apoptosis and cellular/mitochondrial ROS production (*P *<* *0.05) (Figure 2A–C). Nrf2 activity in MSCs was increased by TNF-α/H_2_O_2_ exposure but was rebalanced by co-treatment with MitoQ or NAC. Similarly, the TNF-α/H_2_O_2_ challenge reduced p38 MAPK phosphorylation and DKK1 protein expression, which were substantially restored by MitoQ/NAC co-treatment (Figure 2D). Notably, the decreased ERK phosphorylation seen during TNF-α/H_2_O_2_ exposure was further suppressed by MitoQ/NAC (Figure 2D). Although previous studies have postulated cross-regulation between MAPKs and DKK1 in cancer and T cells [35–37], it remains largely unknown whether crosstalk exists between the MAPK and Nrf2/DKK1 pathways in human MSCs. We found that the provoked Nrf2 activity was further enhanced by SB203580 (p38 MAPK inhibitor) or U0126 (MEK1/2 inhibitor) *in vitro* (*P *<* *0.05). Changes in cellular and secreted DKK1 levels were opposite to those of Nrf2, further confirming a negative regulatory loop between Nrf2 and DKK1 (Figure 2E). When Nrf2 was overexpressed by plasmid transfection, both the basal and TNF-α/H_2_O_2_-suppressed levels of phosphorylated p38 MAPK were elevated. In contrast, Nrf2 overexpression strongly suppressed ERK phosphorylation under basal and TNF-α/H_2_O_2_-treated conditions (Figure 2F). Overexpression of DKK1 in MSCs increased phosphorylated p38 levels but decreased phosphorylated ERK levels when exposed to TNF-α/H_2_O_2_ (Figure 2F). Moreover, TNF-α/H_2_O_2_ exposure diminished the cellular levels of NAD(P)H quinone dehydrogenase 1 (NQO-1) and heme oxygenase-1 (HO-1) (Figure 3A), which are Nrf2-regulated antioxidant enzymes important to stem cell homeostasis [38]. Nrf2 overexpression significantly improved both the basal and TNF-α/H_2_O_2_-repressed levels of NQO-1 and HO-1 (Figure 3A). When the endogenous expression of NQO-1 or HO-1 in either type of human MSCs was silenced by specific shRNAs, the ameliorative effects of Nrf2 or DKK1 overexpression on cell injury were curtailed (*P *<* *0.05) (Figure 3B and C), suggesting a dependence on NQO-1 and HO-1 as distal mediators in the MSC anti-stress response. Collectively, the mobilization of Nrf2/DKK1 signaling in MSCs inhibits cellular/mitochondrial ROS production to a certain extent through the antioxidant effects of NQO-1 and HO-1.

### Co-expression of Nrf2 and DKK1 boosts anti-stress capacity of MSCs

**Figure 4.**
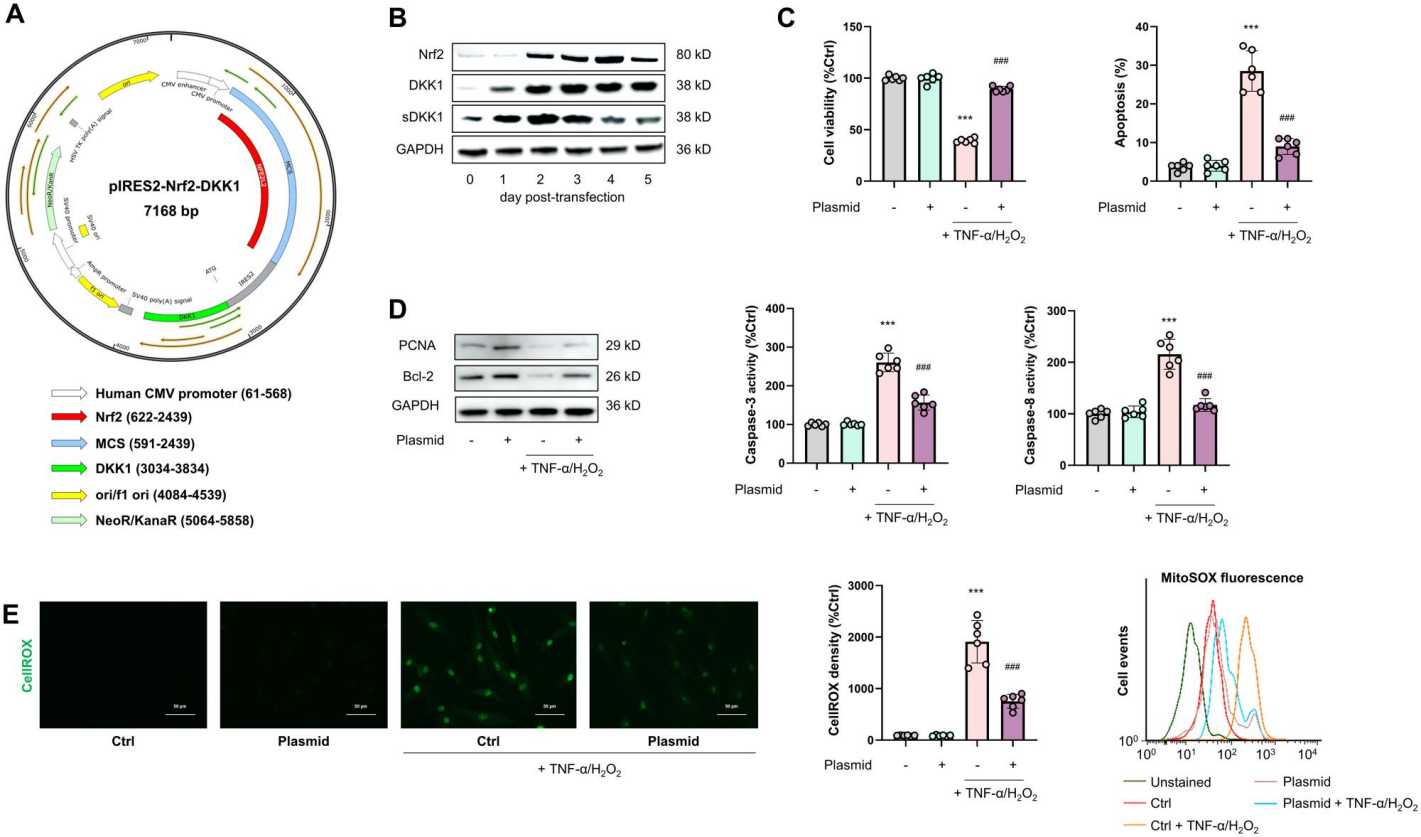
Preconditioning by co-overexpression of Nrf2 and DKK1 enhances MSC resistance to exogenous stress. (A) Plasmid map for the constructed pIRES2–Nrf2–DKK1. (B) Representative immunoblot results for time-lapse study (Day 0–Day 5) on Nrf2, DKK1, and secreted DKK1 (sDKK1) expression in MSCs following transfection of the pIRES2–Nrf2–DKK1 plasmid. (C) Changes in cell viability (Left) and apoptotic ratios (Right) of MSCs following TNF-α/H_2_O_2_ challenge with or without transfection of the pIRES2–Nrf2–DKK1 plasmid (*n *=* *6). (D) (Left) Representative immunoblotting results for PCNA and Bcl-2 in MSCs and (Right) changes in cellular caspase-3/8 activity of MSCs following TNF-α/H_2_O_2_ challenge with or without transfection of the pIRES2–Nrf2–DKK1 plasmid *(n *=* *6). (E) (Left) Detection of cellular ROS in MSCs by CellROX Green and corresponding quantified fluorescence intensities in MSCs following TNF-α/H_2_O_2_ challenge with or without transfection of the pIRES2–Nrf2–DKK1 plasmid (*n *=* *6) (Scale bar = 50 μM). (Right) Changes in mitochondrial superoxide levels in MSCs, following TNF-α/H_2_O_2_ challenge with or without transfection of the pIRES2–Nrf2–DKK1 plasmid (*n *=* *6). Data are expressed as mean ± SD. *** indicates *P *<* *0.001 against an untreated MSC group; ^###^ indicates *P *<* *0.001 against an TNF-α/H_2_O_2_ group.

To sum up, we found that the overexpression of Nrf2 and DKK1 could alleviate inflammation- and oxidative stress-induced cell injury in MSCs. To maximize the alleviative effects, we attempted to co-overexpress Nrf2 and DKK1 by constructing an pIRES2–Nrf2–DKK1 expression plasmid (Figure 4A), which was transfected into MSCs for 5 days (approximately the same duration for which MSCs needed to endure the harsh effects of local stress following transplantation) to verify its protein-inductive potential. Following transfection, the protein expression of Nrf2, total DKK1, and secreted DKK1 in human MSCs began to increase on Day 2 and peaked on Day 3, while secreted DKK1 (sDKK1) returned to near-basal levels on Day 5 post-transfection (Figure 4B). Importantly, transient transfection of this plasmid did not alter MSC viability or differentiation potential (Supplementary Figure 3). Based on this observation, we subjected MSCs to a TNF-α/H_2_O_2_ challenge on Day 3 post-transfection for another 24 h to evaluate the anti-stress capacity of MSCs gained from plasmid transfection. As anticipated, pIRES2–Nrf2–DKK1 plasmid transfection significantly restored cell viability, reduced apoptosis, and reduced cellular/mitochondrial ROS production in MSCs without altering their basal status (*P *<* *0.001) (Figure 4C–E). Notably, empty vector transfection did not affect the MSC viability, apoptosis, or ROS production (Supplementary Figure 4). Collectively, the coordinated stimulation of Nrf2/DKK1 signaling contributes to the enhanced anti-stress capacity of MSCs.

### Co-expression of Nrf2 and DKK1 improves MSC transplantation efficacy in ACLF mice

**Figure 5.**
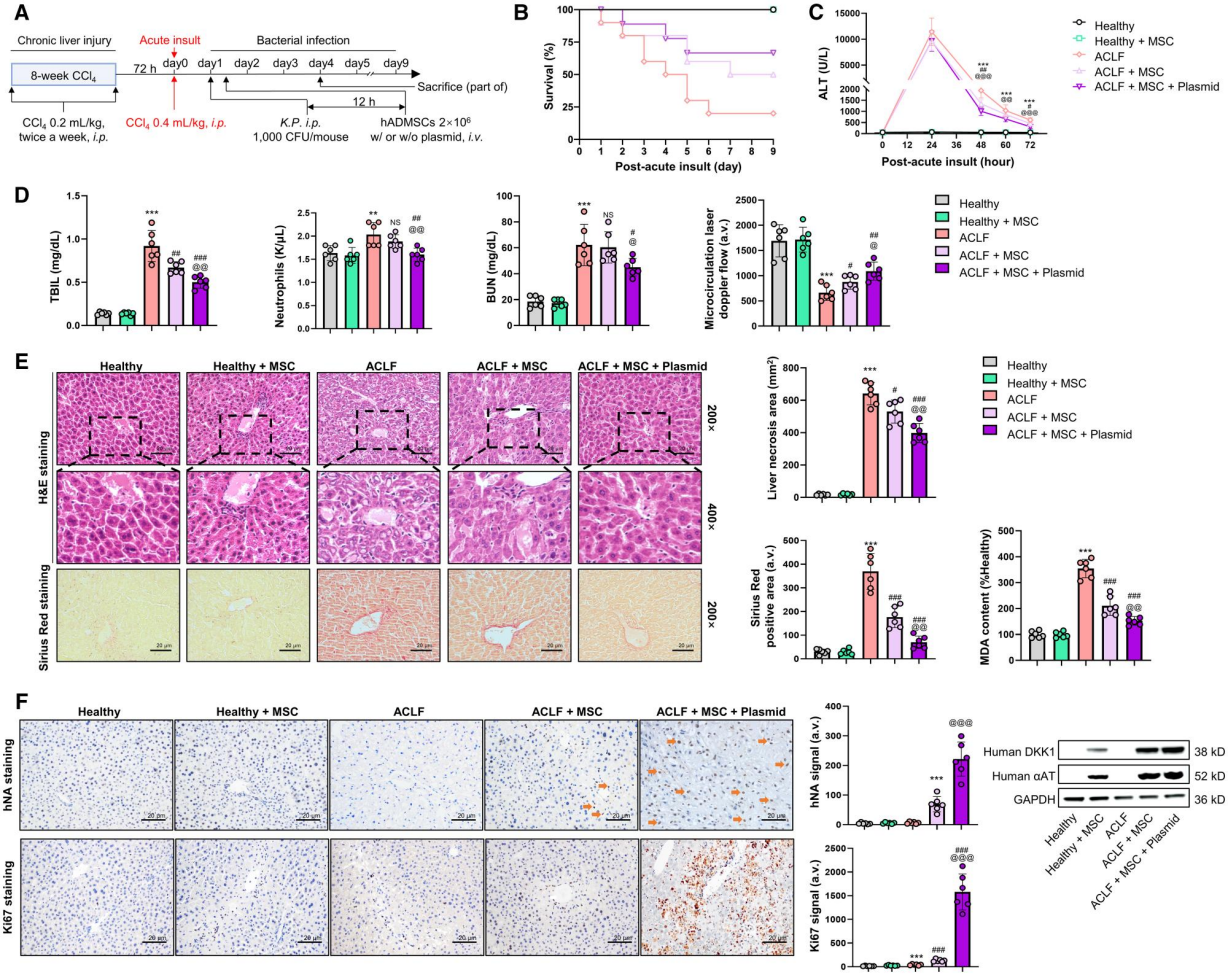
Preconditioning with a pIRES2–Nrf2–DKK1 plasmid improves transplantation efficacy of MSCs in a murine model of acute-on-chronic liver failure. (A) Schematic timeline of the acute-on-chronic liver failure (ACLF) mice model establishment with carbon tetrachloride (CCl_4_) and *Klebsiella pneumoniae* (*K.P*.) injection with or without MSC of Nrf2/DKK1 co-stimulation. (B) Survival counts of ACLF mice with or without injection of human MSCs (naive or pre-transfected with pIRES2–Nrf2–DKK1) for 9 days (*n *=* *10 per group). (C) Changes in serum ALT levels in mice as depicted in (A) (72 h post-*K.P*. administration; *n *=* *8). (D) Changes in liver total bilirubin (TBIL), circulating neutrophils, blood urea nitrogen (BUN), and renal microvascular flow in mice as depicted in (A) (72 h post-*K.P*. administration; *n *=* *6). (E) Representative images of H&E and Sirius Red staining of the mice liver, and corresponding quantification of liver necrosis areas and liver fibrosis areas, and changes in liver malondialdehyde (MDA) contents in mice as depicted in (A) (72 h post-*K.P*. administration; *n *=* *6). (F) Representative immunohistochemical results for human nuclear antigen (hNA) and Ki67 staining and representative immunoblot results for human DKK1/αΑT in the liver from mice as depicted in (A) (72 h post-*K.P*. administration; *n *=* *6). Scale bar = 50 μm Arrows indicate typical immunohistochemistry (IHC) signals. Data are expressed as mean ± SD. ** and *** indicate *P *<* *0.01 and 0.001 against a healthy group, respectively; ^#^, ^##^, and ^###^ indicate *P *<* *0.05, 0.01, and 0.001 against an ACLF group, respectively; ^@^, ^@@^, and ^@@@^ indicate *P *<* *0.05, 0.01, and 0.001 against an ACLF-challenged plasmid-naive MSC group, respectively.

Since this ACLF model has been reported to induce clear liver fibrosis, we also investigated the potential amelioration of fibrosis after MSC transplantation. Using an ACLF mouse model combining chronic liver injury, acute hepatic insult, and bacterial infection that phenocopies some of the key clinical features of ACLF patients [28], we tested the MSC-protective effects of Nrf2/DKK1 co-stimulation (Figure 5A). Empirically, the probability of death in ACLF mice (during a 9-day time window) was mitigated by the transplantation of human MSCs. In particular, preconditioning MSCs with the pIRES2–Nrf2–DKK1 plasmid before transplantation enhanced these protective effects. No further mortality was observed in any group during the extended 9-day observation period (Figure 5B). High levels of serum ALT and total bilirubin (TBIL), elevation of circulating neutrophils and blood urea nitrogen (BUN), and reduction of renal microvascular flow were all significantly alleviated by MSC transplantation and were further strengthened by pIRES2–Nrf2–DKK1 plasmid preconditioning (Figure 5C and D). ACLF-induced severe pathological liver damage, including necrosis, inflammation, fibrosis, and lipid peroxidation, was also ameliorated by MSCs and plasmid preconditioning (*P *<* *0.001) (Figure 5E). Successfully homed human MSCs in the damaged mice liver were labeled with human nuclear antigen (hNA) staining, DKK1 protein, and alpha-1 antitrypsin (αAT) protein, showing that transfection with the pIRES2–Nrf2–DKK1 plasmid significantly improved the homing efficacy of MSCs, which was closely associated with accelerated liver regeneration, as observed by hepatic Ki67 staining (Figure 5F). Therefore, the simultaneous expression of Nrf2 and DKK1 enhances the effectiveness of MSC transplantation in ACLF mice.

### MSCs preconditioned with pIRES2–Nrf2–DKK1 resolve ACLF injury by enhancing the pro-regenerative IL-6/STAT3 pathway and attenuating the anti-regenerative IFN-γ/STAT1 pathway

**Figure 6.**
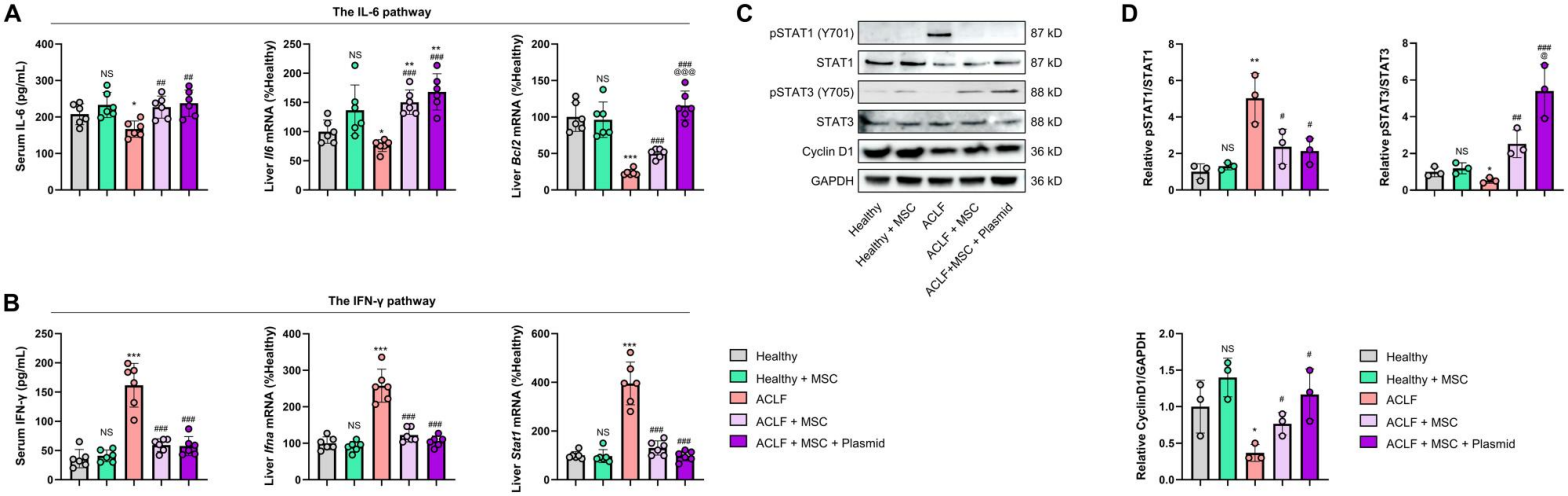
MSCs preconditioned with pIRES2–Nrf2–DKK1 resolve ACLF injury by enhancing the pro-regenerative IL-6/STAT3 pathway but attenuating the anti-regenerative IFN-γ/STAT1 pathway. (A) and (B) Serum IL-6 and IFN-γ, and relative mRNA expressions of *Il-6* and *Ifng*, and their downstream target genes (*Bcl2* and *Stat1*, respectively) in the ACLF or control mice after the transplantation with MSCs, with or without pIRES2–Nrf2–DKK1 plasmid preconditioning (*n *=* *6). (C) Liver extracts were subjected to Western blot analysis of phosphorylated STAT1 (pSTAT1), STAT1, pSTAT3, STAT3, and Cyclin D1 in mice as depicted in (A). (D) Relative quantification of STAT1 and STAT3 (the phosphorylated level was divided by the total protein level) and Cyclin D1 in mice as depicted in (A) (*n *=* *3*).* Data are expressed as mean ± SD. *, **, and *** indicate *P *<* *0.05, 0.01, and 0.001 against a healthy group, respectively; ^#^, ^##^, and ^###^ indicate *P *<* *0.05, 0.01, and 0.001 against an ACLF group, respectively; ^@^ and ^@@@^ indicate *P *<* *0.05 and 0.001 against an ACLF-challenged plasmid-naive MSC group, respectively.

To investigate the mechanisms for MSC transplantation-induced liver regeneration and fibrosis resolution, we measured serum cytokines and found that, in ACLF mice, the IL-6 protein level was inhibited (*P *<* *0.05) while the IFN-γ protein level was provoked (*P *<* *0.001) when compared with those of control mice. In the liver, the mRNA level changes in *Il6* and *Bcl2* corresponded with that of serum IL-6 protein, while *Ifna* mRNA level changes corresponded with that of serum IFN-γ protein (Figure 6A and B). In the liver, Western blot analyses revealed that ACLF mice had enhanced phosphorylated levels of STAT1 when compared with those of control mice, which were suppressed after transplantation with MSCs, with or without pIRES2–Nrf2–DKK1 plasmid preconditioning. In contrast, transplantation with MSCs enhanced the phosphorylated level of STAT3, which was further elevated by the pIRES2–Nrf2–DKK1 plasmid preconditioning (Figure 6C and D). In summary, following the model induction, there was no significant alteration in total STAT1 protein (although some changes in transcription levels were observed), yet an increase in phosphorylated STAT1 (pSTAT1) was noted. This indicated the activation of STAT1 activity in ACLF, with stem cell transplantation showing the potential to mitigate this pathological change. In contrast to STAT activation, cyclin D1 expression was lower in ACLF mice (*P *<* *0.05) than in control mice (Figure 6C and D). In conclusion, preconditioning MSCs with pIRES2–Nrf2–DKK1 effectively ameliorates ACLF injury by promoting the regenerative IL-6/STAT3 pathway and inhibiting the anti-regenerative IFN-γ/STAT1 pathway, providing insights into the mechanisms behind liver regeneration and fibrosis resolution in ACLF.

### Hepatocyte membrane receptor CKAP4 but not LRP6 mediates MSC-based recipient liver repair

**Figure 7.**
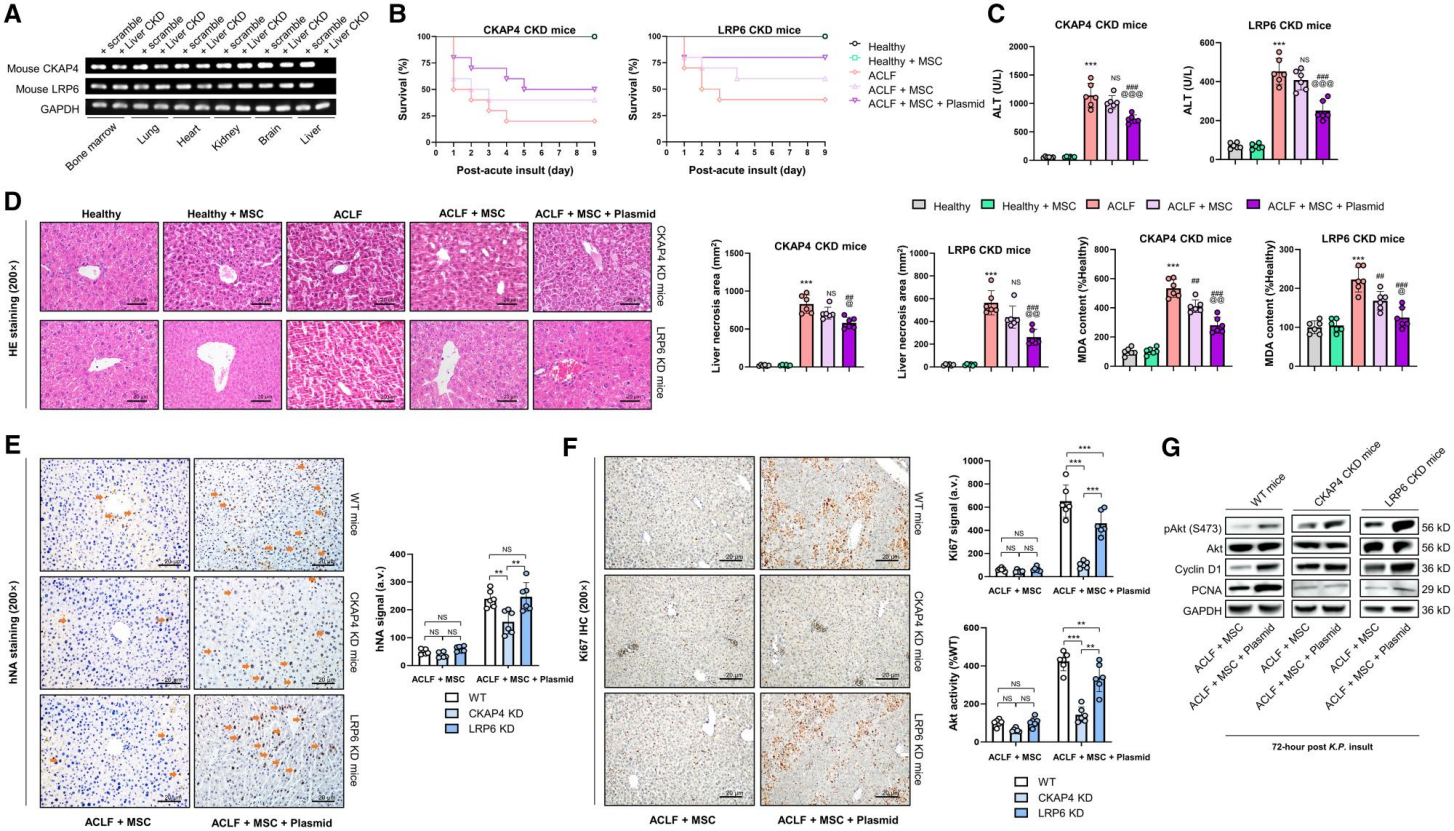
The DKK1 receptor CKAP4, but not LRP6, in host hepatocytes is a paracrine target of MSC-based therapy. (A) Representative genotyping results for hepatic-specific knock-down of CKAP4 and LRP6 by AAV8-mediated shRNA in mice (in the tissue extracts of bone marrow, lung, heart, kidneys, brain, and liver). (B) Survival counts of mice with ACLF challenge with or without injection of human MSCs (naive or pre-transfected with pIRES2–Nrf2–DKK1) for 9 days, following hepatic knock-down (CKD) of CKAP4 or LRP6 (*n *=* *10 per group). (C) Changes in serum ALT levels in mice as depicted in (B) (72 h post-*K.P*. administration; *n *=* *6). (D) Representative images for liver H&E staining and corresponding quantification of liver necrosis areas and liver fibrosis areas, and changes in liver MDA contents in mice as depicted in (B) (dashed lines indicate typical necrotic areas in the liver. 72 h post-*K.P*. administration; *n *=* *6). (E) Representative images for liver human nuclear antigen (hNA) immunohistochemical staining and corresponding quantification of hNA density in mice as depicted in (B) (72 h post-*K.P*. administration; *n *=* *6). Arrows indicate typical IHC signals. (F) Representative images for liver Ki67 immunohistochemical staining and corresponding quantification of Ki67 density and enzyme-immuno assay measurements of hepatic Akt activity in mice as depicted in (B) (*n *=* *6). (G) Representative immunoblot results for liver phosphorylated Akt (p-Akt), Akt, cyclin D1, and PCNA in mice with or without knock-down of hepatic CKAP4 or LRP6. Scale bar = 50 μm. Data are expressed as mean ± SD. *** indicates *P *<* *0.001 against a healthy group; ^##^ and ^###^ indicate *P *<* *0.01 and 0.001 against an ACLF group, respectively; ^@^, ^@@^ and ^@@@^ indicate *P *<* *0.05, 0.01, and 0.001 against an ACLF-challenged plasmid-naive MSC group, respectively. For (E) and (F), *, **, and *** represent *P *<* *0.05, 0.01, and 0.001 between indicated groups.

We speculated that DKK1 receptors on hepatocyte cell membranes transduced DKK1 signaling from transplanted human MSCs to promote repair processes in murine recipient hepatocytes. Thus, using AAV8-ligated shRNAs, we knocked down the expression of two well-documented hepatocyte DKK1 receptors: cytoskeleton-associated protein 4 (CKAP4) and low-density lipoprotein receptor-related protein 6 (LRP6) [39], specifically in the liver (Figure 7A). Compared with their WT littermates (Figure 5B), LRP6 conditional knock-down (CKD) mice showed a similar death rate, whereas CKAP4 CKD mice had a higher death rate upon ACLF challenge (Figure 7B). Changes in serum ALT levels, liver histology, and hepatic injury markers were consistent across the treatment groups (Figure 7C and D). Notably, the hepatic KD of CKAP4 affected hepatic hNA signaling, suggesting that the homing effect of MSC transplantation under pIRES2–Nrf2–DKK1 preconditioning was dependent on hepatocyte CKAP4 levels (Figure 7E). In contrast, hepatic staining of Ki67 and protein expression analysis of phosphorylated protein kinase B (p-Akt), cyclin D1, and PCNA revealed that hepatic KD of CKAP4, when compared with LRP6 KD or WT mice, resulted in significantly lower levels of liver regeneration, suggesting that MSC-based intervention for recipient liver cell proliferation was CKAP4-dependent (Figure 7F and G). In conclusion, our study shows that CKAP4, not LRP6, is essential for mediating MSC-induced liver repair during ACLF, highlighting the key role of CKAP4 in hepatic regeneration and signaling pathways.

### Long-term transplantation with plasmid-transfect MSCs is safe in murine models

**Figure 8.**
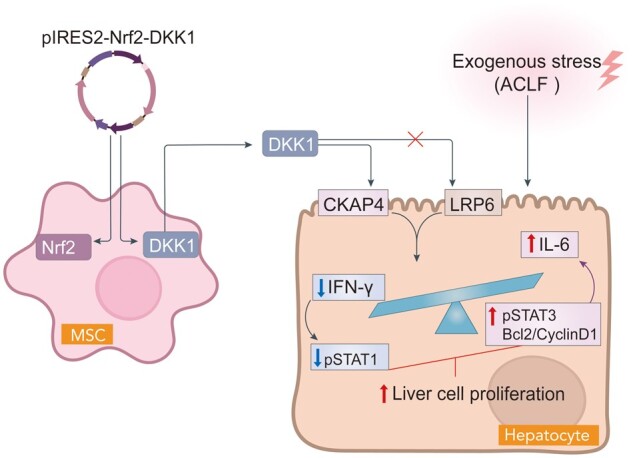
Co-stimulation of Nrf2/DKK1 signaling effectively and safely improves the efficacy of human MSC-based therapies in mouse ACLF models through CKAP4-dependent paracrine mechanisms

The potential risks of tumorigenicity are important safety issues in the development of MSC-based therapies, particularly for viral or plasmid-manipulated MSCs [40]. In our ACLF model, no mice developed tumors during the long-term observation period (24 weeks). In contrast, all animals in the positive control groups of the healthy and ACLF models showed severe symptoms of dyspnea and minimal activity 5–6 weeks after ES-D3 cell injection. Gross morphology analysis suggested that all mice developed tumors in the lungs (Supplementary Table 2). To assess the long-term viability of donor MSCs, human albumin was measured in the mouse serum at 12 and 24 weeks post-transplantation. Human albumin levels were found to be approximately two times higher in the serum of ACLF mice transplanted with Nrf2/DKK1 preconditioned MSCs than in mice with plasmid-naive MSCs at 12 weeks post-injection (*P *<* *0.05). In the 24th week, the differences in human albumin levels between mice transplanted with Nrf2/DKK1-preconditioned MSCs and those with plasmid-naive MSCs became less pronounced than those in the 12th week (Supplementary Figure 5). In conclusion, co-stimulation with Nrf2/DKK1 signaling effectively and safely improved the efficacy of human MSC-based therapies in mouse ACLF models through CKAP4-dependent paracrine mechanisms (Figure 8).

## Discussion

Accumulating evidence suggests that enhancement of the anti-stress capacity of transplanted stem cells favorably influences therapeutic outcomes in a variety of diseases, although the essential signaling pathways that regulate the mechanisms remain incompletely understood [41]. Previous studies have shown that Nrf2 and its upstream MAPK pathways are involved in antioxidant-promoted stem cell resistance against exogenous stress; however, their exact roles in reparative processes in liver injury require further elucidation [16, 17]. Therefore, we investigated the regulatory roles of p38 and ERK in the injury of MSCs. Inhibition of p38 MAPK worsened, while ERK inhibition alleviated TNF-α/H_2_O_2_-induced cell injury. Because Nrf2 is critical for protecting cells from harsh *in vivo* environments (e.g. inflammation, oxidative stress, and cell death signals) and DKK1 secretions mediated hepatocyte regeneration through paracrine mechanisms [25, 26], we decided to force their expression simultaneously. *In vitro* and *in vivo* studies have shown that MSCs transfected with plasmids concurrently overexpressing Nrf2 and DKK1 are more resistant to external stimuli than those without any treatment. Further analysis showed that Nrf2/DKK1 overexpression attenuated cellular/mitochondrial ROS production and restored antioxidant reserves. In addition, the augmented expression of Nrf2 and DKK1 increased the levels of phosphorylated p38 MAPK and reduced the levels of phosphorylated ERK, forming a positive feedback loop that sustained anti-stress regulation within preconditioned MSCs. These findings are consistent with those of several previous studies supporting direct crosstalk between MAPK and Nrf2/DKK1 in other cell types [36, 42–44].

The selective overexpression of key proteins to boost the anti-stress capacity of MSCs prior to transplantation is a theoretically sound strategy for improving therapeutic efficacy. For example, forced myocardin expression in human MSCs via adenoviral gene transfer promotes cardiomyogenic differentiation and transplantation efficiency in murine ischemic heart injury models [45]. Co-expression of the human cytomegalovirus proteins US6 and US11 through retroviral vector-based transfection in human MSCs successfully switched off the recognition of MSCs by the immune system, thus allowing a higher level of productive engraftment in the murine liver after transplantation [46]. However, because viral gene transfer methods genetically drive host cell reprogramming by modulating the activities of target and neighboring genes at the insertion site, they raise safety issues regarding possible post-transplantation tumor development, especially in clinical applications [47]. Nonetheless, the transient overexpression of target gene(s) in MSCs by transfection with carefully conceived plasmids appears to be a relatively safe and technically feasible method. Indeed, as investigated in this study, this alternative approach can significantly improve the therapeutic outcomes in our clinically relevant ACLF model using Nrf2/DKK1-preconditioned MSCs. Moreover, long-term observation after transplantation did not suggest any adverse effects, which precluded the possibility of carcinogenesis.

In terms of mechanisms, recipient liver signaling cascades that are directly modulated by stem cell therapies after injury have long been an enigma. Stem cells have been proposed to orchestrate host hepatocyte regeneration via direct homing, replenishment of functional hepatocytes, and paracrine actions (e.g. via secretion of proteins and extracellular vesicles for cell-to-cell communication) [48, 49]. CKAP4 and LRP6 have been reported to be direct DKK1 receptors implicated in cancer cell proliferation, with similar affinities but distinct cysteine-rich domains [39]. In this study, we investigated which receptors matter and found that CKAP4, but not LRP6, mediates DKK1-induced host hepatocyte regeneration partly through Akt activation in an ACLF hepatotoxicity model. Other DKK1 targets in hepatocytes that could contribute to this intricate process warrant further investigation. Importantly, however, abundant DKK1 expression may promote hepatocellular carcinoma cell migration and invasion, and serve as a protein biomarker of liver cancers [50, 51]. Thus, the transient overexpression of DKK1 as an intervention strategy should be viewed with caution in patients with such cancers. Insights gained on the related signaling pathways underpinning the anti-stress capacity of MSCs will definitively help improve the efficacy and technical maturity of MSC-based transplantation therapies for other types of intractable clinical diseases. Recently, several clinical trials have been conducted using stem cells, most of which have demonstrated the safety and efficiency of stem cell-based therapies [52, 53]. However, despite the great potential of stem cells to treat diseases, stem cell-based therapies have not been shown to be safe or effective and may be accompanied by very serious health risks.

This study had several limitations. Although MSCs have minimal immunogenicity and low tumorigenesis, the safety of transient gene manipulated-stem cell transplantation requires long-term observation in clinical trials [54]. Another question is whether MSCs can be localized to the target tissues after *in vivo* administration. Many studies have shown the chemotaxis of infused MSCs toward injured or inflammatory sites [55–57]. In summary, MSC transplantation offers a promising outlook for treating various diseases, capitalizing on the versatile capabilities of MSCs. Advancements may focus on refining procedures and tailoring MSC functions, providing more effective and personalized treatments in the future. The evolving landscape of MSC-based therapies holds the potential for transformative breakthroughs in medical interventions.

## Conclusions

We found that the Nrf2/DKK1 signaling pathway sustains and enhances MSC resilience against extrinsically imposed stress post-transplantation. The MAPK proteins p38 and ERK are also involved in this process. MSC preconditioning by transiently inducing co-overexpression of Nrf2 and DKK1 via plasmids efficiently and safely improved the transplantation efficacy and therapeutic outcomes of human MSCs in a murine ACLF model. Our findings could lay a theoretical foundation for further innovations in translationally mature MSC-based therapies for liver diseases.

## Supplementary Material

goae016_Supplementary_Data
