# Supplementary material for: Invigorating human MSCs for transplantation therapy via Nrf2/DKK1 co-stimulation in an acute-on-chronic liver failure mouse model
Source: Gastroenterol Rep (Oxf). 2024 Mar 25;12:goae016. doi: 10.1093/gastro/goae016 (PMC10963075; doi:10.1093/gastro/goae016)
Supplement: goae016_Supplementary_Data [file goae016_supplementary_data.zip › Supplementary_file_2.docx]

**Invigorating human MSCs for transplantation therapy via Nrf2/DKK1 co-stimulation in a mice acute-on-chronic liver failure model**

**Running title:** Nrf2/DKK1 costimulation using MSCs in ACLF

Feng Chen^1,2†^, Zhaodi Che^3†^, Yingxia Liu^2^, Pingping Luo^3^, Lu Xiao^3^, Yali Song^3^, Cunchuan Wang^3^, Zhiyong Dong^3^, Mianhuan Li^2^, George L. Tipoe^4^, Min Yang^2^, Yi Lv^5^, Hong Zhang^6^, Fei Wang^1*^, and Jia Xiao^3,6,7*^

^1^Division of Gastroenterology, Seventh Affiliated Hospital of Sun Yat-sen University, Shenzhen, Guangdong, P. R. China

^2^National Clinical Research Center for Infectious Diseases, Second Affiliated Hospital of Southern University of Science and Technology, Shenzhen, Guangdong, P. R. China

^3^Clinical Medicine Research Institute and Department of Metabolic and Bariatric Surgery, The First Affiliated Hospital of Jinan University, Guangzhou, Guangdong, P. R. China

^4^School of Biomedical Sciences, The University of Hong Kong, Hong Kong SAR, P. R. China

^5^Laboratory of Neuroendocrinology, Fujian Key Laboratory of Developmental and Neurobiology, School of Life Sciences, Fujian Normal University, Fuzhou, Fujian, P. R. China.

^6^Department of Surgery, The Sixth Affiliated Hospital of Jinan University, Jinan University, Dongguan, Guangdong, P. R. China

^7^Shandong Provincial Key Laboratory for Clinical Research of Liver Diseases, Qingdao Hospital, University of Health and Rehabilitation Sciences, Qingdao, Shandong, P. R. China.

^†^These authors contributed equally to this work.

**Correspondence**

*Jia Xiao
Clinical Medicine Research Institute and Department of Metabolic and Bariatric Surgery, The First Affiliated Hospital of Jinan University, 613 Huangpu Avenue West, Guangzhou, Guangdong 510632, P. R. China.
Tel: +86-18520228386; Fax: +86-18520228386;
Email: [edwinsiu@connect.hku.hk](mailto:edwinsiu@connect.hku.hk) (J.X.)

*Fei Wang
Division of Gastroenterology, Seventh Affiliated Hospital of Sun Yat-sen University, 628 Zhenyuan Road, Shenzhen, Guangdong 518107, P. R. China.
Tel: +86-13816601005; Fax: +86-0755-81206211;
Email: [wangf323@mail.sysu.edu.cn](mailto:wangf323@mail.sysu.edu.cn) (F.W.)

**The Full-length blots/gels for Figure 1a**


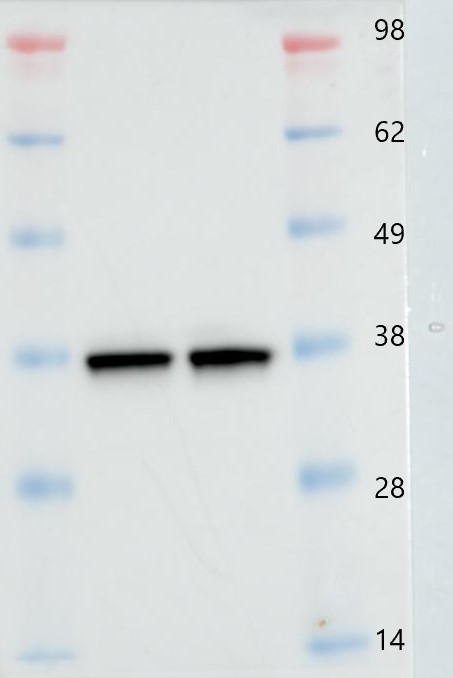


**GADPH: 36 kDa**


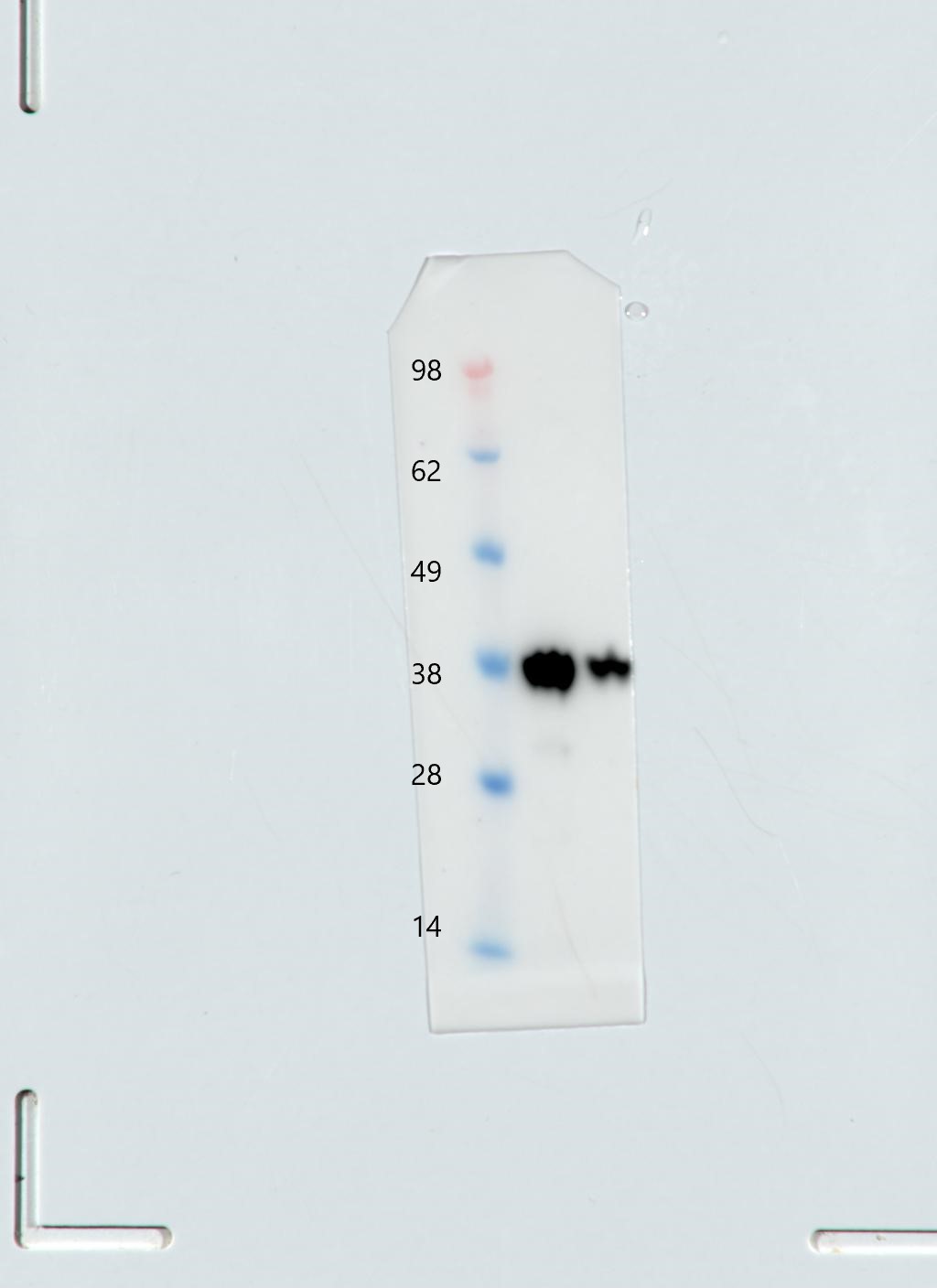


**sDKK1: 38kDa**


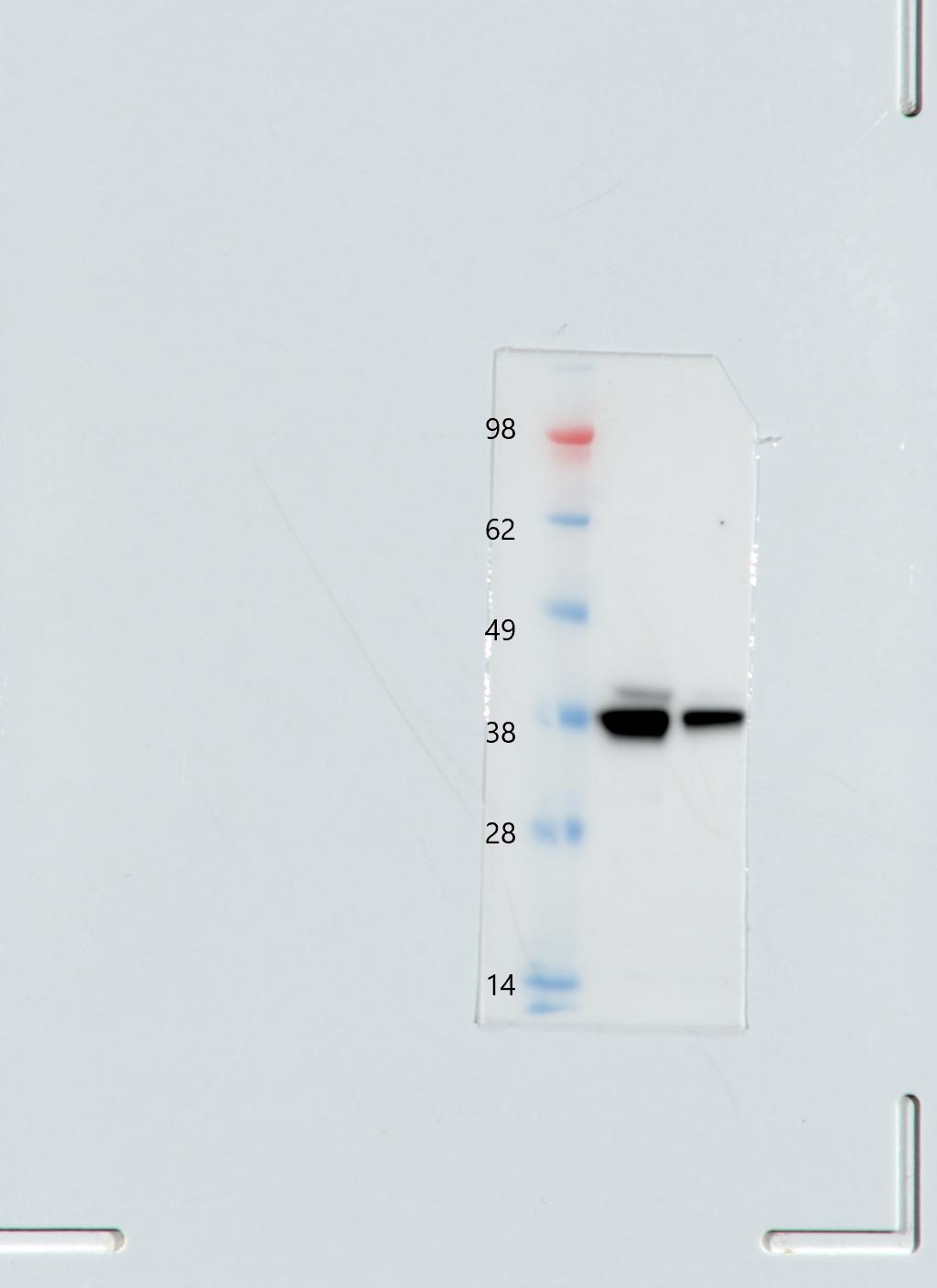


**DKK1: 38 kDa**

Ctrl

+ TNF-α/H_2_O_2_

+ TNF-α/H_2_O_2_

Ctrl

Ctrl

+ TNF-α/H_2_O_2_

**The Full-length blots/gels for Figure 1f**


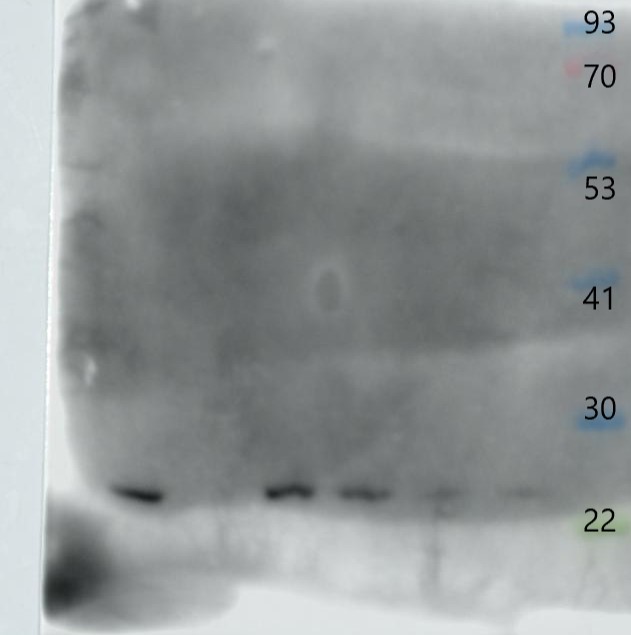

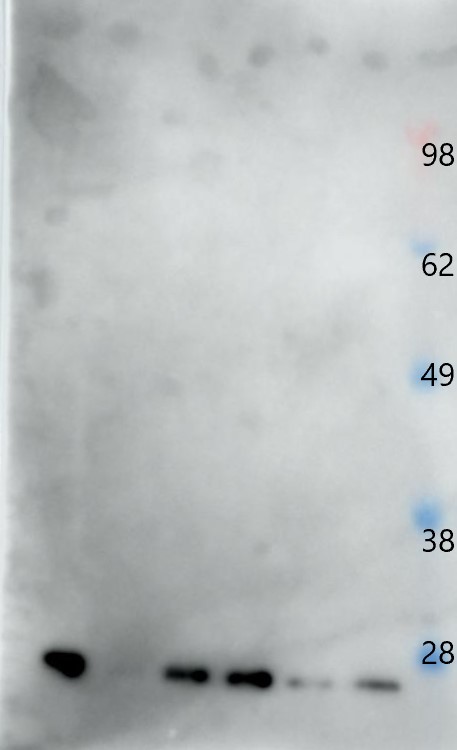

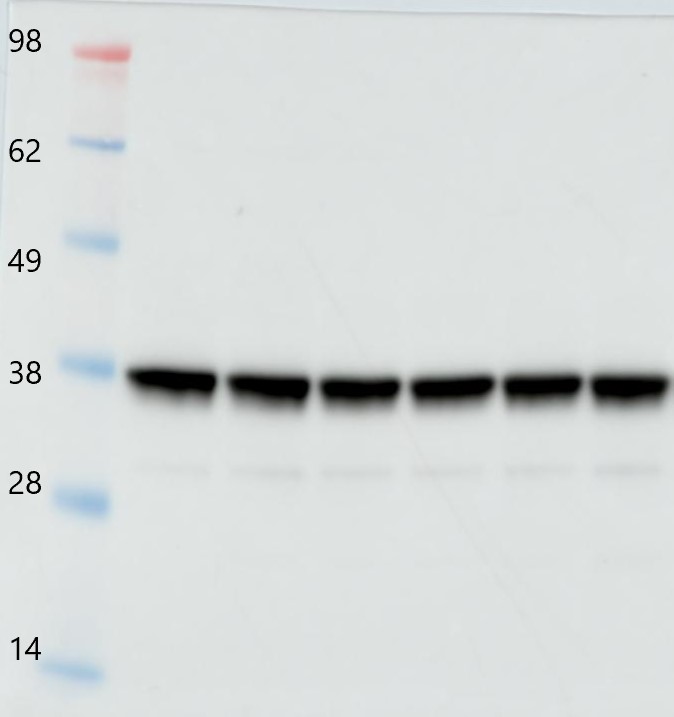


**GADPH: 36kDa**

**Bcl2: 26 kDa**

**PCNA: 29 kDa**

Ctrl

+ TNF-α/H_2_O_2_

+ TNF-α/H_2_O_2_ + Nrf2 OE

+ TNF-α/H_2_O_2_ + DKK1 OE

+ TNF-α/H_2_O_2_ + Nrf2 KD

+ TNF-α/H_2_O_2_ + DKK1 KD

+ TNF-α/H_2_O_2_ + DKK1 KD

+ TNF-α/H_2_O_2_ + Nrf2 KD

+ TNF-α/H_2_O_2_ + DKK1 OE

+ TNF-α/H_2_O_2_ + Nrf2 OE

+ TNF-α/H_2_O_2_

Ctrl

Ctrl

+ TNF-α/H_2_O_2_ + DKK1 OE

+ TNF-α/H_2_O_2_ + DKK1 KD

+ TNF-α/H_2_O_2_ + Nrf2 KD

+ TNF-α/H_2_O_2_ + Nrf2 OE

+ TNF-α/H_2_O_2_

**The Full-length blots/gels for Figure 2b**

**GADPH: 36kDa**

**Bcl2: 26 kDa**

**PCNA: 29 kDa**

**
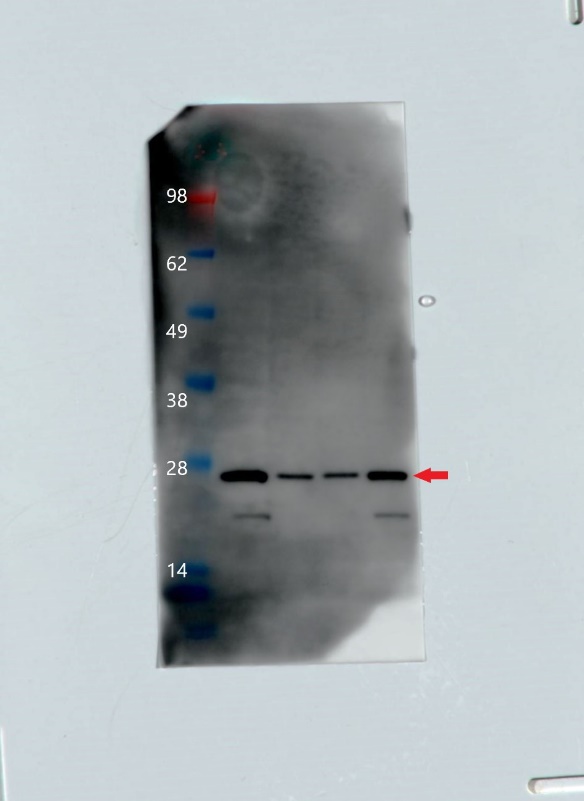

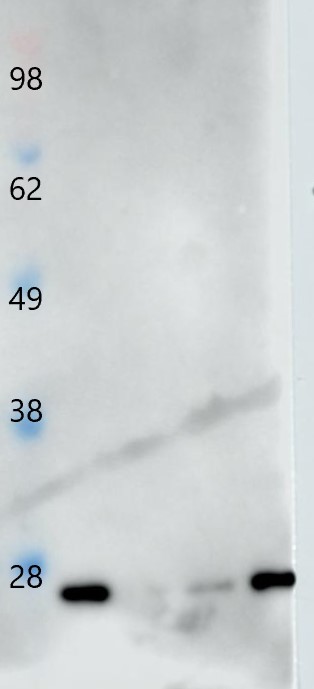

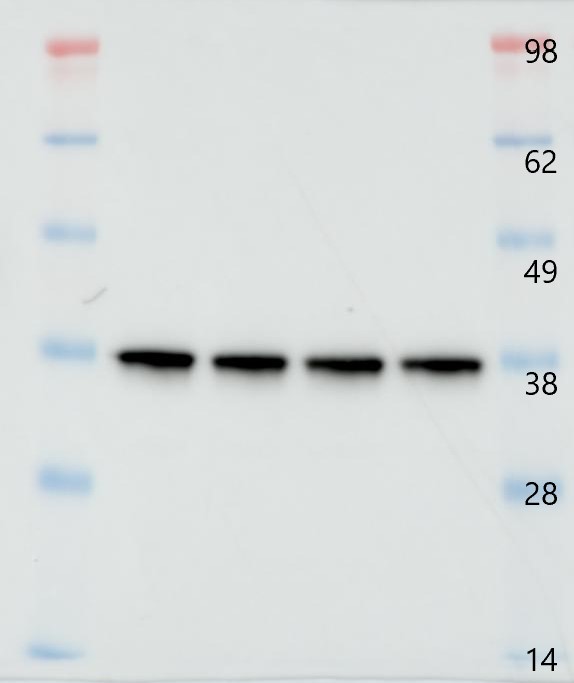
**


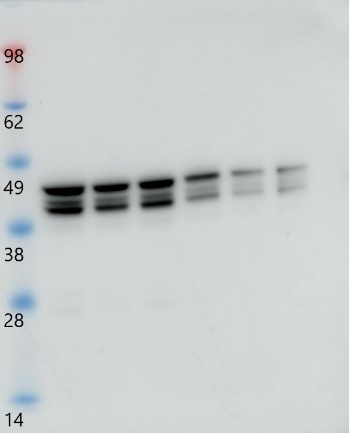

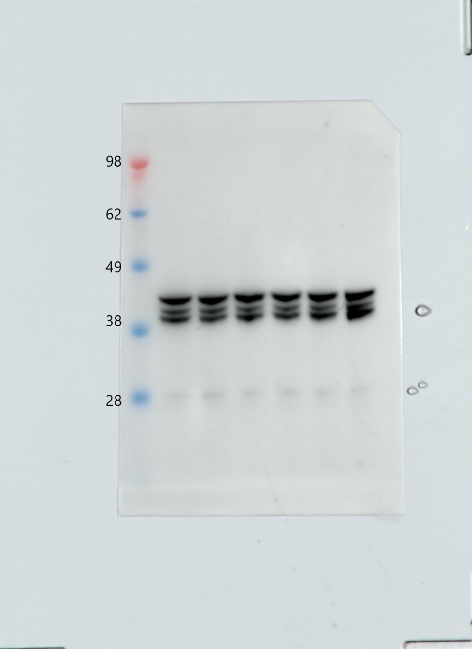
**The Full-length blots/gels for Figure 2d**

+ TNF-α/H_2_O_2_

Ctrl

+ TNF-α/H_2_O_2_ + MitoQ

+ TNF-α/H_2_O_2_ + NAC

+ TNF-α/H_2_O_2_ + NAC

+ TNF-α/H_2_O_2_ + MitoQ

+ TNF-α/H_2_O_2_

Ctrl

+ TNF-α/H_2_O_2_ + NAC

+ TNF-α/H_2_O_2_ + MitoQ

+ TNF-α/H_2_O_2_

Ctrl

**t-ERK: 41/44 kDa**

**p-ERK: 42/44 kDa**

**GADPH: 36 kDa**

**DKK1: 38 kDa**

**t-P38: 41 kDa**

**p-P38: 41 kDa**


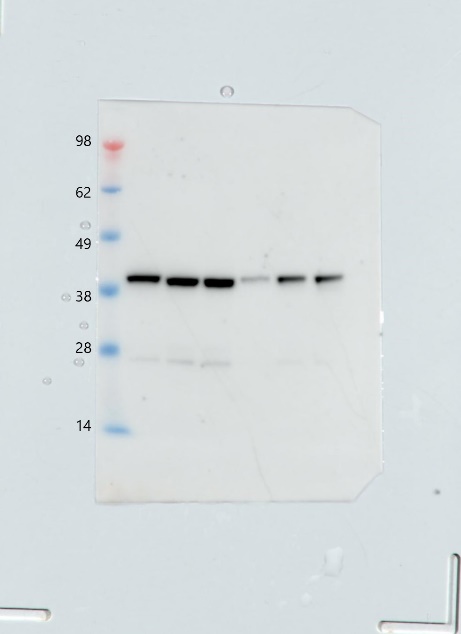

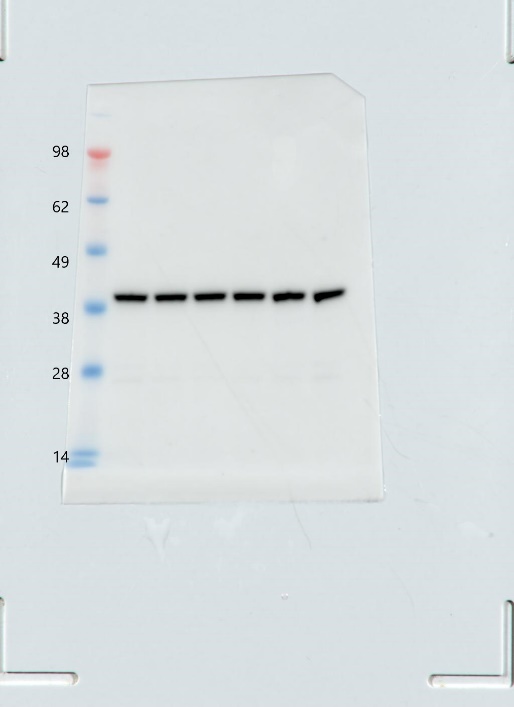

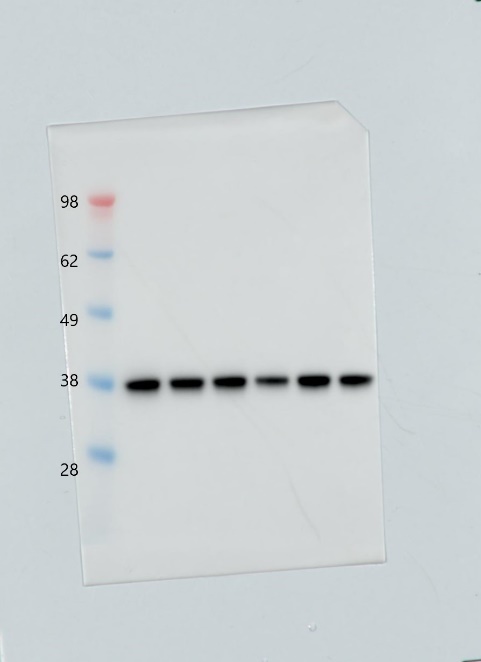

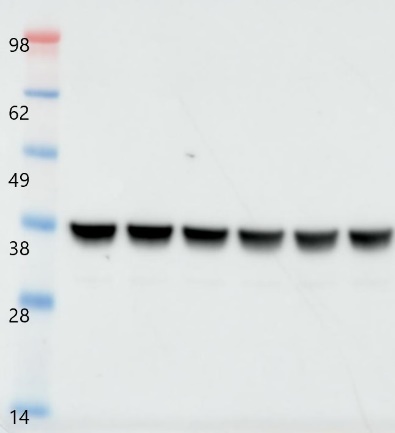


Ctrl

+ MitoQ

+ NAC

+ TNF-α/H_2_O_2_

+ TNF-α/H_2_O_2_ + MitoQ

+ TNF-α/H_2_O_2_ +NAC

+ MitoQ

+ NAC

+ TNF-α/H_2_O_2_

+ TNF-α/H_2_O_2_ + MitoQ

+ TNF-α/H_2_O_2_ +NAC

+ TNF-α/H_2_O_2_

+ TNF-α/H_2_O_2_ + MitoQ

+ TNF-α/H_2_O_2_ +NAC

Ctrl

+ NAC

+ MitoQ

+ TNF-α/H_2_O_2_ +NAC

+ TNF-α/H_2_O_2_ + MitoQ

+ TNF-α/H_2_O_2_

+ NAC

+ MitoQ

Ctrl

Ctrl

+ TNF-α/H_2_O_2_ +NAC

+ TNF-α/H_2_O_2_ + MitoQ

+ TNF-α/H_2_O_2_

+ NAC

+ MitoQ

Ctrl

+ MitoQ

+ NAC

+ TNF-α/H_2_O_2_ +NAC

+ TNF-α/H_2_O_2_ + MitoQ

Ctrl

+ TNF-α/H_2_O_2_

**The Full-length blots/gels for Figure 2e**


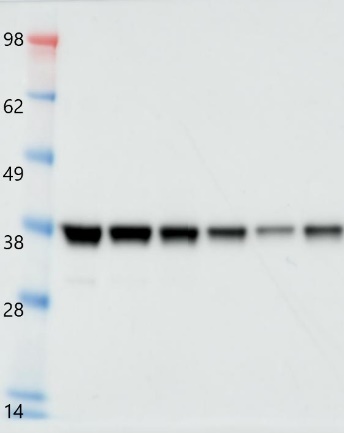

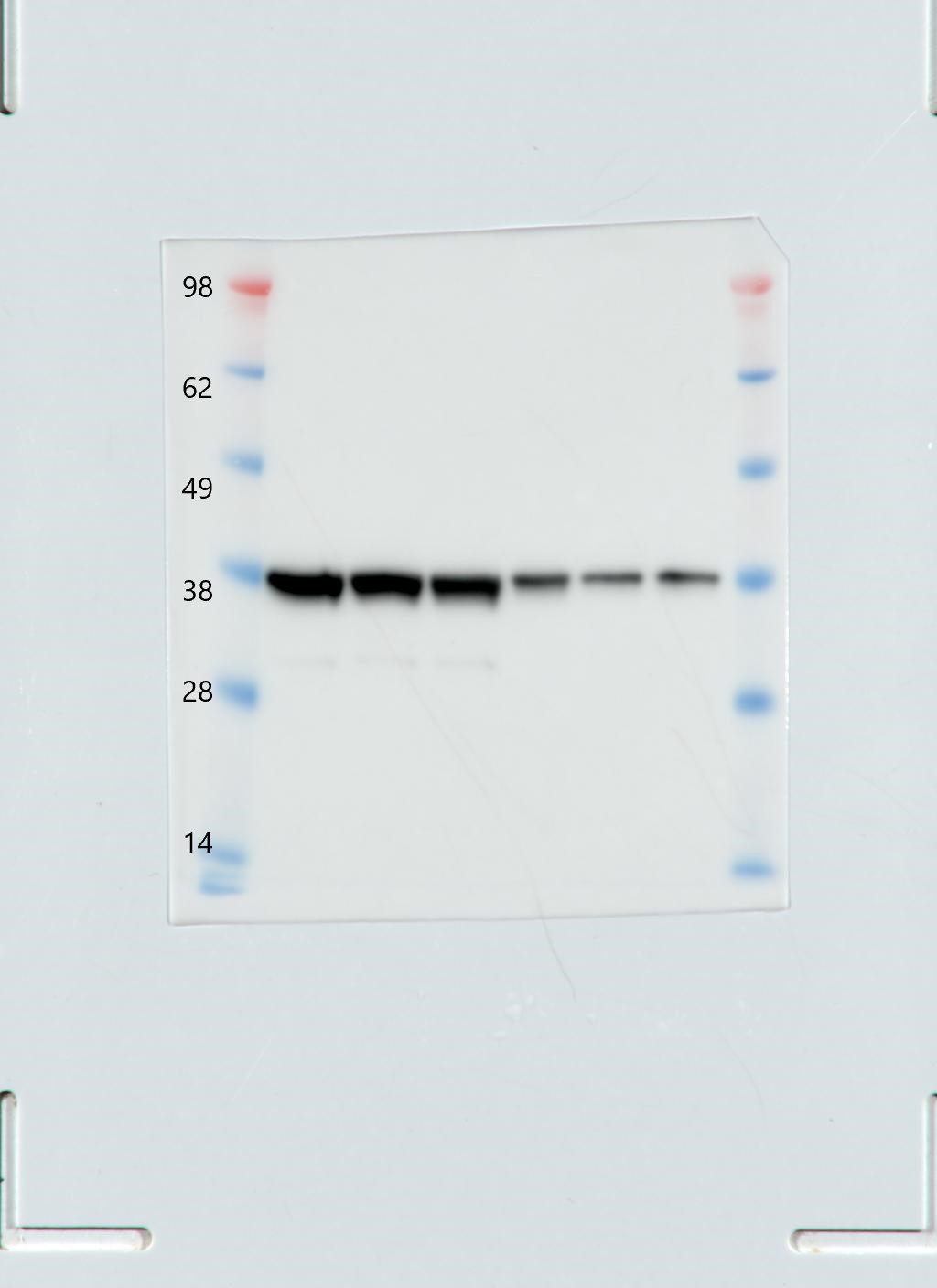

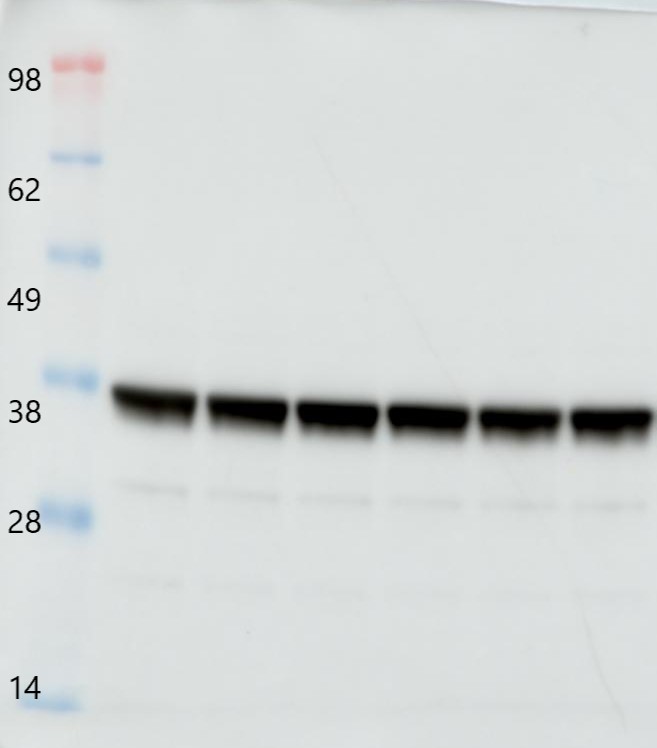


**GADPH: 36 kDa**

**sDKK1: 38 kDa**

**DKK1: 38 kDa**

+ TNF-α/H_2_O_2_

Ctrl

+ TNF-α/H_2_O_2_ + SB

+ TNF-α/H_2_O_2_ + U0

+ U0

+ SB

+ TNF-α/H_2_O_2_ + U0

+ TNF-α/H_2_O_2_ + SB

+ TNF-α/H_2_O_2_

+ U0

+ SB

Ctrl

+ TNF-α/H_2_O_2_ + U0

+ TNF-α/H_2_O_2_ + SB

+ TNF-α/H_2_O_2_

Ctrl

+ SB

+ U0

**The Full-length blots/gels for Figure 2e**

**p-P38: 41 kDa**

**p-ERK: 42/44kDa**

**t-P38：41kDa**

**t-ERK: 42/44 kDa**

**GADPH 36 kDa**


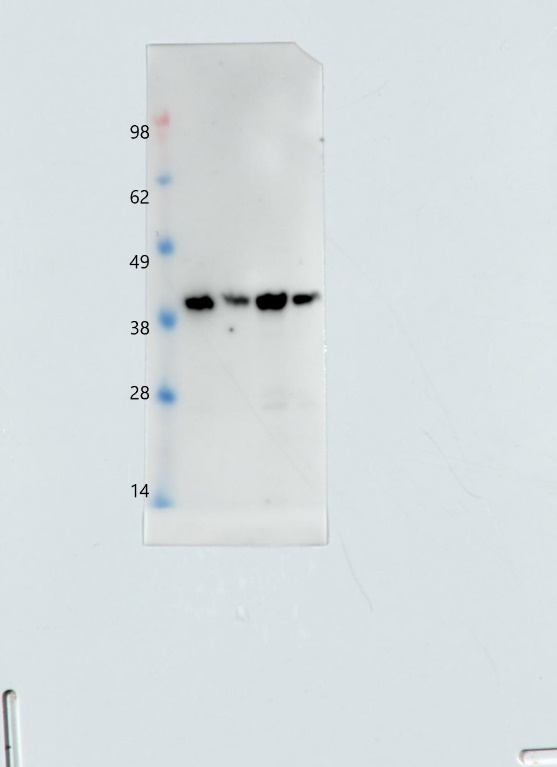

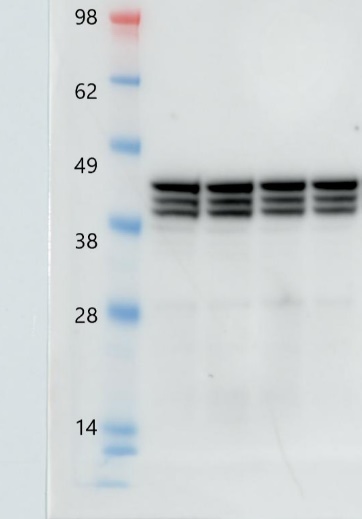

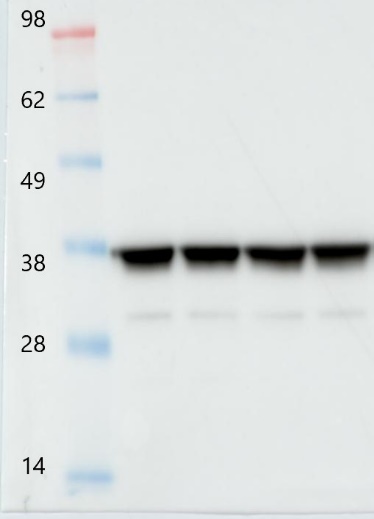

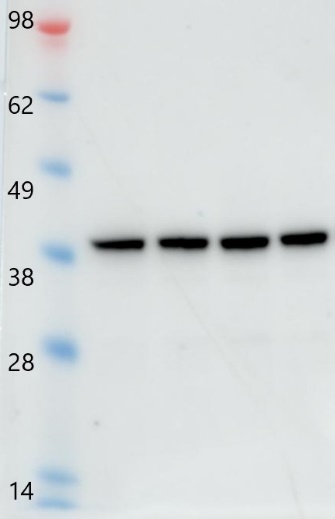

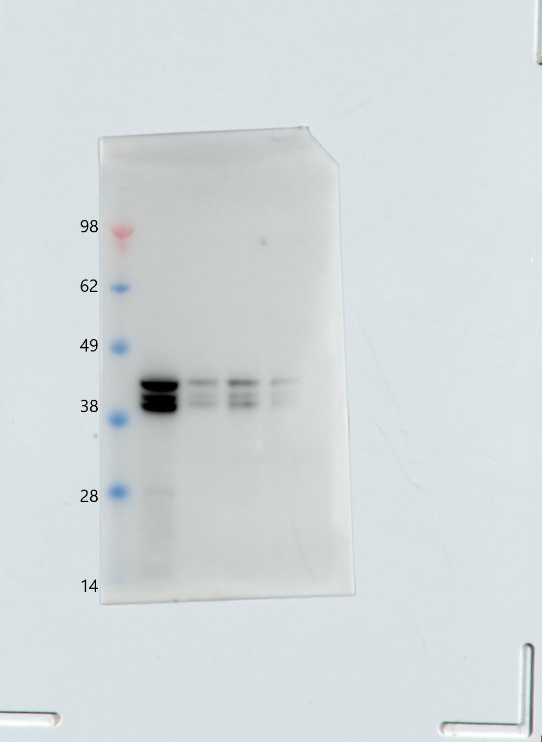


Ctrl

+ Nrf2 OE

+ TNF-α/H_2_O_2_

+ Nrf2 OE

+ TNF-α/H_2_O_2_

+ TNF-α/H_2_O_2_

+ Nrf2 OE

+ Nrf2 OE

+ TNF-α/H_2_O_2_

+ TNF-α/H_2_O_2_

+ Nrf2 OE

Ctrl

+ TNF-α/H_2_O_2_ + Nrf2 OE

+ TNF-α/H_2_O_2_ + Nrf2 OE

Ctrl

+ TNF-α/H_2_O_2_ + Nrf2 OE

+ TNF-α/H_2_O_2_ + Nrf2 OE

Ctrl

Ctrl

+ TNF-α/H_2_O_2_ + Nrf2 OE

**The Full-length blots/gels for Figure 2e**

**t-ERK: 42/44 kDa**

**p-ERK: 42/44 kDa**

**GADPH 36 kDa**

**p-P38: 41 kDa**


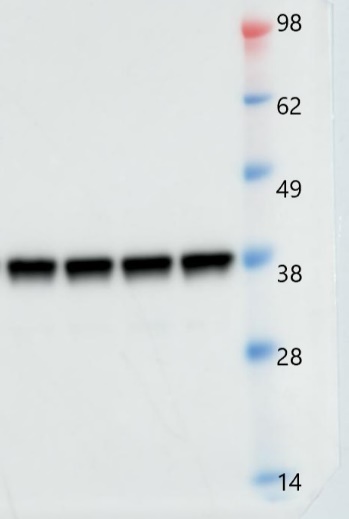


**t-P38：41 kDa**


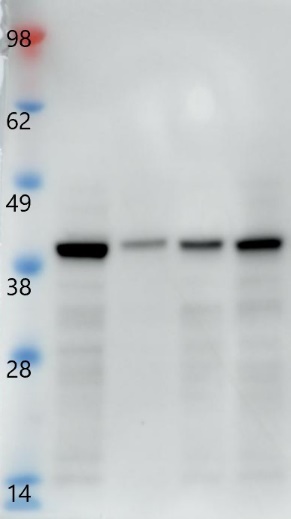


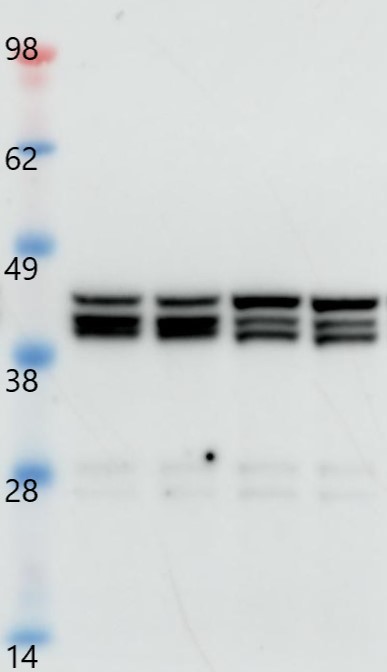

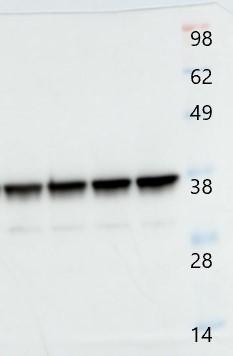

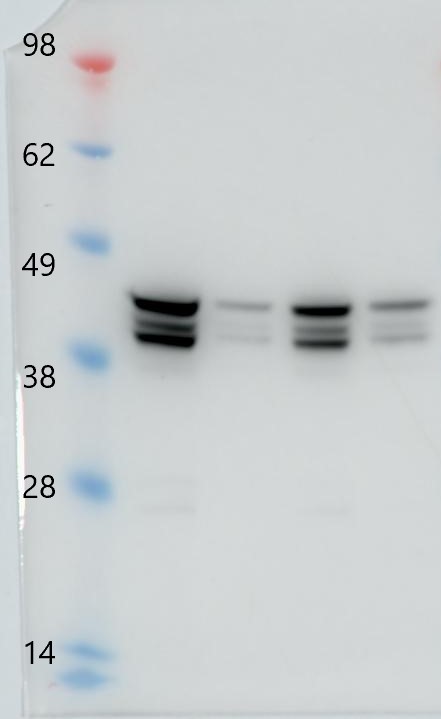


Ctrl

Ctrl

Ctrl

+ TNF-α/H_2_O_2_

+ DKK1 OE

Ctrl

+ DKK1 OE

+ TNF-α/H_2_O_2_

+ TNF-α/H_2_O_2_

+ DKK1 OE

+ DKK1 OE

+ TNF-α/H_2_O_2_

+ TNF-α/H_2_O_2_

+ DKK1 OE

+ TNF-α/H_2_O_2_ + DKK1 OE

+ TNF-α/H_2_O_2_ + DKK1 OE

+ TNF-α/H_2_O_2_ + DKK1 OE

+ TNF-α/H_2_O_2_ + DKK1 OE

Ctrl

+ TNF-α/H_2_O_2_ + DKK1 OE

**The Full-length blots/gels for Figure 3b**


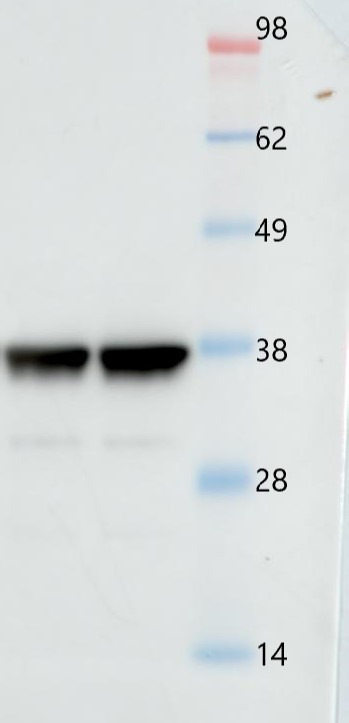

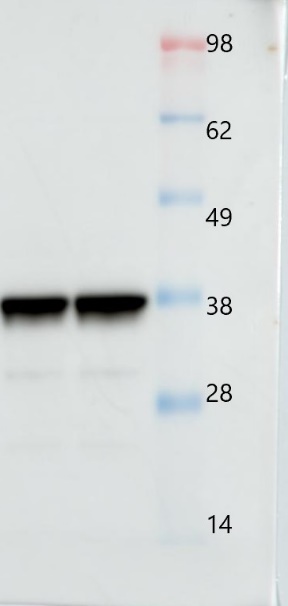

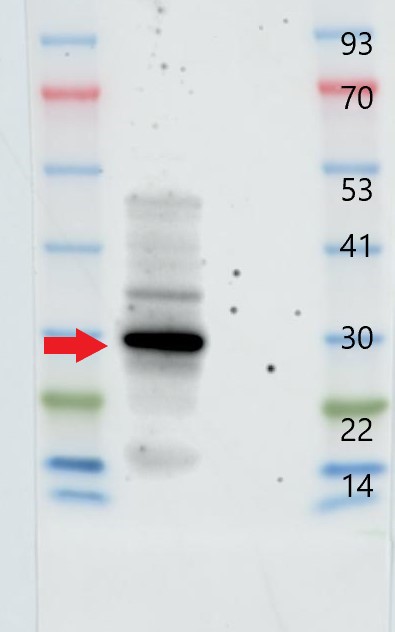

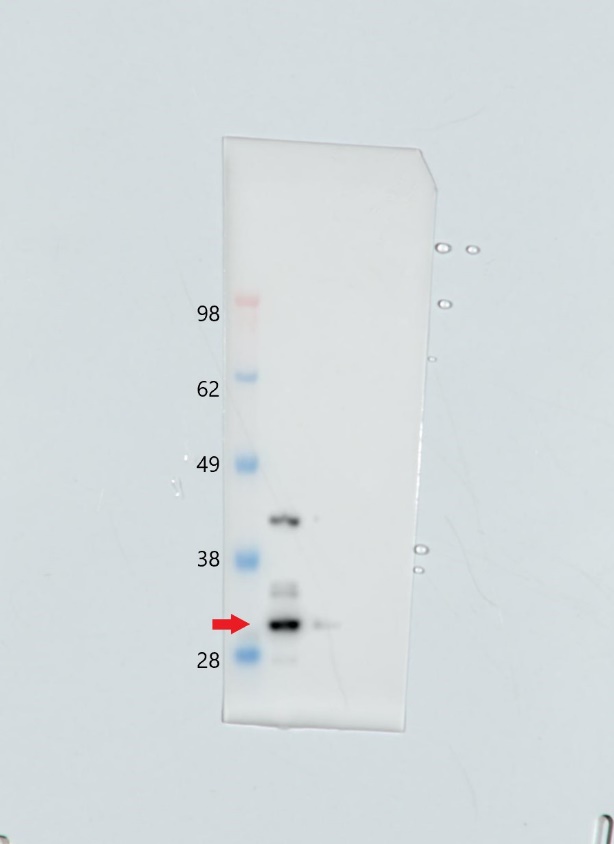


**GADPH: 36 kDa**

**HO-1: 32 kDa**

**NQO1: 31 kDa**

**GADPH: 36 kDa**

Scramble

HO-1 shRNA

HO-1 shRNA

Scramble

Scramble

NQO-1 shRNA

NQO-1 shRNA

Scramble

**The Full-length blots/gels for Figure 4b**

**
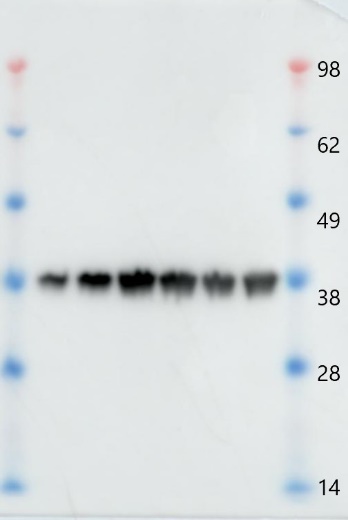

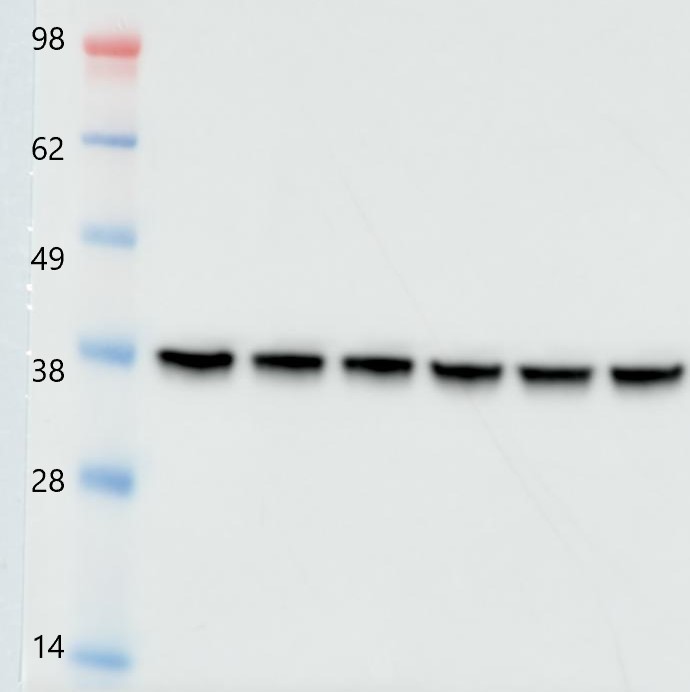

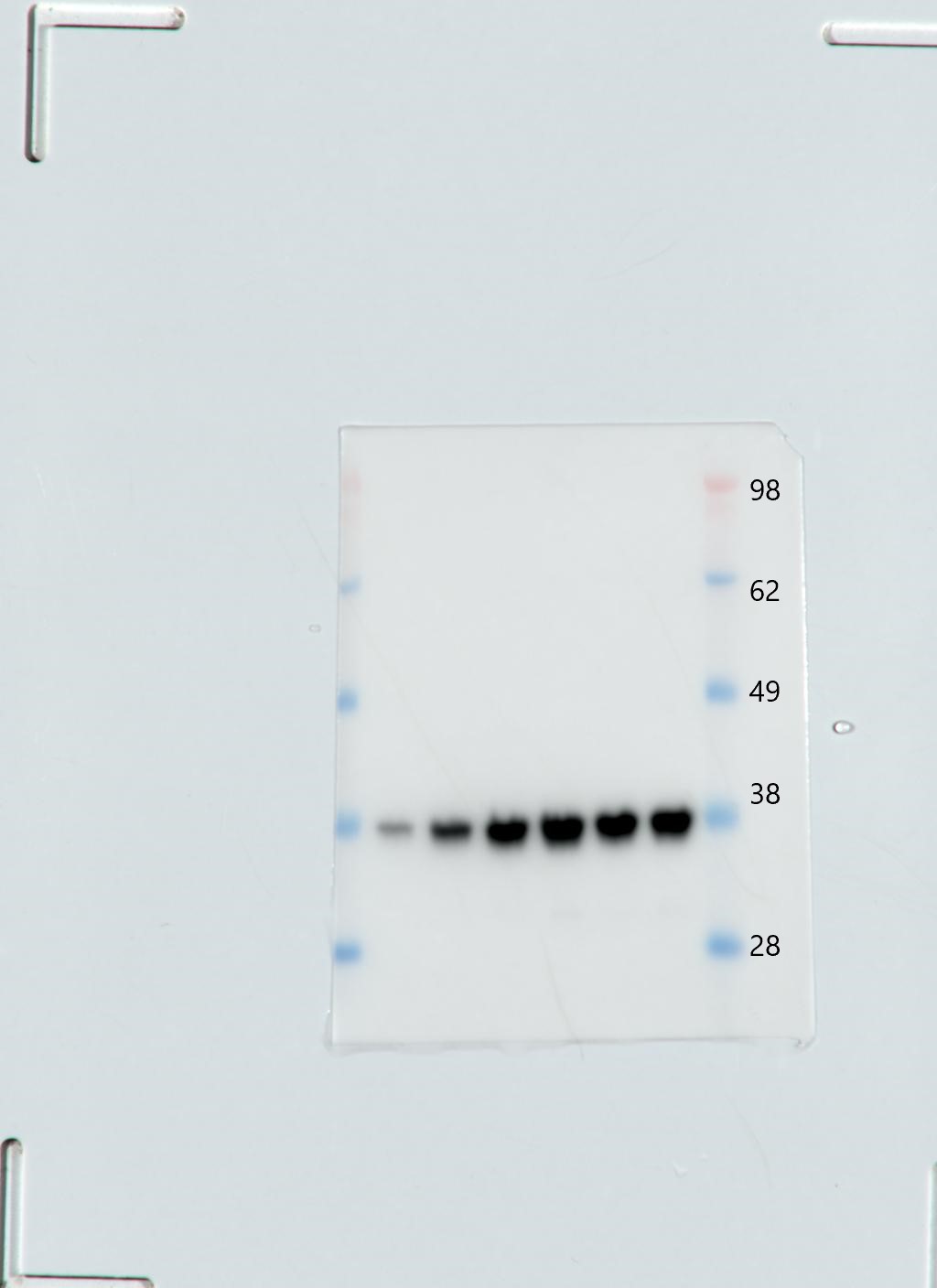
**
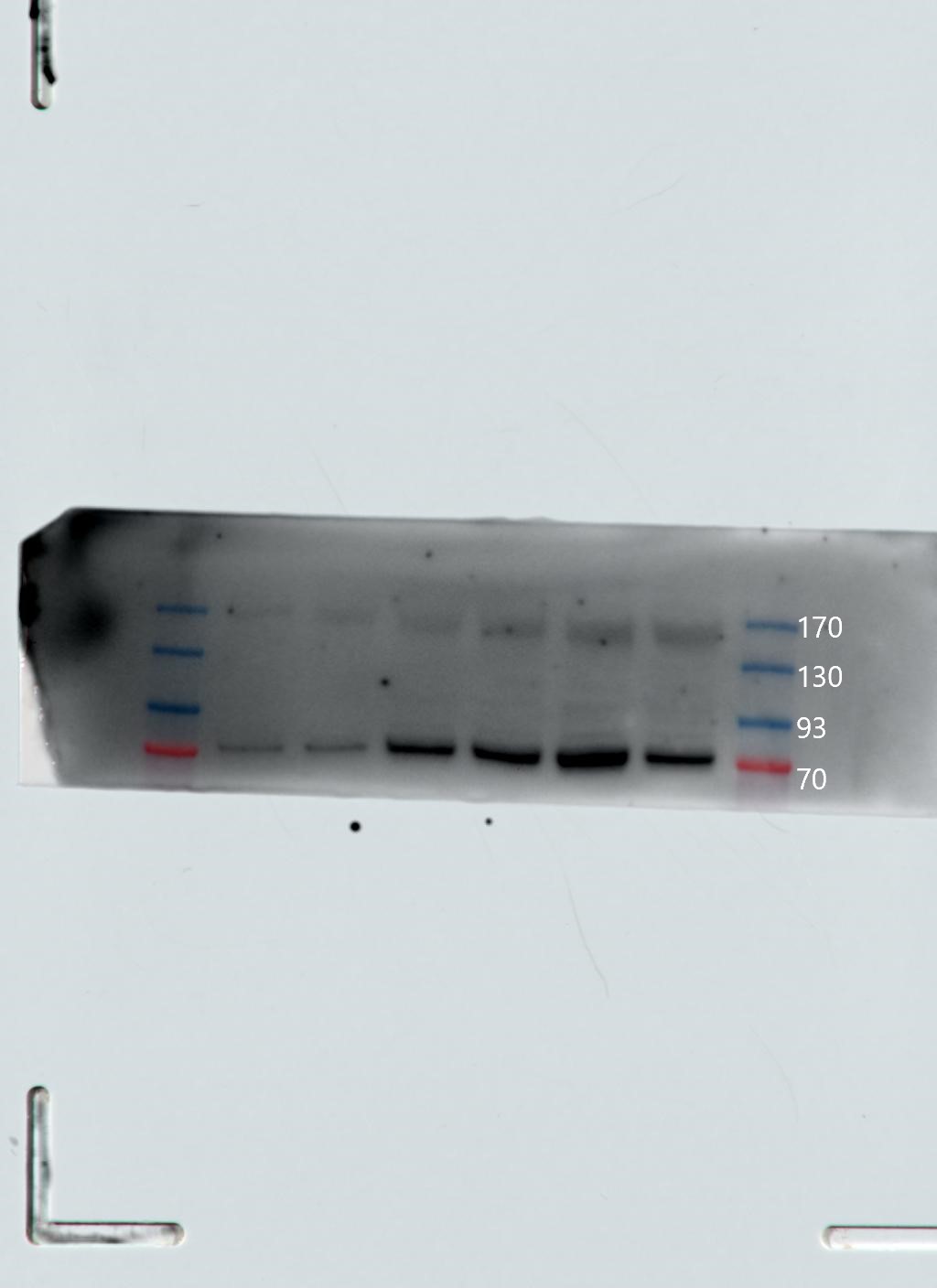


**NRF2: 80 kDa**

**DKK1:38 kDa**

**sDKK1: 38 kDa**

**GADPH: 36 kDa**

days post transfection

5

4

3

2

1

0

0

1

2

3

4

5

days post transfection

days post transfection

5

4

3

2

1

0

days post transfection

5

4

3

2

1

0

**
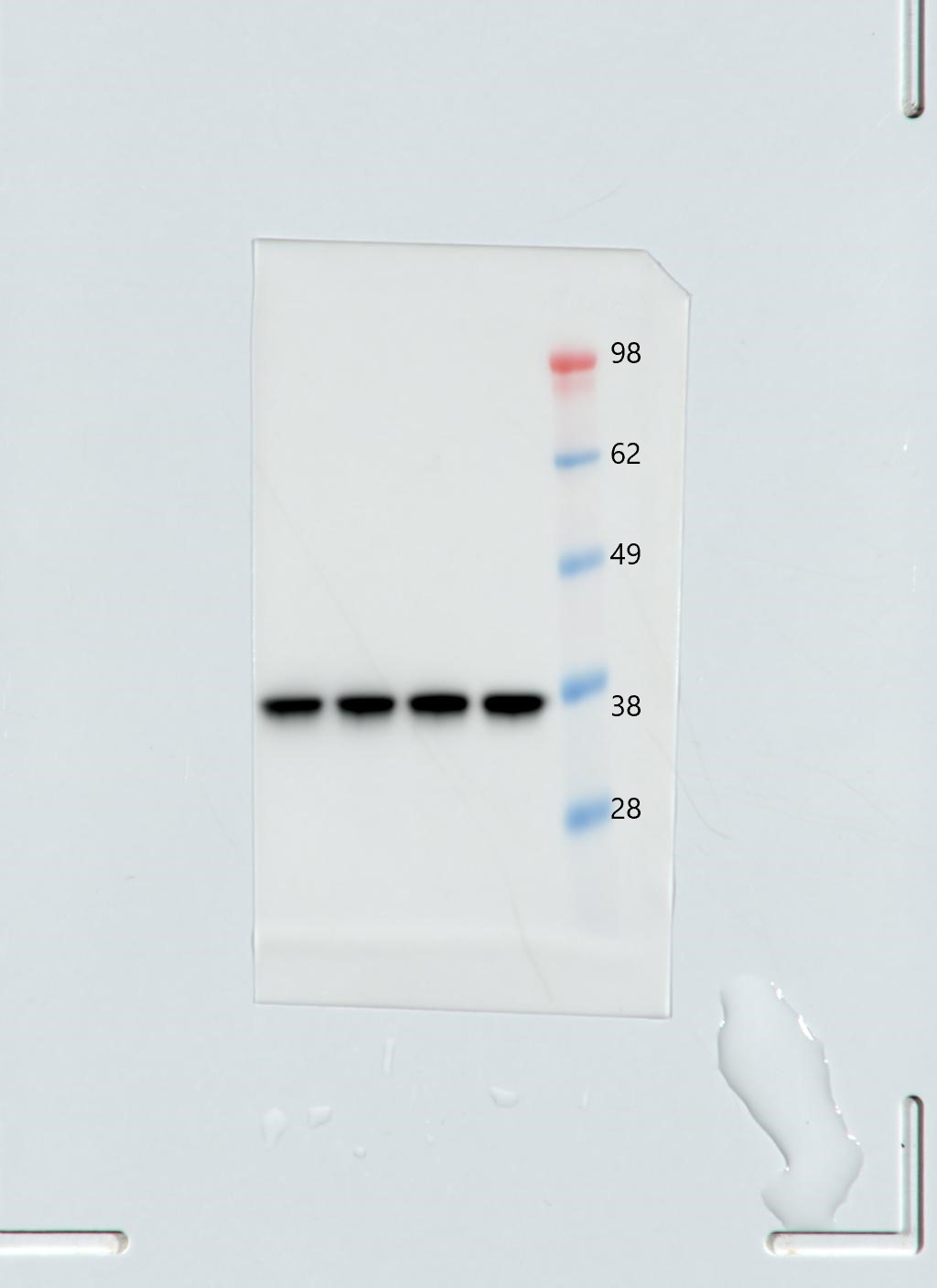

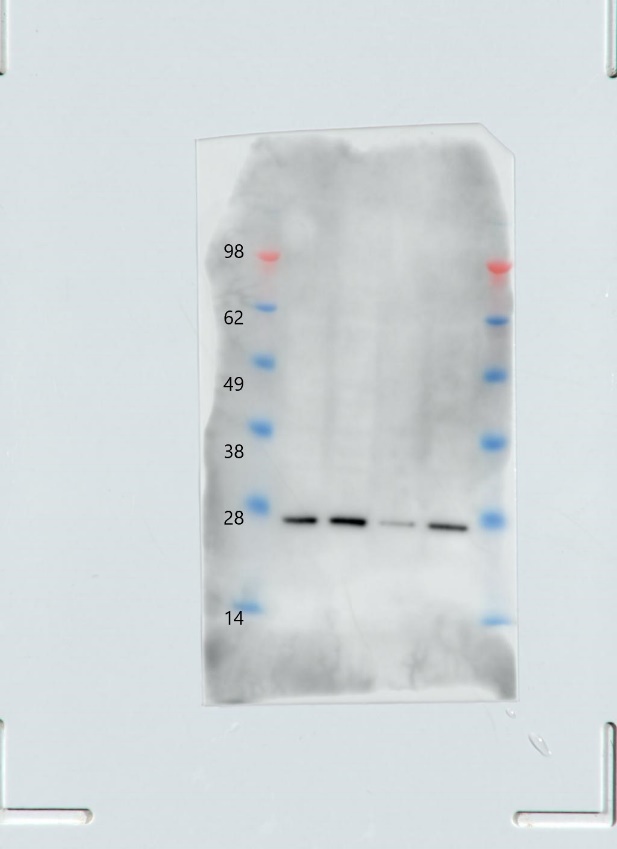

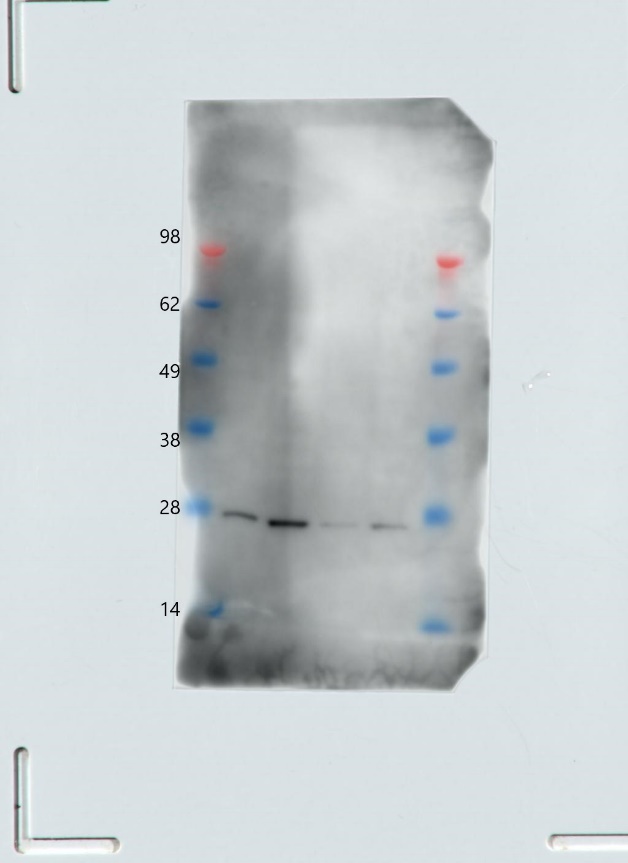
The Full-length blots/gels for Figure 4d**

*+ TNF-α/H_2_O_2_*

*+ TNF-α/H_2_O_2_*

*+ TNF-α/H_2_O_2_*

+

-

Plasmid

Plasmid

Plasmid

-

+

+

-

-

+

+

-

-

+

**GADPH: 36 kDa**

**Bcl2: 26 kDa**

**PCNA: 29 kDa**

**The Full-length blots/gels for Figure 5f**

**GADPH: 36 kDa**

**Human DKK1: 38 kDa**

**Human αAT: 52 kDa**


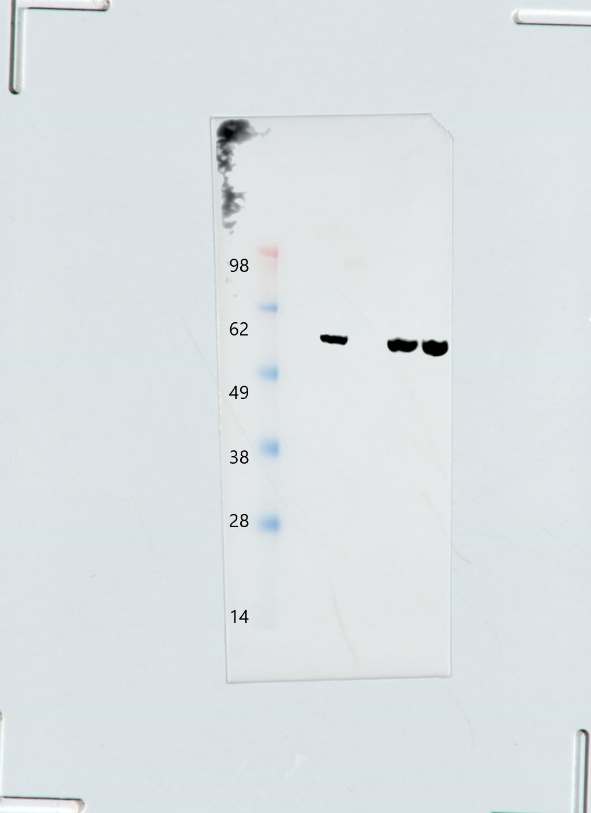

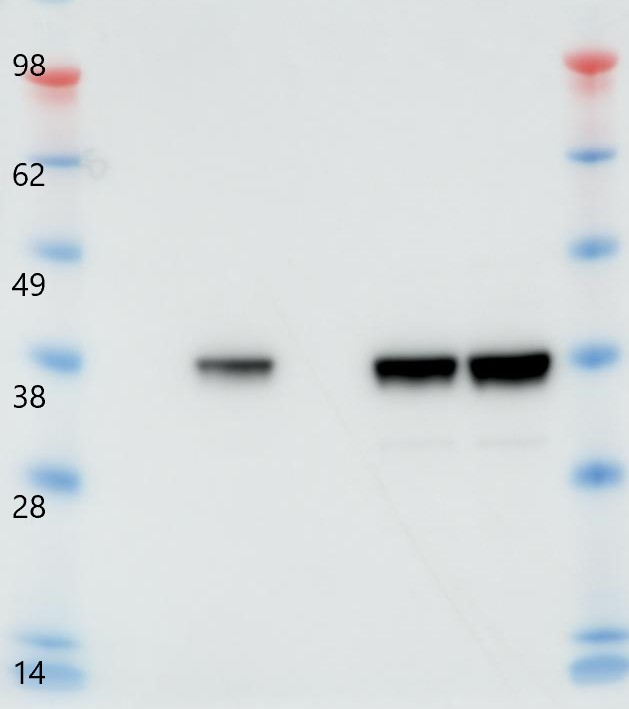

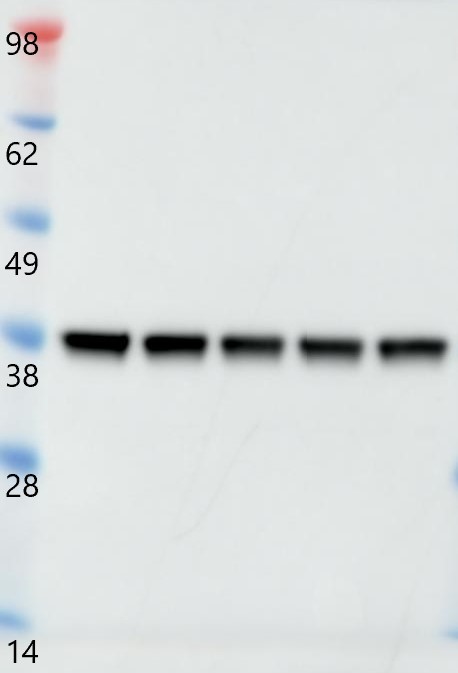


ACLF+MSC+ Plasmid

Healthy

ACLF+ MSC

Healthy+ MSC

ACLF

ACLF+ MSC+ Plasmid

ACLF+ MSC

ACLF

Healthy+ MSC

Healthy

ACLF+MSC+ Plasmid

ACLF+ MSC

ACLF

Healthy

Healthy+ MSC


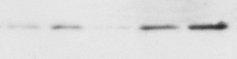


**The Full-length blots/gels for Figure 6c**


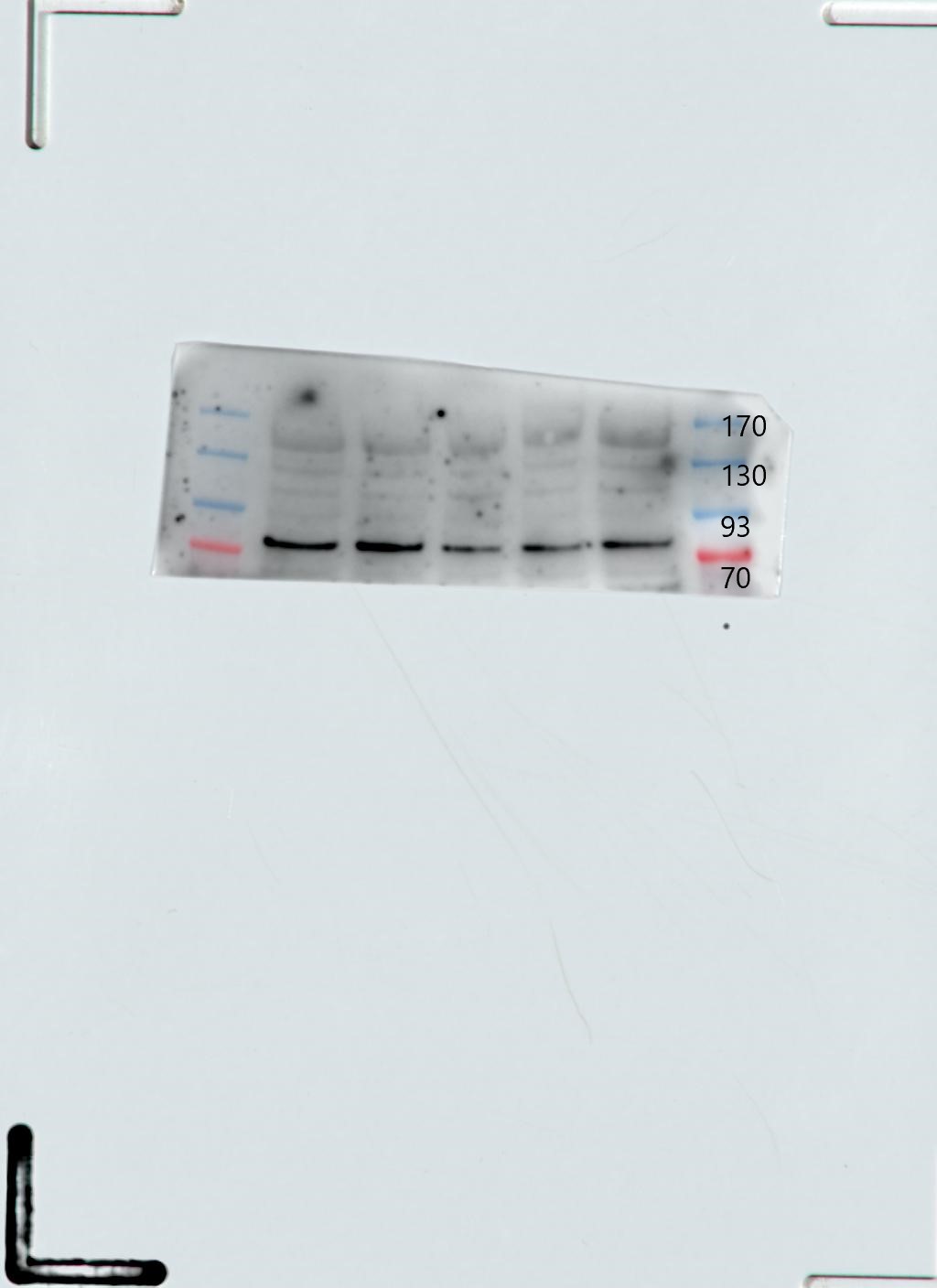


Healthy

Healthy+ MSC

ACLF

ACLF+ MSC

**STAT1: 87 kDa**

**p-STAT1: 87 kDa**


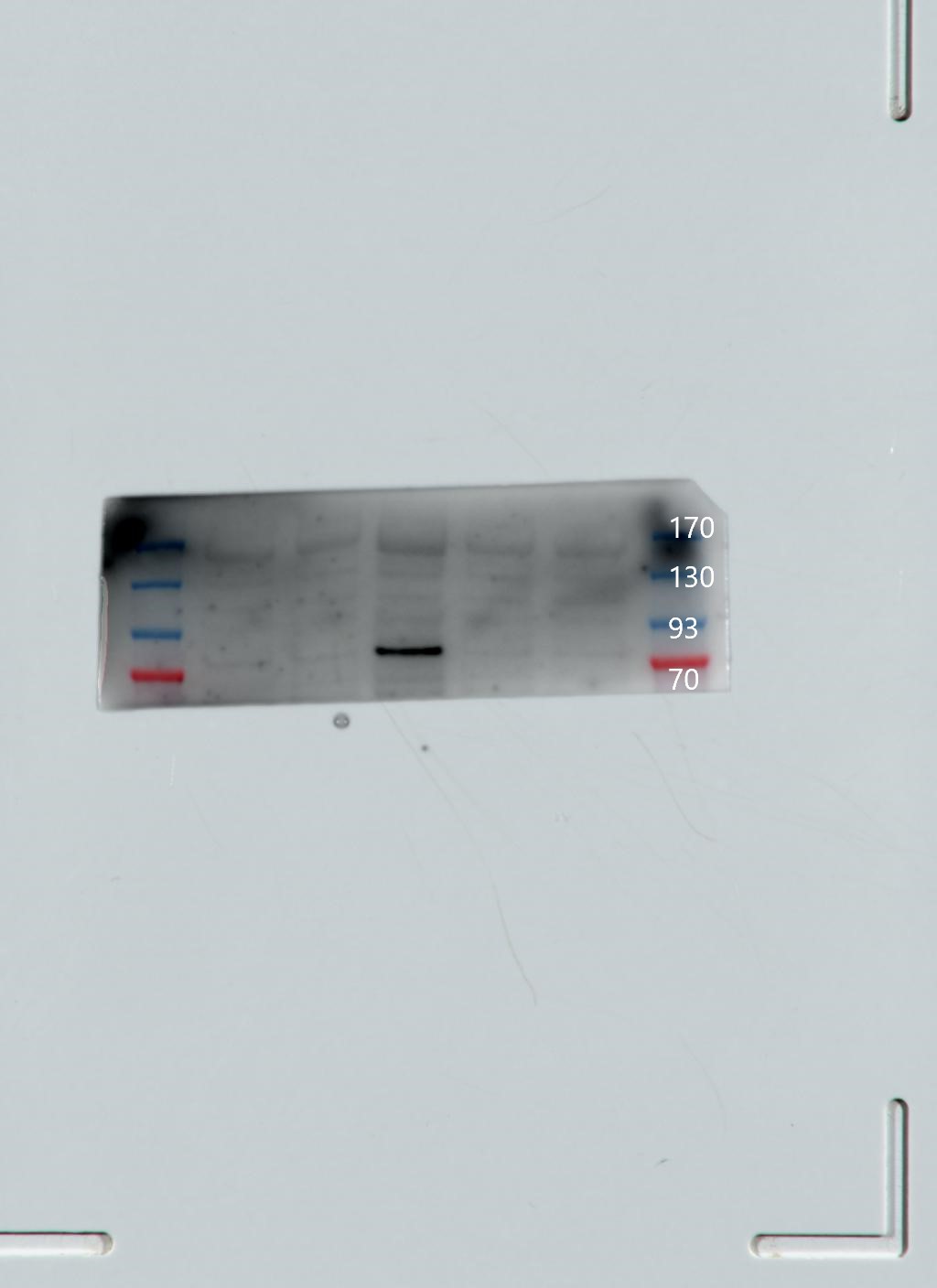
**The Full-length blots/gels for Figure 6c**

ACLF+MSC+ Plasmid

ACLF+MSC+ Plasmid

ACLF+ MSC

ACLF

Healthy+ MSC

Healthy

**
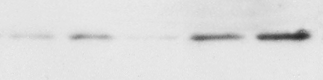

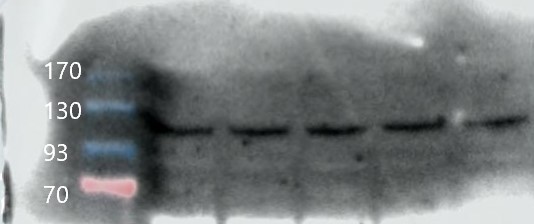
**

**STAT3-cropped: 88 kDa**

Healthy

Healthy+ MSC

ACLF

ACLF+ MSC

ACLF+ MSC+ Plasmid

ACLF+ MSC+ Plasmid

ACLF+ MSC

ACLF

Healthy

Healthy+ MSC

**p-STAT3: 88kDa**

**The Full-length blots/gels for Figure 6c**

**GADPH: 36 kDa**

**Cyclin D1: 36 kDa**


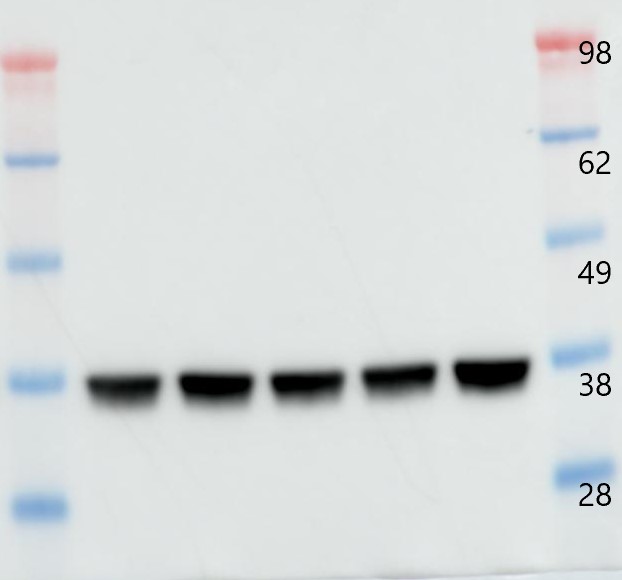

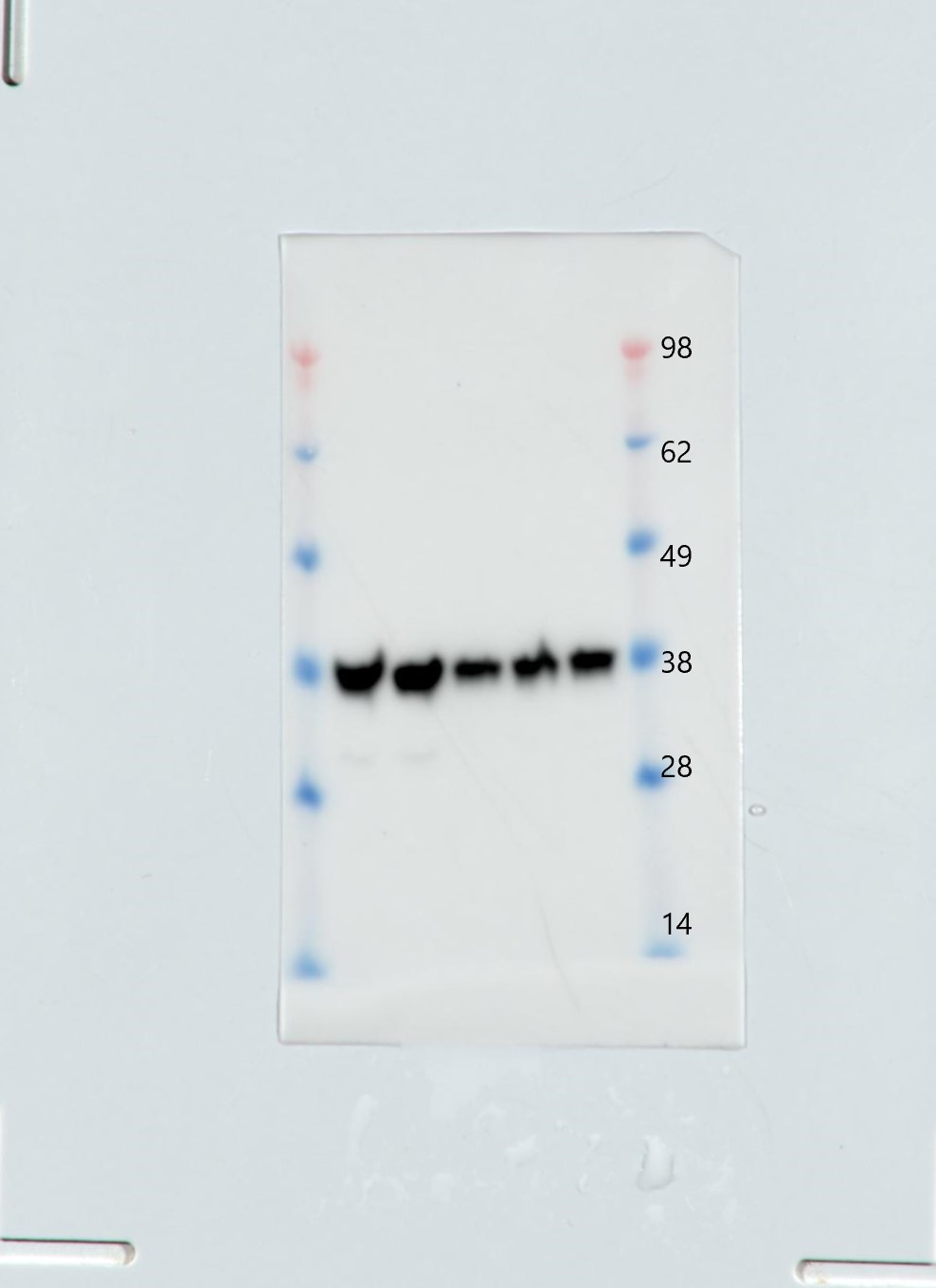


Healthy

Healthy+ MSC

ACLF

ACLF+ MSC

ACLF+ MSC+ Plasmid

ACLF+ MSC

ACLF+ MSC+ Plasmid

ACLF

Healthy+ MSC

Healthy

**The Full-length blots/gels for Figure 7a**

**GADPH**

**Mouse LPR6**

**Mouse CKAP4**


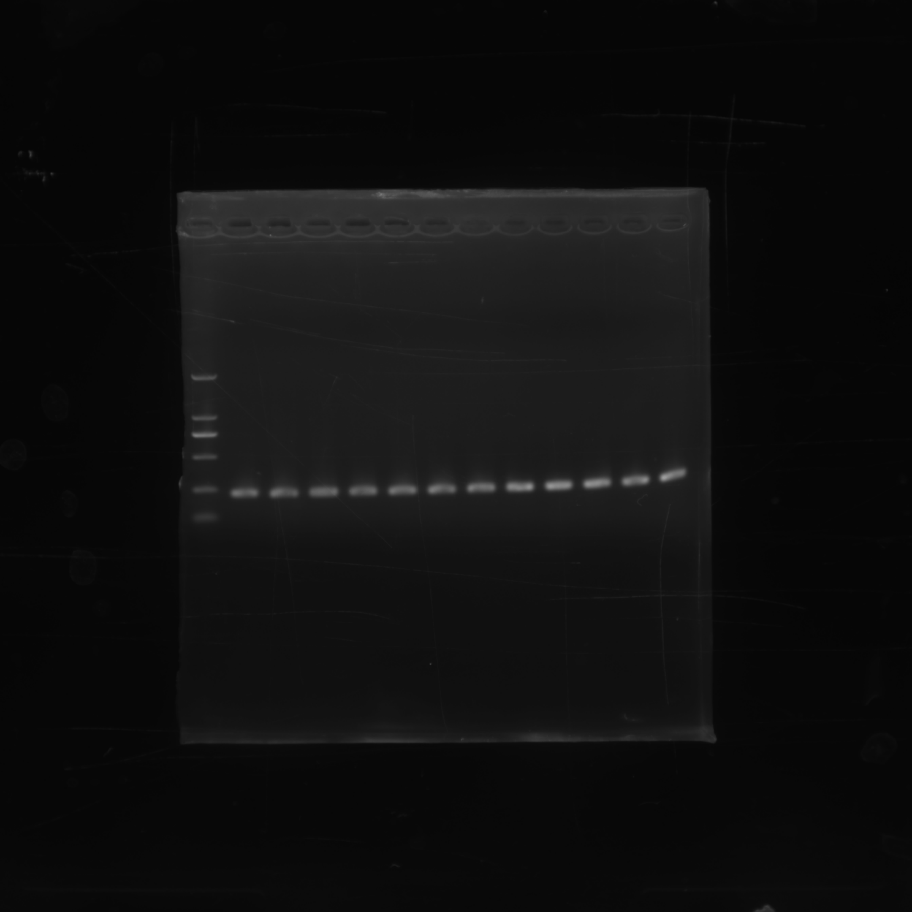

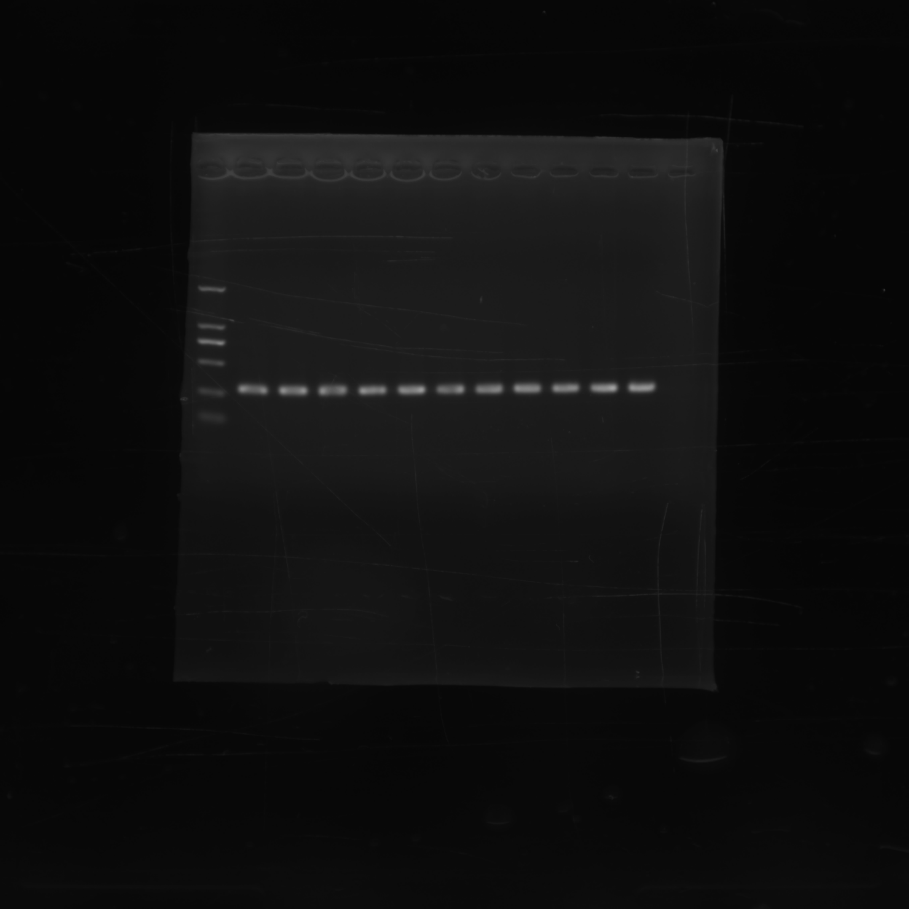
**
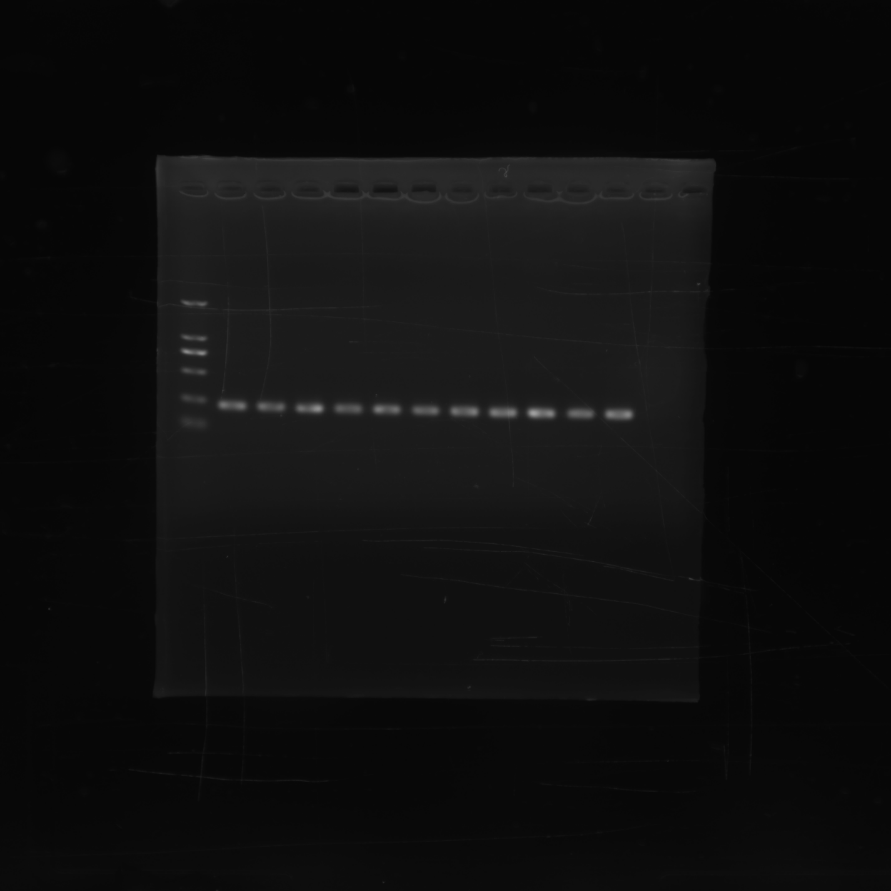
**

2000**-**

100**-**

250**-**

750**-**

1000**-**

500**-**

Liver

Brain

Kidney

Heart

Lung

Bone marrow

+Liver CKD

+Liver CKD

+Liver CKD

+Liver CKD

+Liver CKD

+Liver CKD

Liver

Brain

Kidney

Heart

Lung

Bone marrow

250**-**

100**-**

500**-**

2000**-**

750**-**

1000**-**

+Liver CKD

+Liver CKD

+Liver CKD

+Liver CKD

+Liver CKD

+Liver CKD

Liver

Brain

Kidney

Heart

Lung

Bone marrow

+Liver CKD

+Liver CKD

+Liver CKD

+Liver CKD

+Liver CKD

+Liver CKD

500**-**

750**-**

100**-**

250**-**

1000**-**

2000**-**

CKAP4 F Primer: TATGTCCATCACGTCCTGGAG, R Primer: CTAATTCGGTTGAGCTCGCTCT. The amplified fragment size is 227bp.

LRP6 F Primer: GCGAAGAAGCCATTAAACGAAC, R Primer: ACGTTCTATCTTTGGCACTTCT. The amplified fragment size is 284bp.

GADPH F Primer: AGGTCGGTGTGAACGGATTTG, R Primer: CTGGAAGATGGTGATGGGCTT. The amplified fragment size is 221bp.

**The Full-length blots/gels for Figure 7g -**Wild-type mice

**GADPH: 36 kDa**

**PCNA: 29 kDa**

**Cyclin D1：36 kDa**

**
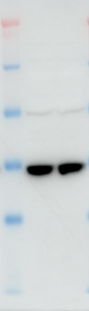
**
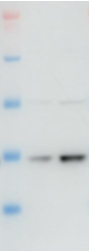
**
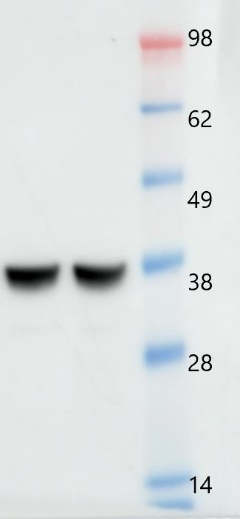

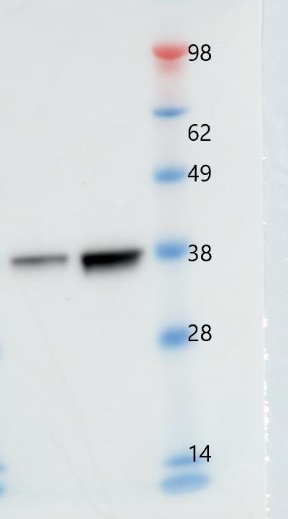

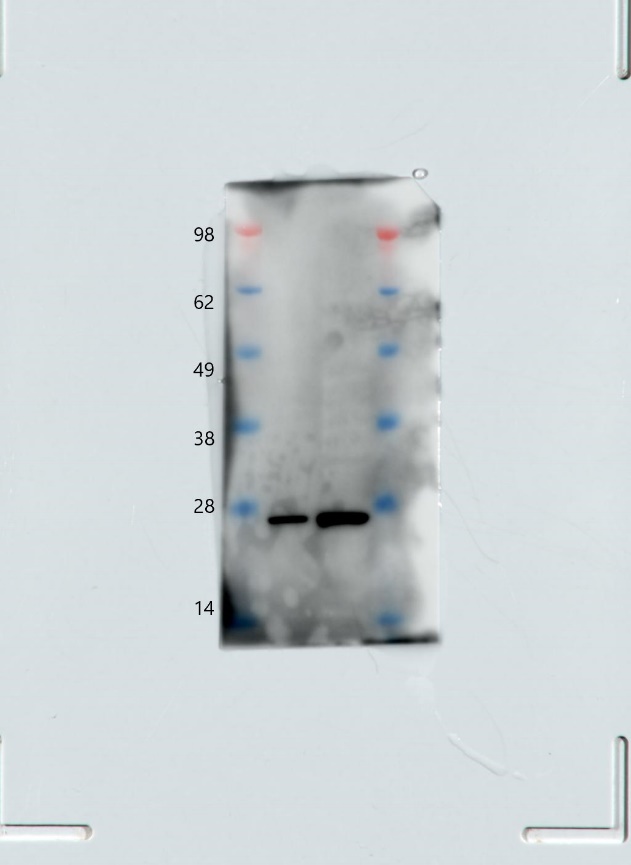
**

**p-Akt: 56 kDa**

**Akt: 56 kDa**

53

41

41

53

MSC

MSC+ Plasmid

MSC

MSC+ Plasmid

MSC+ Plasmid

MSC

MSC+ Plasmid

MSC

MSC

MSC+ Plasmid

**The Full-length blots/gels for Figure 7g -***CKAP4* CKD mice


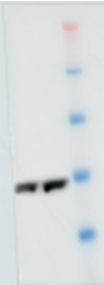

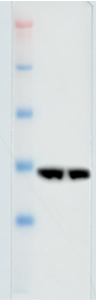

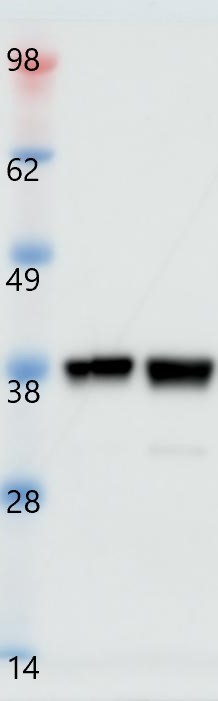

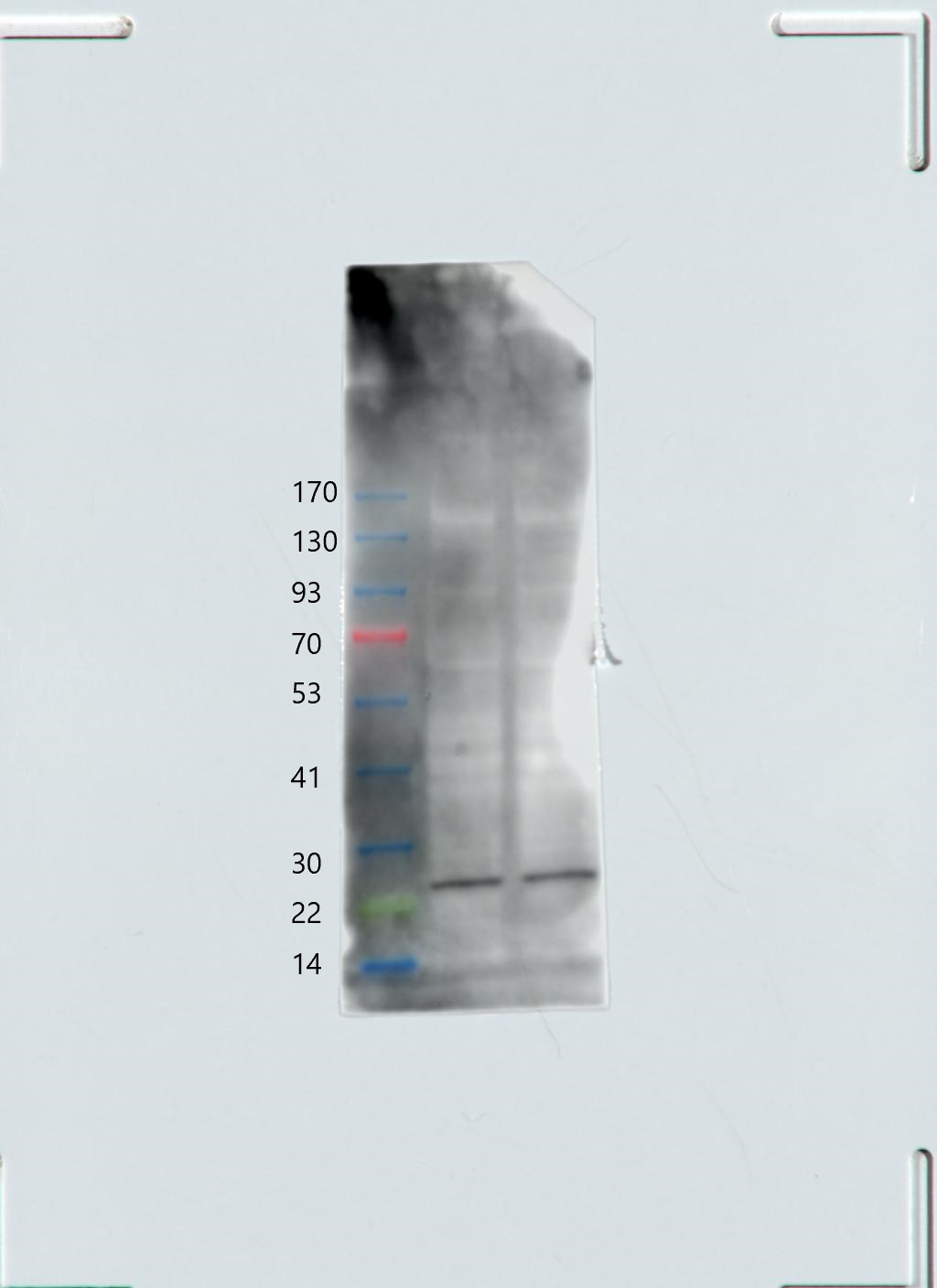

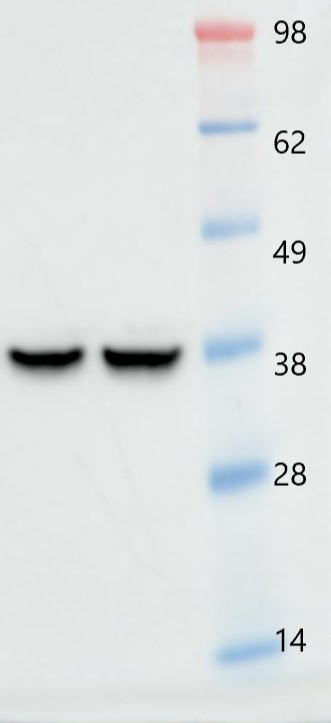


**Cyclin D1: 36 kDa**

**GADPH: 36 kDa**

**PCNA: 29 kDa**

MSC+ Plasmid

MSC

53

41

**p-Akt: 56 kDa**

**Akt: 56 kDa**

41

53

MSC

MSC+ Plasmid

MSC+ Plasmid

MSC

MSC+ Plasmid

MSC

MSC+ Plasmid

MSC

**The Full-length blots/gels for Figure 7g -***LRP6* CKD mice


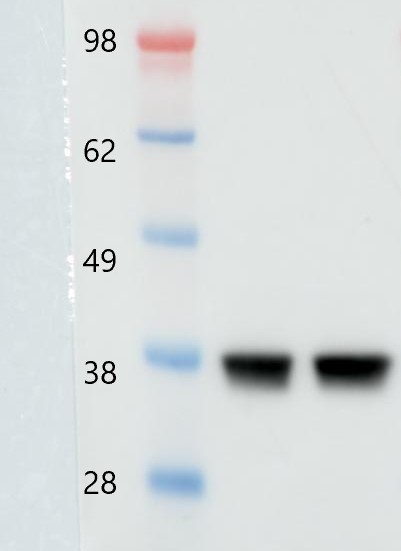

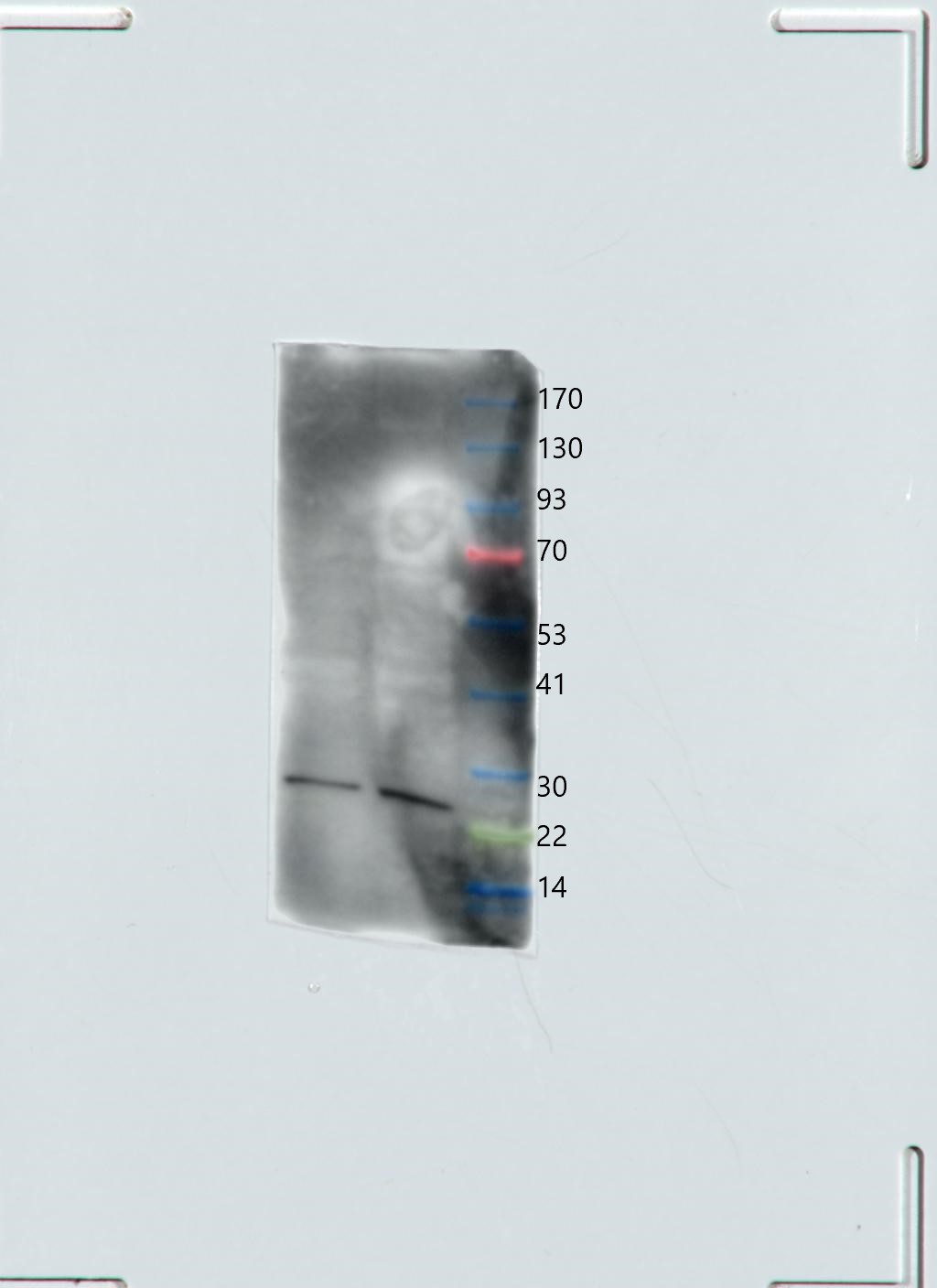

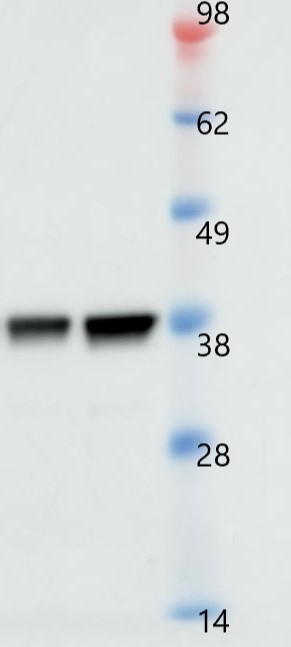


MSC+ Plasmid

MSC

MSC

MSC+ Plasmid

MSC

MSC+ Plasmid

MSC

MSC+ Plasmid

MSC

MSC+ Plasmid

**GADPH: 36 kDa**

**PCNA: 29 kDa**

**Cyclin D1: 36 kDa**


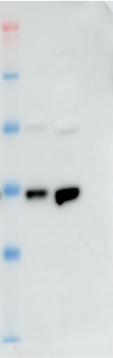

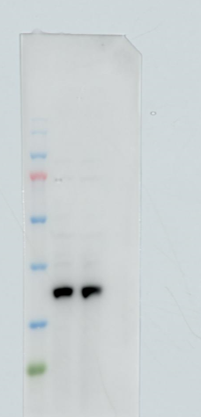


41

53

**Akt: 56 kDa**

**p-Akt: 56 kDa**

41

53
