# Supplementary material for: Invigorating human MSCs for transplantation therapy via Nrf2/DKK1 co-stimulation in an acute-on-chronic liver failure mouse model
Source: Gastroenterol Rep (Oxf). 2024 Mar 25;12:goae016. doi: 10.1093/gastro/goae016 (PMC10963075; doi:10.1093/gastro/goae016)
Supplement: goae016_Supplementary_Data [file goae016_supplementary_data.zip › hADMSC_product test report.pdf]

OriCell®

Product test report

OriCell®Adult Adipose-derived Mesenchymal Stem Cells

Product No.: HUXMD-01001

We help you discover life

Basic

Product name	OriCell®Adult Adipose-derived Mesenchymal Stem Cells
Item No	**HUXMD-01001**
Batch number	210311H61
Donor information	Male
Cryopreservation generation	**P2**
Save the conditions	Liquid nitrogen (-196 C°)

Test standard and result

Test items			Test results	Testing standards	Conclusion
Routine testing	Bacteria, fungi		Negative	Negative	Qualified
	Mycoplasma		Negative	Negative	Qualified
	Endotoxin		≤10EU	≤10EU	Qualified
Identification and testing	Resuscitation survival rate		96.03%	≥80%	Qualified
	Number of living cells		1.45×10^6	$\geq1\times10^6$	Qualified
	Cell recovery and adherence rate		98.17%	≥80%	Qualified
	Growth status		The population doubling time was 24.66 H.	Morphology is long fusiform, showing polar arrangement; Population doubling time ≤ 72 H	Qualified
	Differentiation ability		It can differentiate into adipogenic, osteogenic and chondrogenic cells.	It can differentiate into osteoblasts, adipocytes and chondroblasts by directional induction.	Qualified
	Surface marker molecules	**CD105**	95.73%	≥70%	Qualified
		**CD29**	99.97%	≥70%	Qualified
		**CD73**	99.98%	≥70%	Qualified
		**CD34**	0.96%	≤5%	Qualified
		**CD45**	0.20%	≤5%	Qualified
		**CD11b**	0.15%	≤5%	Qualified

Basic

		**CD44**	99.92%	≥70%	Qualified

Cell growth

Cell proliferation ability

The population doubling time was calculated to be 24. 66 H based on the data of logarithmic phase (1-3 days).

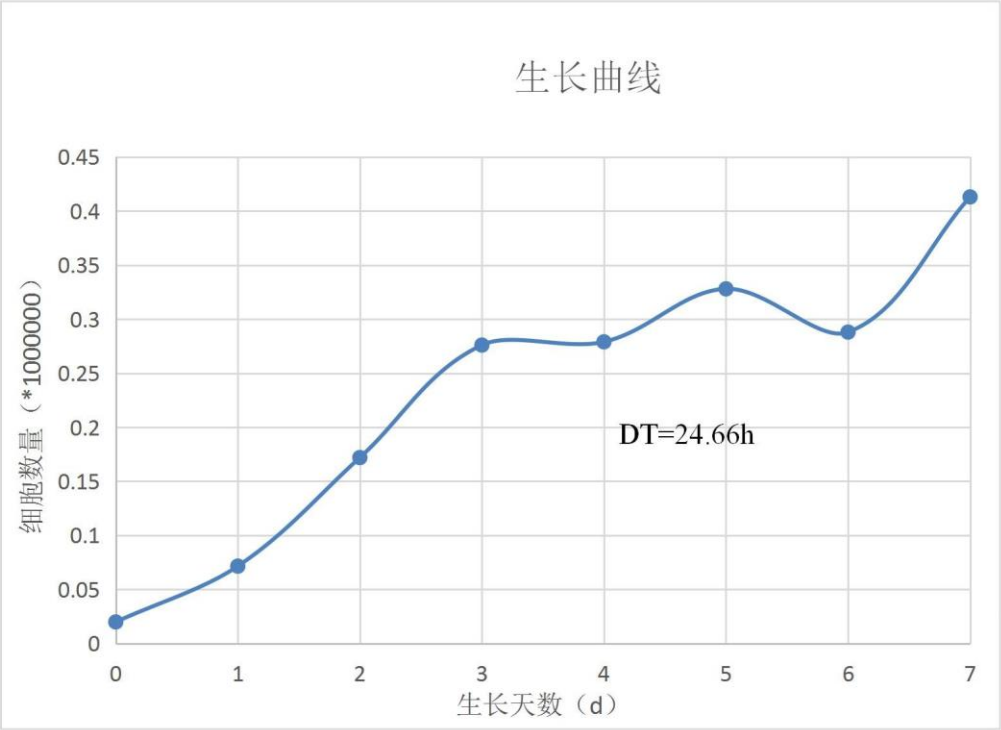

Cell passage ability

The cells grew in long fusiform shape with good polarity and stereo perception. After 5 passages, the cells were still viable.

Morphology of this batch of cells after 48h of Pn + 1 generation growth

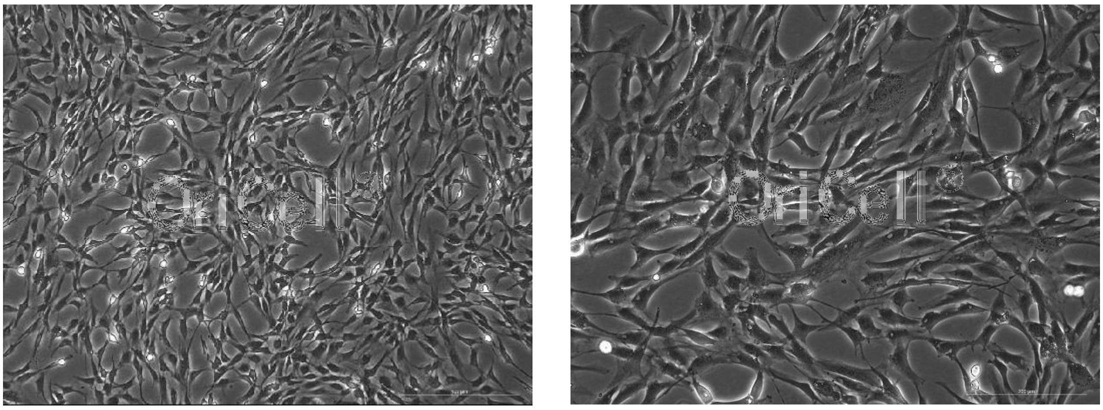

Morphology of this batch of cells after 72 hours of Pn + 3 generation growth

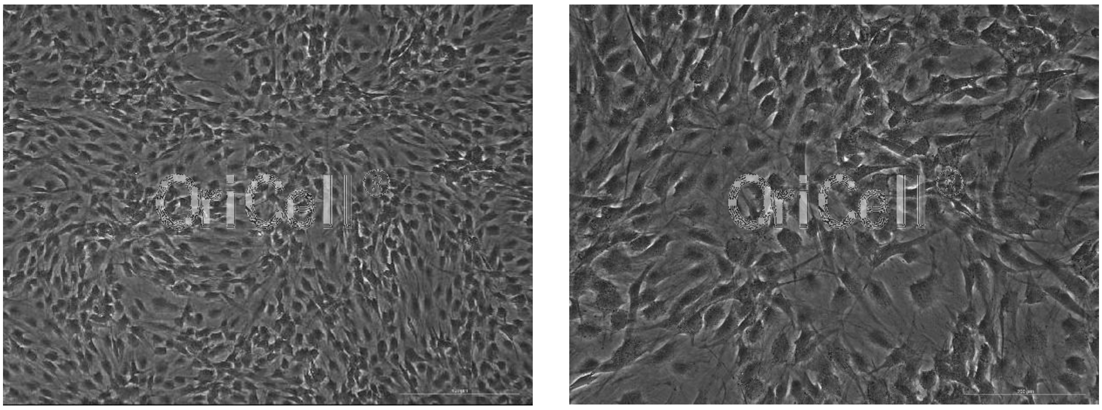

Morphology of this batch of cells after 48 hours of Pn + 5 generation growth

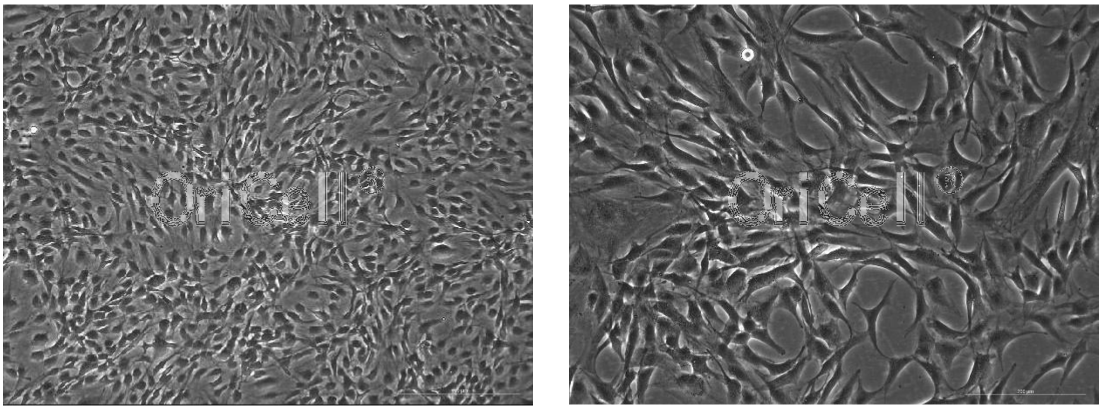

Ability to induce differentiation

Adipogenic differentiation

When the confluence of cells was about 90%, mesenchymal stem cell adipogenic induction solution was added. After 10 days, oil red O staining was carried out, and standard red lipid droplets could be seen.

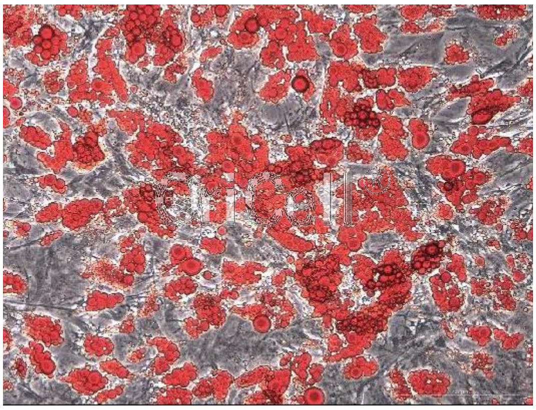

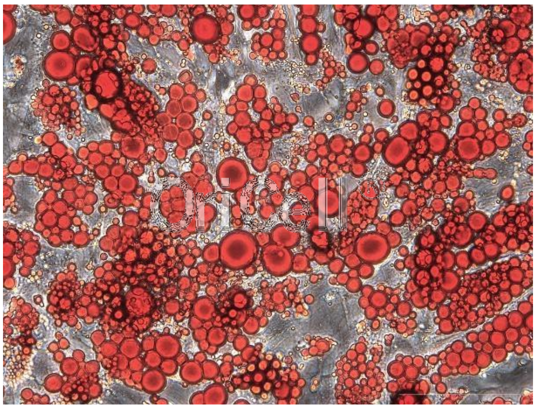

Osteogenic differentiation

Mesenchymal stem cell osteogenic induction solution was added when the confluence of cells reached about 70%. Alizarin red staining was performed 25 days later. Alizarin red combined with osteoid to form concentric dark red nodules.

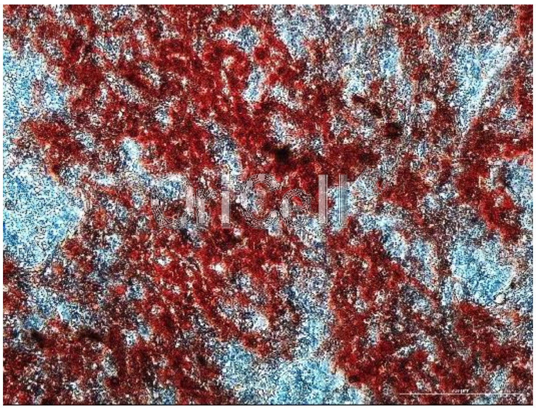

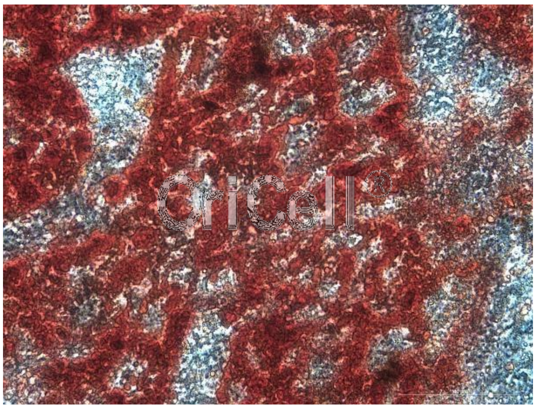

Chondrogenic differentiation

The cells were cultured by pellet culture and mesenchymal stem cell cartilage induction culture. The cells gradually changed from a flat cell group attached to the bottom of the centrifuge tube to a cell ball. After 21 days of induction, the cell group became larger and round, and the surface became smooth.

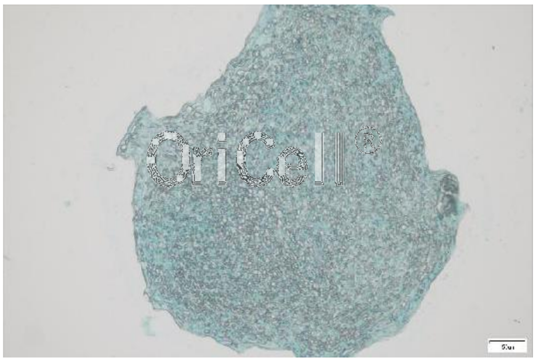

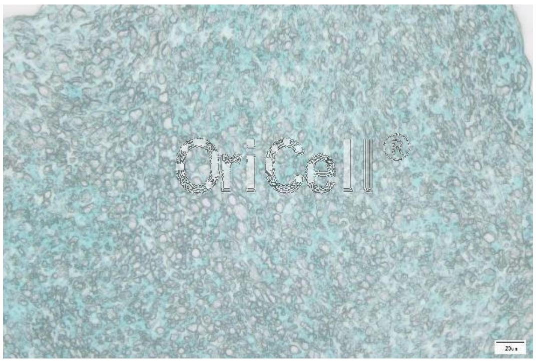

Surface molecular flow detection

Data details

Mouse IgG1, κ Isotype Control Antibody

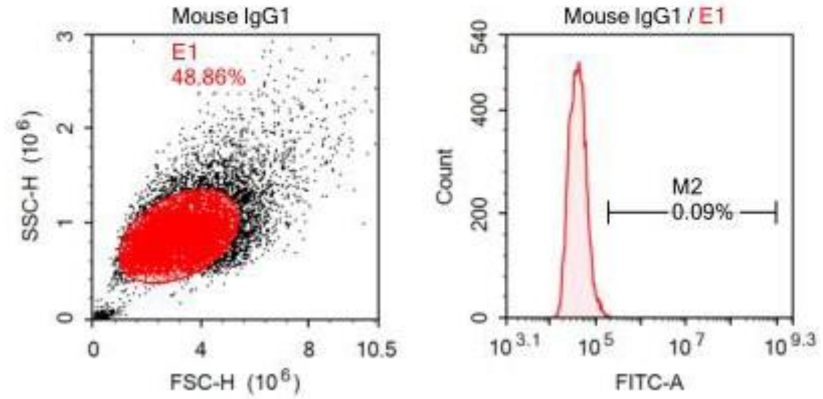

Anti-human CD105 antibody

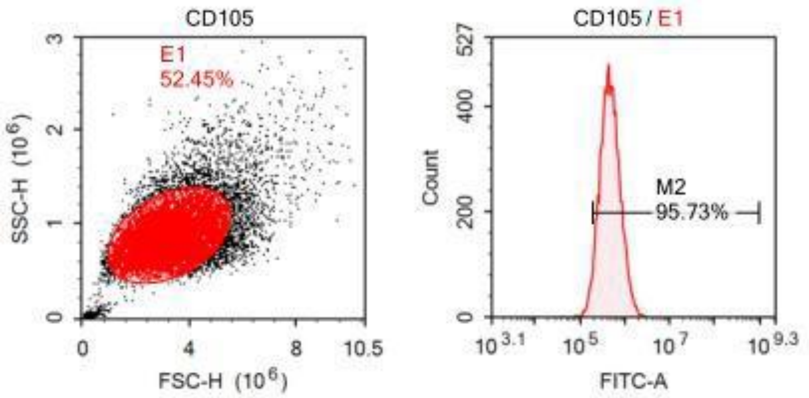

Anti-human CD29 antibody

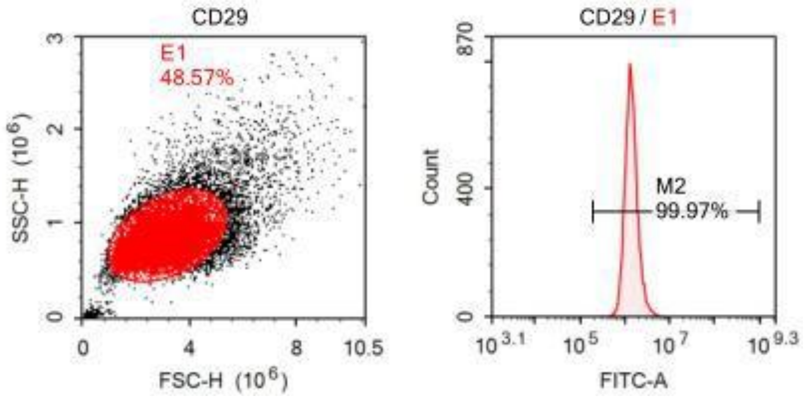

Anti-human CD73 antibody

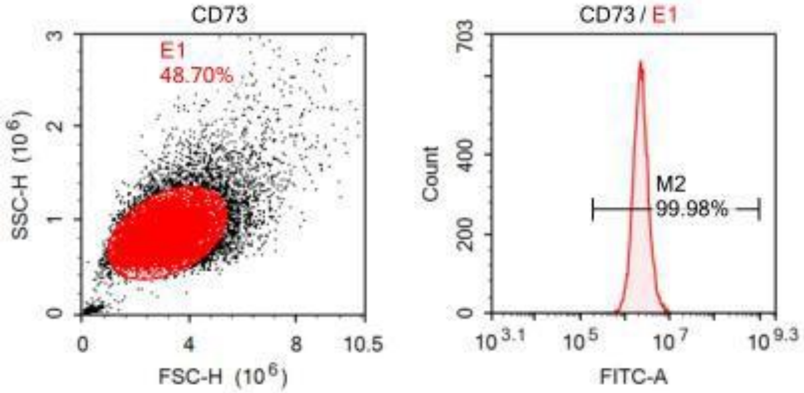

Anti-human CD34 antibody

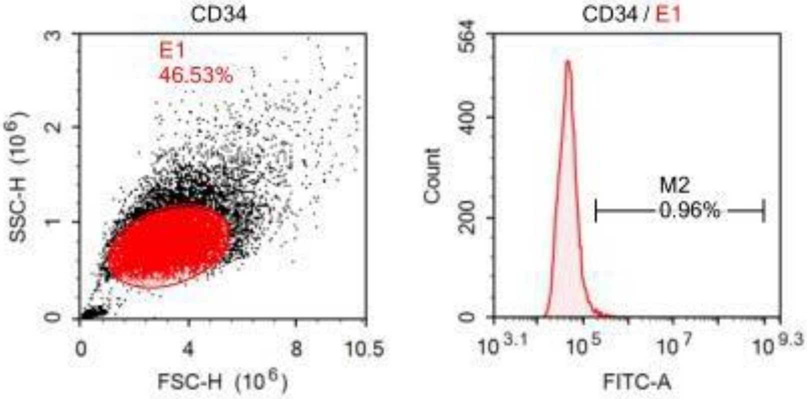

Anti-human CD45 antibody

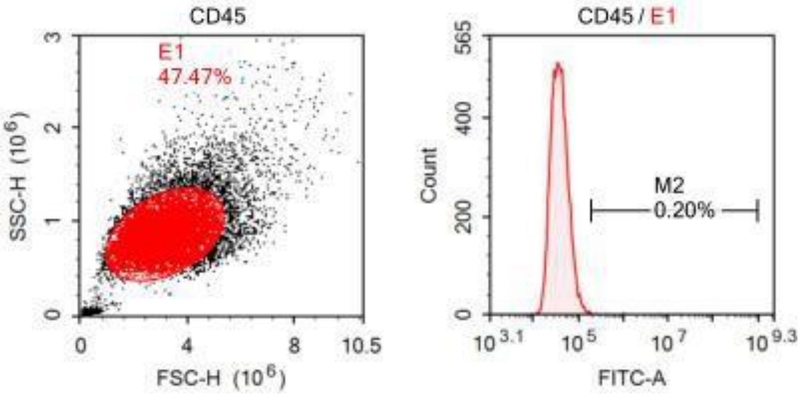

Anti-human CD11b antibody

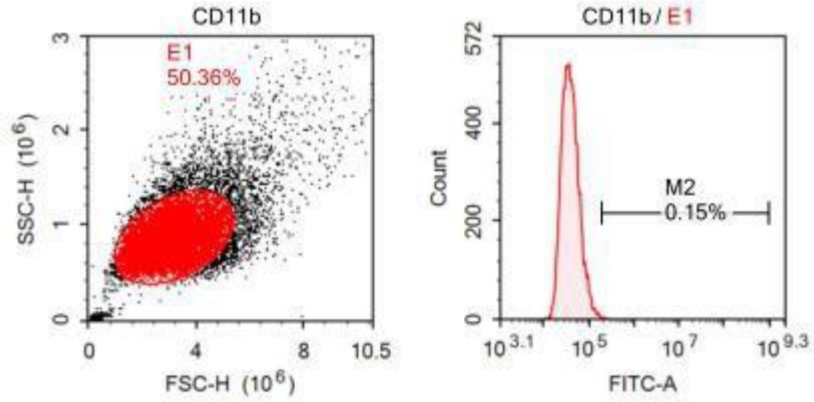

Rat IgG2b, κ Isotype Control Antibody

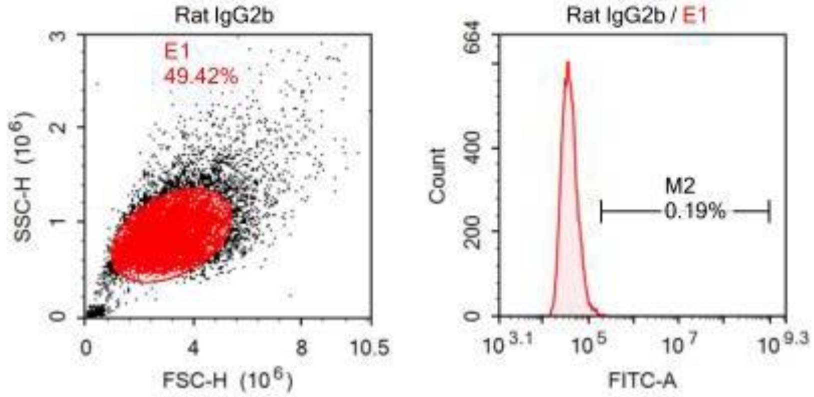

Anti-human CD44 antibody

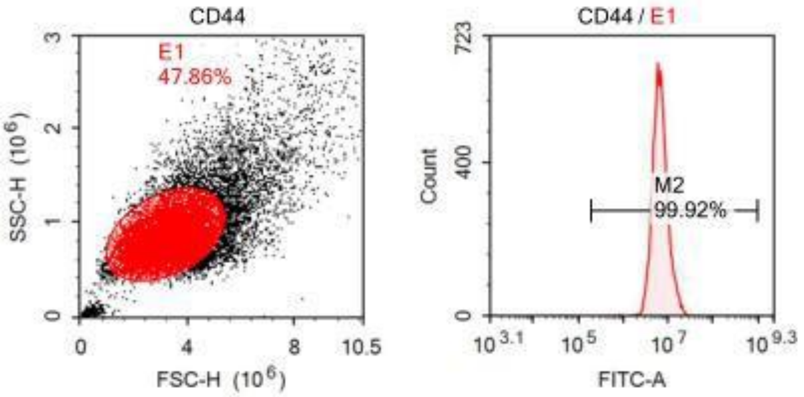

Conclusion

This product has been tested to meet the requirements of various indicators and is approved for release.

Inspector: Nancy

Reviewed by: Glory

Saiye (Guangzhou) Biotechnology Co., Ltd. reserves all rights to the technical documentation of OriCell®cell culture products. Any part of this document without the written permission of Saiye (Guangzhou) Biotechnology Co., Ltd.

Not to be adapted or reproduced for other commercial purposes.
