# Supplementary material for: Invigorating human MSCs for transplantation therapy via Nrf2/DKK1 co-stimulation in an acute-on-chronic liver failure mouse model
Source: Gastroenterol Rep (Oxf). 2024 Mar 25;12:goae016. doi: 10.1093/gastro/goae016 (PMC10963075; doi:10.1093/gastro/goae016)
Supplement: goae016_Supplementary_Data [file goae016_supplementary_data.zip › Supplementary_file_1_v3.docx]

**Invigorating human MSCs for transplantation therapy via Nrf2/DKK1 co-stimulation in a mice acute-on-chronic liver failure model**

**Running title:** Nrf2/DKK1 costimulation using MSCs in ACLF

Feng Chen^1,2†^, Zhaodi Che^3†^, Yingxia Liu^2^, Pingping Luo^3^, Lu Xiao^3^, Yali Song^3^, Cunchuan Wang^3^, Zhiyong Dong^3^, Mianhuan Li^2^, George L. Tipoe^4^, Min Yang^2^, Yi Lv^5^, Hong Zhang^6^, Fei Wang^1*^, and Jia Xiao^3,6,7*^

^1^Division of Gastroenterology, Seventh Affiliated Hospital of Sun Yat-sen University, Shenzhen, Guangdong, P. R. China

^2^National Clinical Research Center for Infectious Diseases, Second Affiliated Hospital of Southern University of Science and Technology, Shenzhen, Guangdong, P. R. China

^3^Clinical Medicine Research Institute and Department of Metabolic and Bariatric Surgery, The First Affiliated Hospital of Jinan University, Guangzhou, Guangdong, P. R. China

^4^School of Biomedical Sciences, The University of Hong Kong, Hong Kong SAR, P. R. China

^5^Laboratory of Neuroendocrinology, Fujian Key Laboratory of Developmental and Neurobiology, School of Life Sciences, Fujian Normal University, Fuzhou, Fujian, P. R. China.

^6^Department of Surgery, The Sixth Affiliated Hospital of Jinan University, Jinan University, Dongguan, Guangdong, P. R. China

^7^Shandong Provincial Key Laboratory for Clinical Research of Liver Diseases, Qingdao Hospital, University of Health and Rehabilitation Sciences, Qingdao, Shandong, P. R. China.

^†^These authors contributed equally to this work.

**Correspondence**

*Jia Xiao
Clinical Medicine Research Institute and Department of Metabolic and Bariatric Surgery, The First Affiliated Hospital of Jinan University, 613 Huangpu Avenue West, Guangzhou, Guangdong 510632, P. R. China.
Tel: +86-18520228386; Fax: +86-18520228386;
Email: [edwinsiu@connect.hku.hk](mailto:edwinsiu@connect.hku.hk) (J.X.)

*Fei Wang
Division of Gastroenterology, Seventh Affiliated Hospital of Sun Yat-sen University, 628 Zhenyuan Road, Shenzhen, Guangdong 518107, P. R. China.
Tel: +86-13816601005; Fax: +86-0755-81206211;
Email: [wangf323@mail.sysu.edu.cn](mailto:wangf323@mail.sysu.edu.cn) (F.W.)

**Supplementary Figure 1**

**
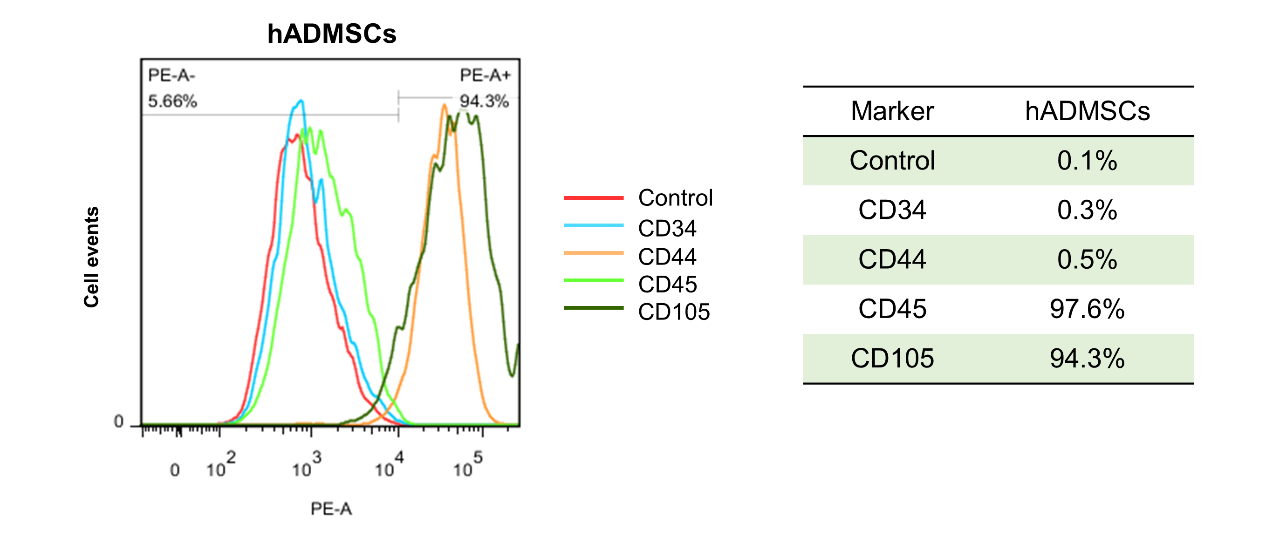
**

**Supplementary Figure 1.** **Validation of cell surface markers.** Flow cytometry analysis on human adipose-derived mesenchymal stromal cells (hADMSCs) showed their high expression in CD44 and CD105 and low expression in CD34 and CD45

**Supplementary Figure 2**

**
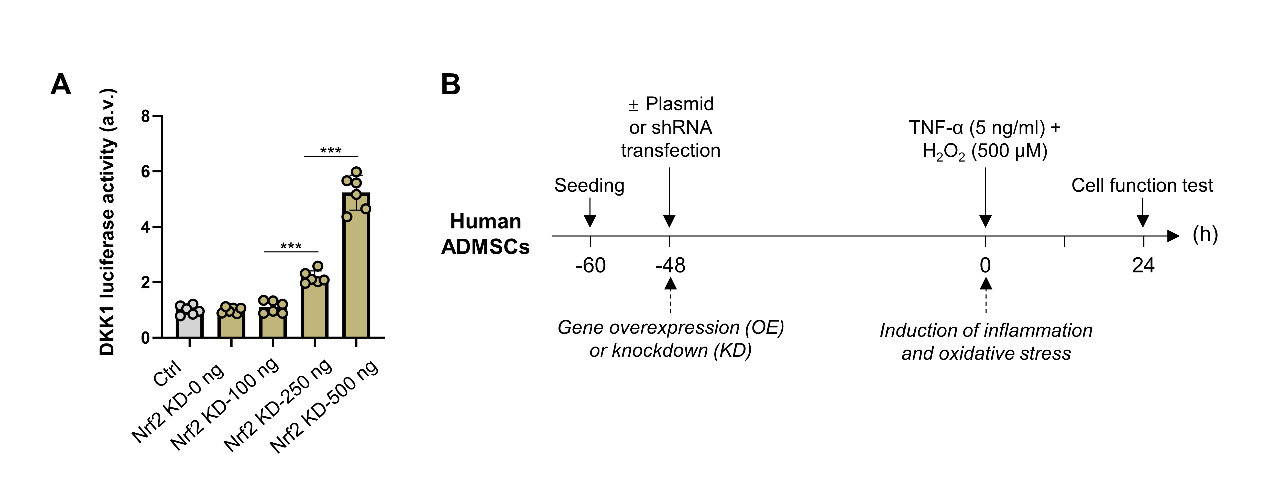
**

**Supplementary Figure 2. The Nrf2 promoter directly regulates DKK1.** (A) Changes of DKK1 luciferase activity when Nrf2 was knocked-down by siRNA with indicated concentrations (*n* = 6). (B) Experimental design illustration showing that Nrf2 and DKK1 was overexpressed (OE) or knocked-down (KD) by transfection with gene open reading frame-bearing plasmid or shRNA, respectively, at the time of 48-h before the treatment of TNF-α/H_2_O_2_ in MSCs. Data are expressed as mean ± SD. *** indicates *P* < 0.001 against the control group

**Supplementary Figure 3**

**
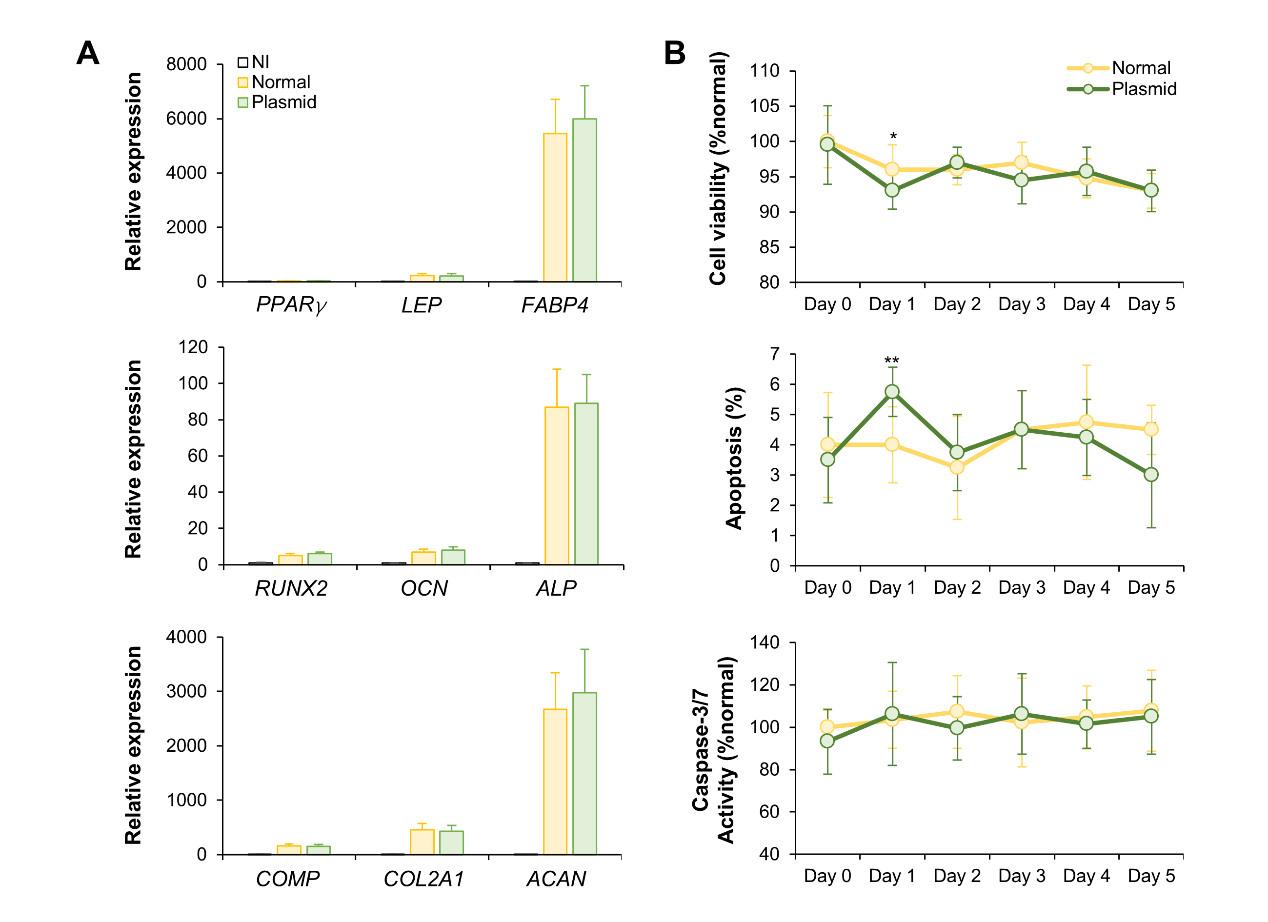
**

**Supplementary Figure 3.** **Transfection with a pIRES2-Nrf2-DKK-1 plasmid does not interfere with MSCs transdifferentiating potential or cell status.** (A) Quantitative RT-PCR measurements of key genes for MSCs adipogenic, osteogenic, and chondrogenic differentiation. (B) Changes in MSCs viability, apoptosis ratios, and caspase-3/7 activities on day 0-5 following transfection with the pIRES2-Nrf2-DKK1 plasmid. All data shown herein are that of MSCs. Data are expressed as mean ± SD. *, ** indicate *P* < 0.05, 0.01 against a corresponding untreated MSCs group, respectively

**Supplementary Figure 4**

**
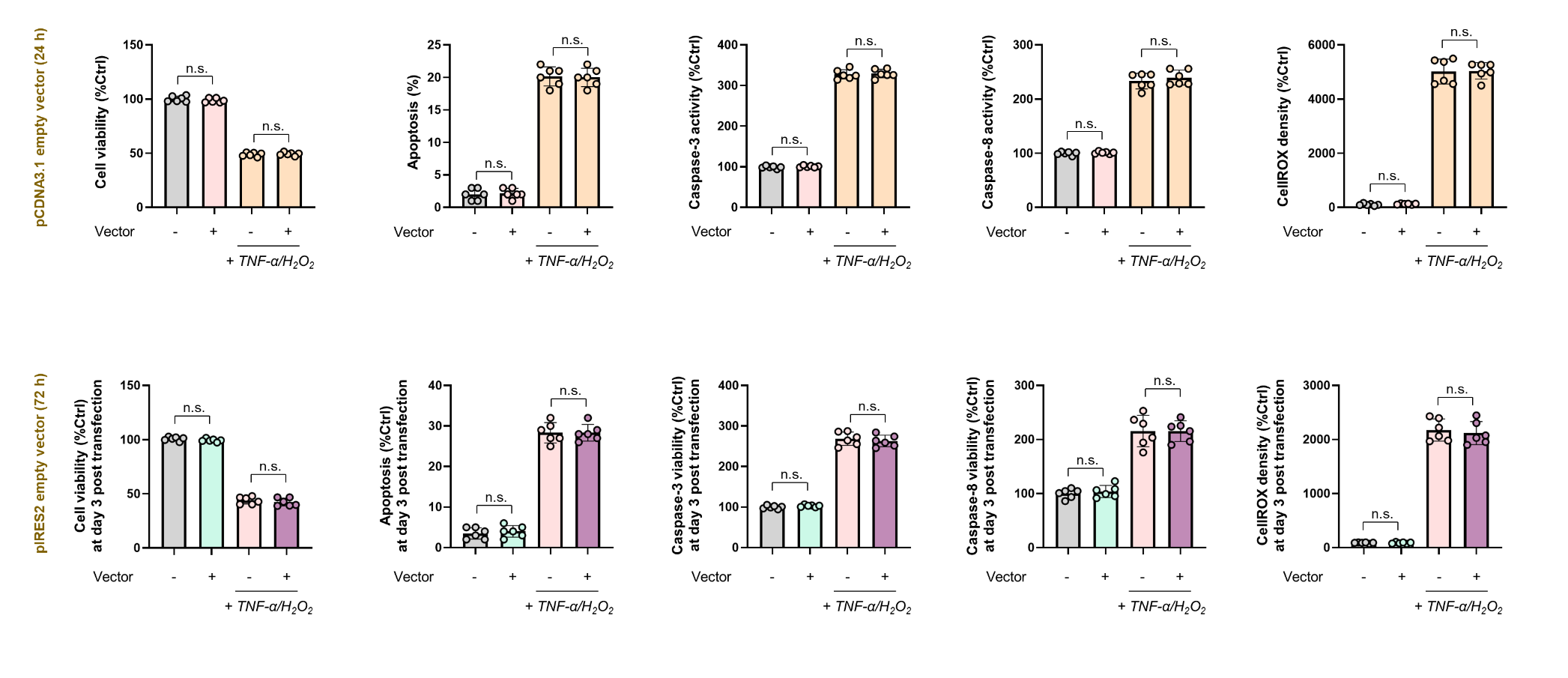
**

**Supplementary Figure 4. Empty vector does not influence stem cell states.** Transfection of pCDNA3.1 (24 h) or pIRES2 (72 h) empty vector into human MSCs did not influence cell viability, apoptotic ratio, activity of caspase-3/8, and CellROX density. Data are expressed as mean ± SD. Results are representative of at least 3 independent experiments. n.s. not significant

**Supplementary Figure 5
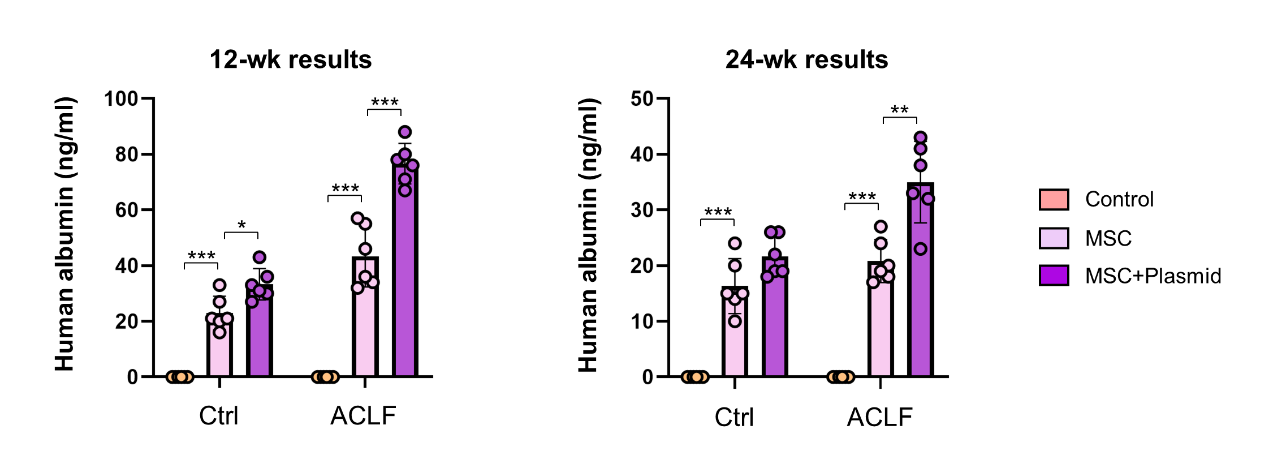
**

**Supplementary Figure 5. Long-term (12- and 24-week) donor cell function in healthy and ACLF mice transplanted with human MSCs with or without plasmid pre-transfection.** Human albumin was determined in the serum by an ELISA assay at weeks 12 and 24 for healthy or ACLF mice (*n* = 6) transplanted with preconditioned MSCs. Mice without MSC transplantation served as negative controls. Data are expressed as mean ± SD. Results are representative of at least 3 independent experiments. *, **, *** represent *P* < 0.05, 0.01, 0.001 between indicated groups

**Supplementary Table 1. Sequences of Plasmid Constructs.**

| Entry | Plasmid construct | Vector | Insert | Accession number |
| --- | --- | --- | --- | --- |
| 1 | pCDNA3.1 | N.A. | Nil | N.A. |
| 2 | pIRES2-EGFP | pIRES2 | EGFP (Clontech) | N.A. |
| 3 | pCDNA3.1-Nrf2 | pCDNA3.1 | Nrf2 (full length) | NM_006164 |
| 4 | pCDNA3.1-DKK-1 | pCDNA3.1 | DKK-1 (full length) | NM_012242 |
| 5 | pCDNA3.1-Nrf2-DKK-1 | pCDNA3.1-DKK-1 | Nrf2 (full length) | As above |
| 6 | pIRES2-Nrf2-DKK-1 | pIRES2 | Nrf2-DKK-1 (full length) | As above |

**Supplementary Table 2.** **Tumor incidence rate after transplantation of human adipose-derived mesenchymal stromal cells (hADMSCs) in healthy and acute-on-chronic liver failure (ACLF) mice.**

| Group | Test item | Injected cell amount | No. of tumor bearing mice^a^ | Tumor incidence rate (%) |
| --- | --- | --- | --- | --- |
| Healthy mice | MRC-5^b^ | 1 × 10^7^ | 0/12 | 0 |
|  | hADMSCs | 1 × 10^7^ | 0/12 | 0 |
|  | hADMSCs with plasmid | 1 × 10^7^ | 0/12 | 0 |
|  | ES-D3^d^ | 1 × 10^7^ | 10/10 | 100 |
| ACLF model | MRC-5 | 1 × 10^7^ | 0/9 | 0 |
|  | hADMSCs | 1 × 10^7^ | 0/11 | 0 |
|  | hADMSCs with plasmid | 1 × 10^7^ | 0/14 | 0 |
|  | ES-D3 | 1 × 10^7^ | 9/9 | 100 |

^a^Dead mice during experiments were not included for calculations.

^b^Negative control group.

^c^Transfected with the pIRES2-Nrf2-DKK1 plasmid for 24 h before MSCs transplantation.

^d^Positive control group.
