# Supplementary figures and images for: Invigorating human MSCs for transplantation therapy via Nrf2/DKK1 co-stimulation in an acute-on-chronic liver failure mouse model
Source: Gastroenterol Rep (Oxf). 2024 Mar 25;12:goae016. doi: 10.1093/gastro/goae016 (PMC10963075; doi:10.1093/gastro/goae016)

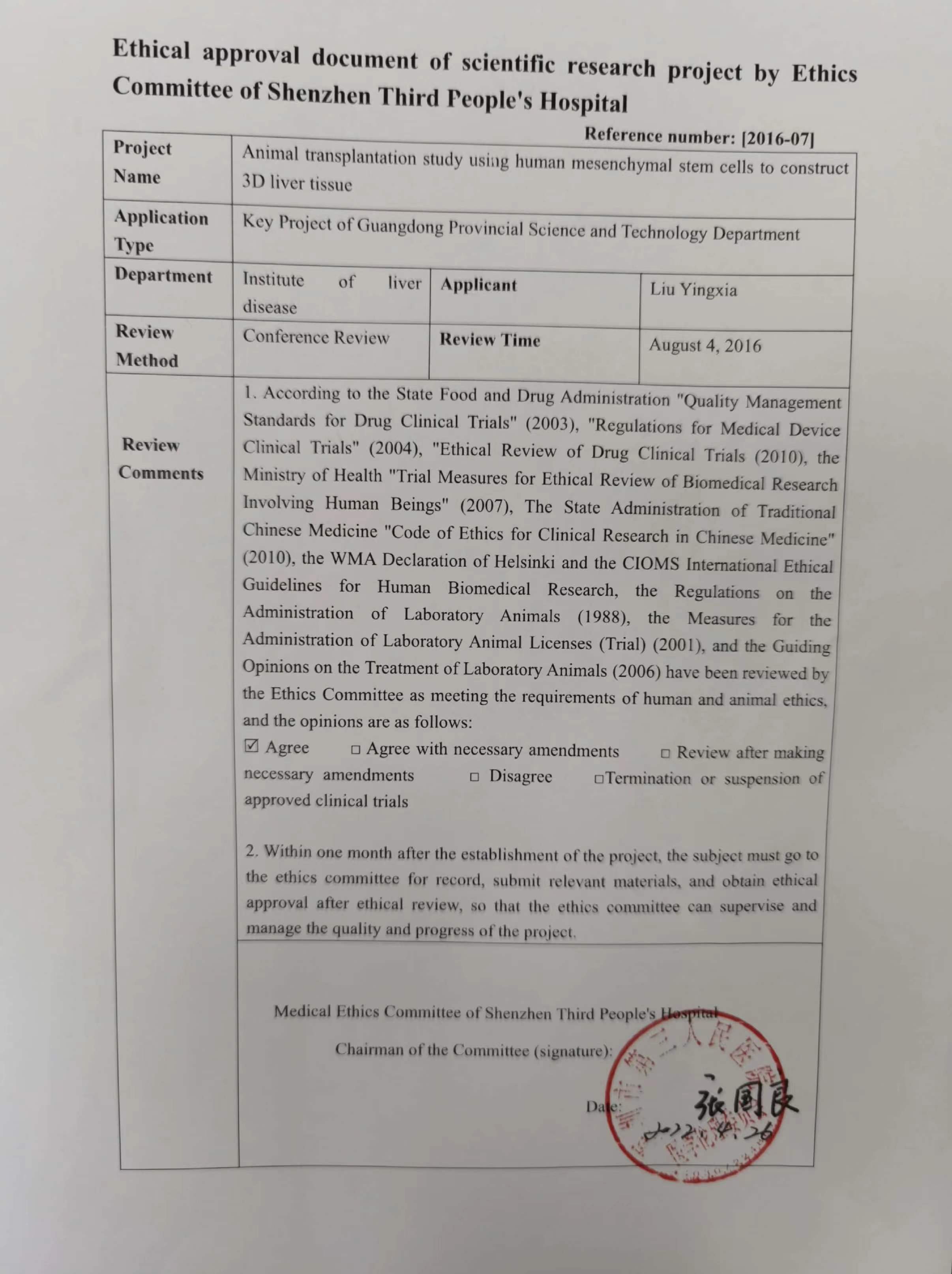

Supplement: goae016_Supplementary_Data [file goae016_supplementary_data.zip › Ethics Statement.jpg]
